# UPLC-MS/MS High-Risk Screening for Sphingolipidoses Using Dried Urine Spots

**DOI:** 10.3390/biom14121612

**Published:** 2024-12-17

**Authors:** Tristan Martineau, Bruno Maranda, Christiane Auray-Blais

**Affiliations:** Division of Medical Genetics, Department of Pediatrics, Faculty of Medicine and Health Sciences, Université de Sherbrooke, Centre de Recherche-CHUS, 3001, 12th Avenue North, Sherbrooke, QC J1H 5N4, Canada; tristan.martineau@usherbrooke.ca (T.M.); bruno.maranda@usherbrooke.ca (B.M.)

**Keywords:** tandem mass spectrometry, urine dried on filter paper, lysosomal storage disorders, lysosphingolipids, sphingolipidoses, Fabry disease, Gaucher disease

## Abstract

Background: Early detection of sphingolipidoses is crucial to prevent irreversible complications and improve patient outcomes. The use of urine samples dried on filter paper (DUS) is a non-invasive strategy that simplifies the collection, storage, and shipping of samples compared to using liquid urine specimens. Objectives: (1) Develop and validate a multiplex ultra-performance liquid chromatography–tandem mass spectrometry (UPLC-MS/MS) methodology using DUS to quantify twenty-one lysosphingolipids normalized to creatinine for eight different sphingolipidoses. (2) Establish normal reference values to evaluate the clinical utility of the methodology. Methods: Samples were eluted from a 5 cm filter paper disk (~1 mL of urine) and extracted on Oasis MCX solid-phase extraction cartridges prior to injection in the UPLC-MS/MS system. Results: Urinary lysosphingolipids were stable on DUS at −80 °C and −30 °C for 117 days, at 21.5 °C and 4 °C for at least 26 days, and at 35 °C for 3 days. Globotriaosylsphingosine, glucosylsphingosine, and their analogs were elevated in patients with Fabry disease and Gaucher disease, respectively, compared to controls (*p*-value < 0.0001). The analysis of related analog profiles suggests a better overall reliability in detecting patients early, especially for Fabry patients. Conclusions: This approach is feasible and might be useful for the early detection, monitoring, and follow-up of patients with sphingolipidoses.

## 1. Introduction

Lysosomal storage disorders (LSDs) regroup over 70 inborn errors of metabolism characterized by the excess systemic accumulation of metabolites due to mutated genes encoding lysosomal enzymes, transporters, or enzyme cofactors [1].

Sphingolipidoses, an LSD subgroup, represent several rare diseases caused by a dysfunctional pathway of sphingolipid catabolism. Sphingolipids form an essential lipid class regarding the cellular structure, homeostasis, adhesion, signaling, senescence, development, and death. Their chemical structure is based on a sphingosine backbone comprised of a N-acylated fatty acid with a variable chain length (isoforms) or modifications on the sphingosine moiety (analogs) combined with a carbohydrate group (cerebrosides and gangliosides) or hydrogen (ceramide) in the C-1 hydroxyl [2,3,4,5,6,7,8].

The accumulation of a specific sphingolipid leads to different diseases, such as metachromatic leukodystrophy, Gaucher disease, Krabbe disease, Fabry disease, GM1 and GM2 gangliosidoses, and Niemann–Pick disease. They are autosomal recessive genetic disorders, except for Fabry disease, which is X-linked [9].

Gaucher disease (OMIM no. 230800) has an average incidence of 1 in 50,000 births but is more prevalent in some populations, such as the Ashkenazi Jewish population (1 in 800). It is caused by mutations in the *GBA1* gene (chromosome 1 [1q21]), leading to the deficiency of the glucocerebrosidase enzyme (EC: 4.2.1.25), which is essential to the catabolism of glucosylceramide [10]. The cellular accumulation of metabolites, including glucosylceramide, glucosylsphingosine and related analogs in several tissues (bone marrow, spleen, and liver) and biological fluids (urine and plasma) will cause cellular dysfunctions and organ involvement [6,7,11]. There are three major Gaucher disease clinical types (1, 2, 3) and two variant phenotype forms (perinatal-lethal and cardiovascular). Type 1 is the most common and is characterized by several clinical manifestations, such as bone involvement, hepatosplenomegaly, anemia, thrombocytopenia, and lung disease. Compared to type 2 (acute; infantile) and type 3 (subacute; juvenile), Gaucher disease type 1 is non-neuronopathic [10].

Krabbe disease (OMIM no. 245200) is caused by mutations in the *GALC* gene and is characterized by progressive demyelination and the presence of globoid cells [12]. It is caused by the deficiency of galactosylceramidase (EC: 3.2.1.46), which causes the accumulation of galactosylceramide and galactosylsphingosine and leads to the degeneration of oligodendrocytes. These lipids are important for the integrity of the myelin, kidney, and epithelial cells of the small intestine and the colon [12,13]. Elevated levels of galactosylsphingosine in dried blood spots were previously detected [14].

Fabry disease (OMIM no. 301500) is a multisystemic X-linked sphingolipidosis caused by mutations in the *GLA* gene affecting the α-galactosidase A enzyme (EC: 3.2.1.22) activity, which leads to the accumulation of globotriaosylceramide (Gb_3_), globotriaosylsphingosine (lyso-Gb_3_), and their analogs/isoforms in biological fluids and tissues [5,8,15]. The severity and signs/symptoms are quite variable. The main features are acroparesthesia, angiokeratomas, corneal opacity, cornea verticillata, digestive tract issues and severe complications such as kidney disease, heart failure and stroke. Fabry females may also develop the disease, albeit generally with less severe symptoms compared to male patients [16].

Niemann–Pick disease types A and B (type A: OMIM no. 257200; type B: OMIM no. 607616) are characterized by the deficiency of acid sphingomyelinase (EC: 3.1.4.12), which leads to lipid storage accumulation and to visceral or neurovisceral manifestations. Intermediate forms (types A and B) are caused by mutations in the *SMPD1* gene. Niemann–Pick disease type C (OMIM no. 257220) is a less severe form resulting from mutations in the *NPC1* or *NPC2* gene and affecting the cholesterol trafficking without altering the enzyme activity of acid sphingomyelinase [17]. The clinical manifestation spectrum largely depends on the disease form, but patients may suffer from common neurological signs and symptoms such as gaze palsy, cerebellar ataxia, dysarthria, cataplexy, seizures, dystonia, and progressive dementia [17].

Metachromatic leukodystrophy (OMIM no. 250100) is the most common leukodystrophy, with a prevalence of 1 in 40,000–160,000 worldwide. Due to the deficiency of the lysosomal enzyme arylsulfatase A (EC: 3.1.6.1), sulfatides and sphingolipids accumulate in affected cells and damage the myelin sheath of the central and peripheral nervous systems, causing progressive motor and cognitive involvement [18].

GM1 gangliosidosis (OMIM no. 230500) is caused by mutations in the *GLB1* gene (chromosome 3p21.33), leading to the deficiency of β-galactosidase activity (EC: 3.2.1.23), while GM2 gangliosidosis (Tay–Sachs disease: OMIM no. 272800; Sandhoff disease: OMIM no. 268800) is characterized by mutations in the *HEXA* or *HEXB* gene, respectively, leading to the deficiency of β-hexosaminidase A and B (EC: 3.2.1.52). GM1 gangliosidosis is distinguished by the systemic and central nervous system neurodegeneration phenotype with a severity spectrum categorized as type I (infantile), type II (late infantile), and type III (juvenile). GM2 gangliosidosis is characterized only by central nervous system involvement [19].

There is no treatment available to cure these diseases. However, several approaches were developed for patients affected by sphingolipidoses to reduce or stop disease progression: enzyme replacement therapy (ERT) for Gaucher disease [20] and Fabry disease [21], substrate reduction therapy (SRT) for Gaucher disease [20] and Niemann–Pick C [22], pharmacological chaperone therapy for Gaucher disease [20] and Fabry disease [21], gene therapy for Fabry disease [21,23,24], Gaucher disease [20] and metachromatic leukodystrophy [25], and allogeneic hematopoietic stem cell transplantation for Gaucher disease [20], Krabbe disease [26], and metachromatic leukodystrophy [18]. There is no FDA-approved therapy option for GM1 and GM2 gangliosidoses. Genome editing by CRISPR-Cas9 is an emerging therapy currently in progress and seems promising in terms of curing sphingolipidoses [27]. Biomarker monitoring is important at various stages of therapy development to evaluate the safety and the therapeutic efficiency [28].

Early detection of these disorders using biomarkers is important to treat affected patients in a window of action before the development of irreversible organ damage [29,30]. Newborn screening programs (NBSs) were developed to reduce the diagnostic delays experienced by LSD patients [31,32]. Currently, the gold standard is to measure specific enzyme activities from dried blood spots (DBSs), followed by confirmation tests such as clinical evaluations, enzyme activity measurements in leukocytes/lymphocytes, biomarker quantitation, and mutation analyses [31,32,33]. However, analytical challenges remain. The analytical accuracy when using DBS can be affected by the limited volume of blood available on the filter paper and the hematocrit effect [34]. Also, patients with late-onset forms may have residual enzyme activity that is not correlated with the genotype and phenotype [31,35,36]. A high number of patients with variants of uncertain significance (VUS) are detected in NBSs [31,36]. It has been reported that among 248,616 newborns screened in the NBS program of Northeast Italy, 22 of 126 confirmed cases were asymptomatic with VUS (3 Gaucher disease, 7 Pompe disease, 10 Fabry disease, 2 mucopolysaccharidosis type I) [31]. Furthermore, even if NBS programs allow early detection of patients, the most appropriate course of action for the follow-up, management, and treatment of these patients still needs to be confirmed [31]. Guidelines for clinicians are updated frequently. Recent expert consensus guidelines recommend the monitoring of specific biomarkers, such as lyso-Gb_3_ instead of Gb_3_ in plasma, for the evaluation of the pharmacodynamic response and treatment outcomes in patients with the classic form of Fabry disease. Nevertheless, detection of late-onset forms or female Fabry patients is still challenging [37].

Lysosphingolipids, which represent deacylated sphingolipids generated by the acid ceramidase from intra-lysosomal sphingolipid accumulations, are interesting biomarkers for the follow-up and monitoring of LSDs [38,39]. As mentioned above, analog forms of primary lysosphingolipids were found in plasma and urine samples from Fabry disease [5,8] and Gaucher disease patients [6,7]. In urine samples, the analog levels may be more elevated than the primary lysosphingolipid itself. More studies analyzing larger cohorts of Gaucher and Fabry disease patients are needed to assess the clinical benefits of analyzing the analog profiles. Still, previous studies have shown the advantages of the analog profile analysis in Fabry patients with late-onset mutations [40,41].

Several analytical MS methods were developed to analyze different lysosphingolipids in tissues and biological fluids, especially in plasma samples [42,43,44]. Some methodologies were developed to analyze the lysosphingolipid profiles using DBSs [45,46]. Some analytical MS methods were developed using urine samples from patients with metachromatic leukodystrophy [47], GM1 gangliosidosis [48], GM2 gangliosidosis [49], and Niemann–Pick disease [50]. We have previously shown that LC-MS sensitivity can ensure the detection and measurement of sphingolipids and lysosphingolipids in urine, including in Gaucher disease and Fabry disease [51,52]. We have already developed a multiplex UPLC-MS/MS methodology to measure lysosphingolipids in liquid urine specimens for the detection of the aforementioned sphingolipidoses, including Krabbe disease [53]. Compared to plasma, it remains true that urine sample collection is non-invasive for patients and can easily be performed at home, but the number of studies using urine samples for analysing lysosphingolipids is limited. Also, home collection of liquid urine samples from the pediatric population can still be tedious, according to many parents [54].

Therefore, a urine filter paper collection approach can be readily used. Existing programs, such as the Provincial Neonatal Urine Screening Program in Sherbrooke, Quebec, Canada, have proven that using urine dried on filter paper allows the detection of inborn errors of metabolism (IEM) in newborns [55]. Until now, more than 3.7 million babies have been screened using DUSs collected at home by parents, with a voluntary compliance ranging from 84–93%. Also, the program has highlighted that it is possible to easily collect samples and analyze 5 cm DUS disks. More recently, a mass spectrometry approach was used to evaluate the feasibility of screening IEM using DUS specimens as part of a sensitive, specific, non-invasive, and low-cost strategy [56]. Furthermore, the DUS approach was previously used to collect LSD patient samples: mucopolysaccharidoses [57], Pompe disease [58], Fabry disease [59], and MLD [60]. Finally, considering that the concept of “hospital at home” and virtual medicine is more accessible than before [61], using DUSs to collect samples from patients may simplify the process, reduce the cost, and facilitate sample shipping by regular mail to the analytical laboratory [62,63].

Considering these previous studies, there is an incentive to develop a new multiplex non-invasive high-risk screening MS approach for the early detection, monitoring, and follow-up of sphingolipidosis-affected pediatric patients, as well as some adult patients, using DUS instead of liquid urine. Therefore, our main objectives were twofold: (1) to develop and validate a multiplex quantitative UPLC-MS/MS methodology using DUS to quantify the levels of 21 lysosphingolipids and their related analogs normalized to creatinine for the detection of eight different sphingolipidoses: metachromatic leukodystrophy, Gaucher disease, Krabbe disease, Fabry disease, GM1 gangliosidosis, GM2 gangliosidosis (Tay–Sachs and Sandhoff diseases) and Niemann–Pick disease; and (2) to establish normal reference values to evaluate the clinical utility of this assay for high-risk screening, monitoring, and follow-up of patients.

## 2. Materials and Methods

### 2.1. Ethics Approval

This research project followed the principles outlined in the Declaration of Helsinki for all human experimental investigations and was approved by the Research Ethics Board at the Centre intégré universitaire de santé et de services sociaux de l’Estrie—Centre hospitalier universitaire de Sherbrooke under project ID 2023-4955, which was an extended study of project ID 2021-3435. Urine samples were obtained from consenting participants as part of this latter study [53].

### 2.2. Urine Sample Collection

Urine samples selected from healthy controls (n = 59), patients diagnosed with specific sphingolipidoses (n = 119), and pathological controls (patients suffering from LSDs other than sphingolipidoses (n = 21)) were used. The available demographic details, such as the treatment status, sex, age, and mutation of participants, are summarized in Tables S1 and S2.

Briefly, the patients recruited for this study according to each disease were as follows: Fabry disease, 97 patients (untreated males (UFMs): n = 19; treated males (TFMs): n = 22); untreated females (UFFs): n = 33; treated females (TFFs): n = 23); Gaucher disease, 14 patients (untreated (UG): n = 9; treated (TG): n = 5); GM1 gangliosidosis: two treated patients; metachromatic leukodystrophy (MLD), four untreated patients; and Niemann–Pick disease type C, one treated patient. Healthy control samples were obtained from healthy participants with no incidental findings (<18 years: n = 26; ≥18 years: n = 34). Samples from patients suffering from other LSDs were used as pathological controls: MPS type I (n = 4); MPS type II (n = 5), MPS type III (n = 2), MPS type IV (n = 6), MPS type VI (n = 2), MPS type VII (n = 1), and Pompe disease (n = 2).

Liquid urine specimens were vortexed for 30 s, and one mL was deposited on a 5 cm Whatman 903 filter paper disk (CF10, GE Healthcare, Chicago, IL, USA). The disks were flat-dried for 4 h at room temperature, then transferred at −30 °C until the UPLC-MS/MS analysis. For the MLD patients, no liquid urine specimens were available and only a quarter of a 5 cm DUS was available per patient for the extraction.

### 2.3. Reagents

LC/MS-grade water (H_2_O), methanol (MeOH), American Chemical Society-grade ammonium formate, o-phosphoric acid (H_3_PO_4_, 85%), and ammonium hydroxide (NH_4_OH, 28–30% purity) were purchased from Fisher Scientific (Hampton, NH, USA). Formic acid (FA, 99+%) was obtained from Thermo Fisher Scientific (Waltham, MA, USA) and LC-MS-grade acetonitrile, ReagentPlus-grade dimethyl sulfoxide (DMSO) (≥99.5%), ammonium formate (Amm. Form.) (≥99.995% trace metals basis) and creatinine (≥98%) were obtained from Millipore Sigma (Burlington, MA, USA). Glucosylsphingosine (d18:1) (psychosine; GluSph), galactosylsphingosine (d18:1) (GalSph), globotriaosylsphingosine (d18:1) (lyso-Gb_3_), lyso-sulfatide (d18:1) (lyso-Sulf), lyso-monosialoganglioside GM1 (d18:1) (lyso-GM1), lyso-monosialoganglioside GM2 (d18:1) (lyso-GM2), ^13^C_6_-glucosylsphingosine (d18:1) (^13^C_6_-GluSph), D-erythro sphingosylphosphorylcholine (d18:1) (D-erythro-lysosphingomyelin; lyso-SM) and N-glycinated lyso-sulfatide (d18:1) (N-Gly-lyso-Sulf) were purchased from Cayman Chemical Company (Ann Arbor, MI, USA). D_9_-sphingosylphosphorylcholine (d18:1) (d_9_-lyso-SM) was purchased from Avanti Polar Lipids (Alabaster, AL, USA). The d_3_-creatinine standard (99.8 atom %D) was purchased from CDN Isotopes (Pointe-Claire, QC, Canada). N-glycinated globotriaosylsphingosine (d18:1) (N-Gly-lyso-Gb_3_) was synthesized and purified in our laboratory [51]

### 2.4. Calibration Standards and Quality-Control Preparation

Spiked (S-) and urine (U-) in-house quality controls (QCs) (lower limit of quantification level (LLOQC), low level (LQC) at 3xLLOQC, medium level (MQC) at the midrange of the calibrators and high level (HQC) at the higher range of the calibrators) were used to evaluate the effectiveness and robustness of the method, as described in the Section 2.7 Method Validation. The S-QCs and calibrators were prepared on the day of the analysis, while the U-QCs were prepared before the analysis and stored with the working solutions and urine matrices in the freezer (−30 °C) until analysis. The concentration of the QCs and calibrator working solutions are shown in Table S3.

For creatinine, the S-QCs were freshly prepared before the extraction by spiking 100 μL of working solutions, prepared in water, uniformly on a 5 cm blank filter paper disk, which was flat-dried at room temperature (21.5 °C) for 30 min. Blank filter paper disks (without urine) were used because the urine matrix is not exempt from endogenous creatinine. The U-QCs were made from a pool of urine samples from five healthy controls (three males and two females) to an endogenous creatinine concentration at 15 mmol/L, then diluted with water to obtain the same final concentration ranges as the S-QCs, before depositing 1 mL on a 5 cm blank filter paper disk flat-dried at room temperature (21.5 °C) for at least 4 h. For the creatinine calibrators, eight working solutions prepared in water were spiked as the creatinine S-QCs.

For lysosphingolipids, the S-QCs were prepared similarly to the S-QCs for creatinine, but the working solutions were prepared in 80:20 MeOH:DMSO, sonicated 5 min prior to use and spiked on 5 cm filter paper disks containing 1 mL of urine with an endogenous creatinine level at 7 mmol/L, already dried. The U-QCs were made by spiking the same liquid urine pool used to prepare the creatinine U-MQCs with the same multiplex lysosphingolipid working solutions used for the S-QCs before depositing 1 mL on a 5 cm blank filter paper, as mentioned previously for the creatinine U-QCs. For the lysosphingolipid calibrators, eight working solutions prepared in 80:20 MeOH:DMSO were spiked as the lysosphingolipids S-QCs.

### 2.5. Extraction of Urine from Filter Paper Disks

Prior to the extraction, the samples, QCs, and calibrators were thawed at room temperature (21.5 °C). A volume of 100 µL of multiplex lysosphingolipid internal standard working solution, containing 50 nmol/L of N-Gly-lyso-Sulf, ^13^C_6_-GluSph, N-Gly-lyso-Gb_3_ and d_9_-lyso-SM in 80:20 MeOH:DMSO, and 100 µL of creatinine internal standard working solution, containing 10 mmol/L of d_3_-creatinine in water, was deposited on each filter paper and flat-dried at room temperature for 30 min. Afterwards, each 5 cm filter paper disk was folded in half and deposited in a 20 mL glass vial with the fold on top. To elute the compounds, 3 mL of 80:20 MeOH:H_2_O was added to the glass vial, covered with parafilm M PM-996 (Bemis Co., Neenah, WI, USA), and agitated for 30 min at 300 rpm on a model G2 gyratory shaker (New Brunswick Scientific Co., Edison, NJ, USA). Then, 1 mL of eluate was transferred to a 13 × 100 mm borosilicate test tube containing 100 µL of H_3_PO_4_ 2% in water. For each acidified eluate, the preparations were loaded on mixed-mode strong cation-exchange cartridges (Oasis MCX, 1 cc, 30 mg, Waters Corp., Milford, MA, USA) previously conditioned with 1200 µL of MeOH followed by 1200 µL of H_3_PO_4_ 2% in water. Each cartridge was washed successively with 1200 µL of water containing 2% FA and 1200 µL of 50:50 MeOH:H_2_O containing 0.2% FA, then transferred in a clean 13 × 100 mm borosilicate test tube to eluate the compounds by gravity with 1200 µL of 2% NH_4_OH in MeOH. The collected eluates were dried under a nitrogen flow for about 60 min. The prepared samples were resuspended in 100 µL of 60:40 mobile phase A:B (82.2:17.8 ACN:H_2_O + 5mM Amm. Form. + 0.3% FA). The reconstituted samples were transferred to a 300 µL borosilicate insert fitted in a 2 mL borosilicate capped vial to be injected into the UPLC-MS/MS system.

### 2.6. UPLC-MS/MS Analysis of Lysosphingolipids and Creatinine

An Acquity UPLC I-Class Plus combined with a triple quadrupole Xevo TQ-XS (MS/MS) from Waters Corporation (Milford, MA, USA) was used to analyze the targeted molecules. An HILIC column approach with a Halo HILIC column (90 Å, 2.1 × 50 mm, 1.6 µm, Advanced Materials Technology, Wilmington DE, USA) combined with an Acquity Column In-Line filter (2.1 mm nut, Frit 0.2 µm, Waters Corp., Milford, MA, USA) was used to separate the lysosphingolipids according to the interaction of their polar moiety with the UPLC column. This chromatographic approach was successfully used in our laboratory in previous studies on sphingolipids [53]. The UPLC-MS/MS parameters are described in Table 1. Briefly, the system was operated in the selected reaction monitoring (SRM) mode with positive electrospray ionization (ESI+). Using this tandem mode, one precursor ion specific to each studied lysosphingolipid was selected in the first quadrupole, and fragmented in the collision cell, followed by the selection of a specific and sensitive fragment ion in a second quadrupole. The SRM transitions are described in Table 2. The M + 1 peak was used for the detection of creatinine and its internal standard to avoid saturating the MS detector signal.

### 2.7. Method Validation

The method validation was performed for research purposes according to the guideline recommendations from the FDA guidance for industry [64]. All the S-QCs levels were used to evaluate the assays from three intradays (n = 5) and one interday (n = 3) for accuracy (%bias) and precision (%CV). The U-LQCs and U-HQCs were used to evaluate the sample stability (n = 3) up to 4 months (117 days) for lysosphingolipids and 1 month (26 days) for creatinine at different temperatures: high temperature (35 °C), room temperature (21.5 °C), 4 °C, −30 °C, and –80 °C. The effects of 3 and 5 freeze–thaw cycles (n = 3) were also assessed for all the biomarkers with the U-LQCs and U-HQCs. The autosampler stability (10 °C) of the extracted U-LQCs and U-HQCs was evaluated for 24 h (n = 3). Biomarkers were considered stable in samples when the accuracy (bias nominal%) was ≤15% at each concentration level within the precision range of the replicates. 

The adsorption of the metabolites to polypropylene tubes and glass tubes was not investigated because no significant impact was observed in our previous study using liquid urine samples [53]. The limit of detection (LOD) and the lower limit of quantification (LLOQ) were, respectively, established as 3 and 10 times the standard deviation of the analyte concentration after 10 injections of S-LLOQ.

The criteria for the extraction recovery and matrix effect (nominal bias%) were assessed based on Matuszewski et al.’s approach [65]. Three urine matrices with different levels of endogenous creatinine (1.5, 7, and 15 mM) were used to represent the impact of the matrix on the results in different spiked QC ranges (L, M, and H). Pre- and post-spiked extracted matrices were used to evaluate the compound recovery, while post-spiked extracted matrices with spiked blank results were compared to evaluate the ion suppression or enhancement. Internal standards were added in the final step before injection on the instrument. The detailed approach for these assays is described in Protocol S-1.

The sample dilution impact was investigated by comparing the measured concentrations of sphingolipids normalized to creatinine in the U-MQCs of different filter paper sizes (n = 5): 5 cm filter paper disk, half of a 5 cm filter paper disk and a quarter of a 5 cm filter paper disk. The carry-over impact was evaluated by analyzing a blank sample after the injection of the most concentrated calibrator, HQCs, and every 20 injections. The selectivity was evaluated by analyzing a blank sample, zero calibrators, and six healthy controls without internal standards to verify if they were free of interferences at each SRM transition and the chromatographic retention times of the biomarkers and the internal standards. Finally, the LOD, LLOQ, and specificity were the only parameters validated for the analogs of GluSph and lyso-Gb_3_, considering that commercial standards are not available for these molecules. These tests were performed with diluted samples obtained from an untreated Fabry male and untreated Gaucher patient with a confirmed diagnosis excreting related analogs.

### 2.8. Statistical Analyses

The MS data generated were processed with MassLynx-TargetLynx V4.2 SCN 1040 software (Waters, Milford, MA, USA). The calibration curve parameters for all the compounds were defined as a linear curve with the point of origin excluded and with a 1/x weighting. Statistical analysis was performed using GraphPad Prism 10.2.2. software (Dotmatics, Boston, MA, USA). Non-parametric tests were chosen because of the small group size and the non-normal distribution of the results analyzed. Differences between the healthy controls, pathological controls, other sphingolipidoses, and specific patient groups were determined by comparing the group medians with the Kruskal–Wallis test with the post hoc Dunn’s multiple comparisons test. For the healthy control group, the Spearman test was used to evaluate if there was a correlation between the biomarker levels and the participant age, and the Mann–Whitney U test was used to evaluate if the biomarker levels were associated with the sex of the participant. The area under the receiver operating characteristic (ROC) curve (AUC) was calculated to evaluate the overall diagnostic accuracy of the putative biomarkers and related analogs in the Fabry and Gaucher disease untreated groups compared to the healthy controls (n = 59). The ROC curve represents the true-positive rate (sensitivity: (true positives/(true positives + false negatives)) on the *y*-axis versus the false-positive rate (1 − (specificity: true negatives/(true negatives + false positives)) on the *x*-axis, as a function of the cut-off value tested. The optimal cut-off according to the Youden index (sensitivity + specificity − 1) was evaluated. The diagnostic reliability of the assay regarding the other sphingolipidoses could not be evaluated due to the limited number of patients. However, the normal reference values were determined by calculating the 95th percentile of the results from the biomarker levels of the healthy control specimens.

## 3. Results and Discussion

### 3.1. Chromatographic Separation of Lyso-Sulf, GluSph and GalSph

**Figure 1:**
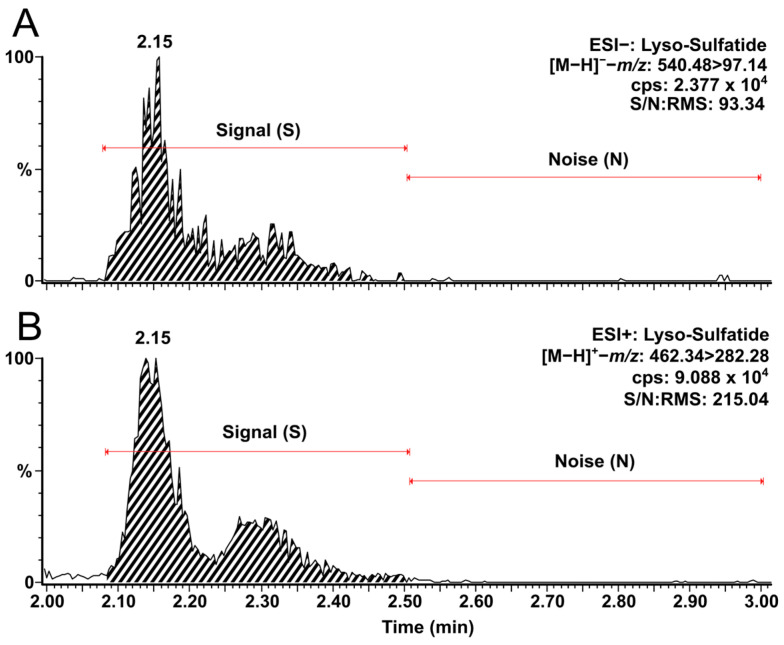
Chromatograms of lyso-Sulf using: (**A**) negative electrospray ionization (ESI−); and (**B**) positive electrospray ionization (ESI+) at 1 nmol/L. Red lines show chromatogram sections to calculate the signal-to-noise ratio (S/N) and the root mean square (RMS) of lyso-Sulf with the MassLynx software. Cps: counts per second.

In our previous studies, it was already shown that chromatographic HILIC approaches were efficient in separating structural isomers, such as GluSph and GalSph, which allowed the differentiation between Gaucher disease and Krabbe disease biomarkers and ensured a reliable quantification [53]. It was observed that with positive electrospray ionization (ESI+), the lyso-Sulf precursor ion lost its H_2_SO_4_ group by in-source fragmentation and had the same mass-to-charge (*m*/*z*) ratio as GluSph and GalSph (*m*/*z* 462.34). Even if another study selected the *m*/*z* 542 precursor ion in ESI+ for the quantification of lyso-Sulf in plasma [66], we observed, by MS infusion (50:50 MeOH:H_2_O + 10 mM ammonium formate), that the in-source fragmentation ion was ten times more sensitive than the *m*/*z* 542 precursor ion in our specific setting. Also, lyso-Sulf was analyzed in negative electrospray ionization (ESI−) in our previous study [53]. However, we used a more recent triple quadrupole MS for this study, and with our optimized parameters, at molar equivalence, the precursor ion at *m*/*z* 462.34 was three times more abundant in ESI+ than analyzing *m*/*z* 540.48 using ESI− according to the signal-to-noise ratio (S/N) (Figure 1). For these reasons, the lyso-Sulf precursor ion at *m*/*z* 462.34 was selected.

**Figure 2:**
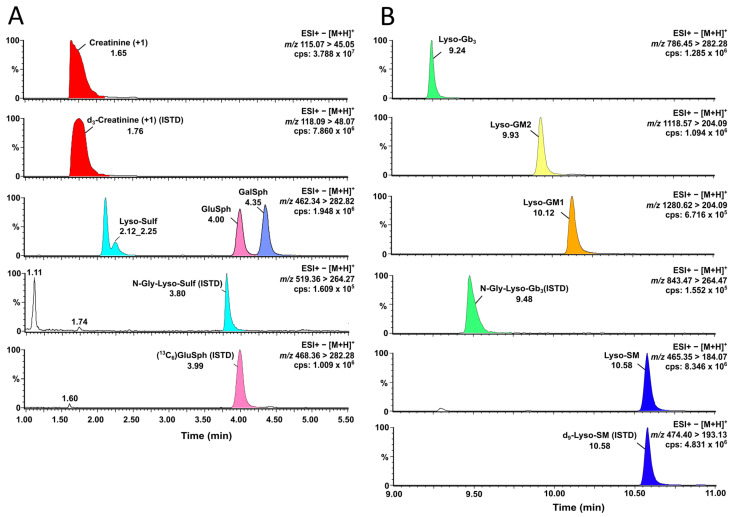
Chromatograms of the principal biomarkers analyzed by UPLC-MS/MS. (**A**) Extracted ion currents from SRM transitions of creatinine, lyso-Sulf, GluSph, GalSph and their internal standards; and (**B**) extracted ion currents from SRM transitions of lyso-Gb_3_, lyso-GM2, lyso-GM1, lyso-SM and their internal standards. Cps: counts per second; %: relative abundance; ESI+: positive electrospray ionization; *m*/*z*: mass-to-charge ratio; ISTD: internal standard.

**Figure 3:**
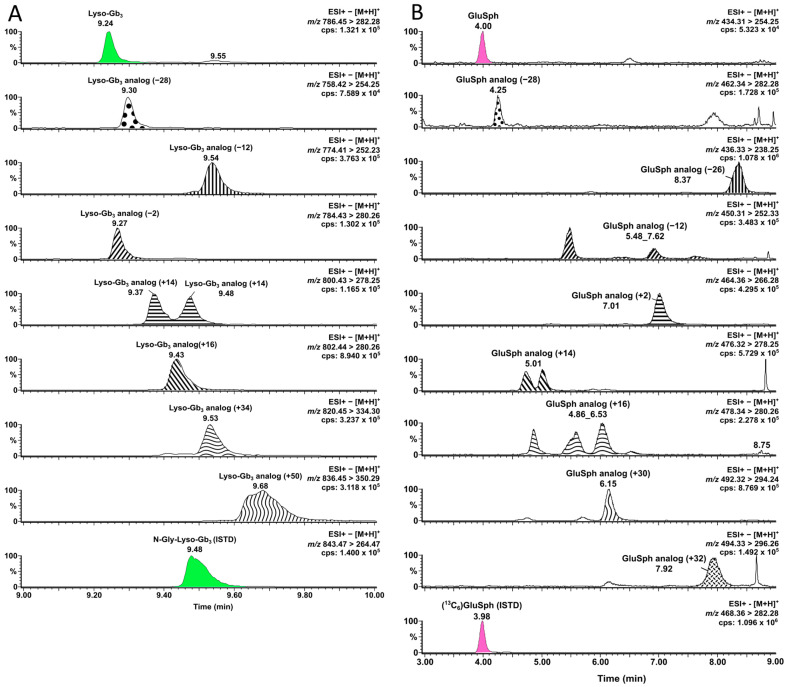
Chromatograms of separated (**A**) Lyso-Gb_3_ and its analogs (−28, −12, −2, +14, +16, +34, and +50) using UPLC-MS/MS for Fabry disease; and (**B**) GluSph and its analogs (−28, −26, −12, +2, +14, +16, +30, and +32) by UPLC-MS/MS for Gaucher disease. Cps: counts per second; %: relative abundance; ESI+: positive electrospray ionization; *m*/*z*: mass-to-charge ratio.

Considering that lyso-Sulf was not retained with the previous chromatography strategy developed [53], a new chromatographic method was devised to optimize the lyso-Sulf retention time while still allowing a good separation of GluSph, and GalSph. The HPLC column previously used (Halo^®^ HILIC 2.7 (4.6 × 150 mm, 2.7 µm, Advanced Materials Technology, Wilmington, DE, USA) was replaced by the equivalent particles of a UPLC column to improve the chromatographic resolution and separation efficiency (Halo^®^ HILIC 90 Å (2.1 × 50 mm, 1.6 µm, Advanced Materials Technology, DE, USA), with a slight modification of the mobile phase A to ensure the retention of lyso-Sulf. Also, the strong wash and the injection mode were modified to avoid carry-over in-between injections using the different UPLC system. All the other compounds targeted in this study were still clearly separated by the new UPLC-MS/MS method developed, as shown in Figure 2. Furthermore, analogs of lyso-Gb_3_ and GluSph were analyzed, and their chromatographic separation is shown in Figure 3. The urinary analogs of lyso-Gb_3_ (−28, −12, −2, +14, +16, +34 and +50) in Fabry disease patients and of GluSph (−28, −26, −12, +2, +14, +16, +30, and +32) in Gaucher disease patients were discovered in previous metabolomic studies in urine [5,7]. For Fabry disease, analogs +16, +34, and +50 had a positive association with the left ventricular mass index in Fabry patients from Taipei carrying the IVS4 + 919G>A mutation (cardiac variant) [40]. For Gaucher disease, larger cohorts are needed to understand the clinical significance of GluSph-related analogs in urine [52].

### 3.2. Method Validation Results

**Figure 4:**
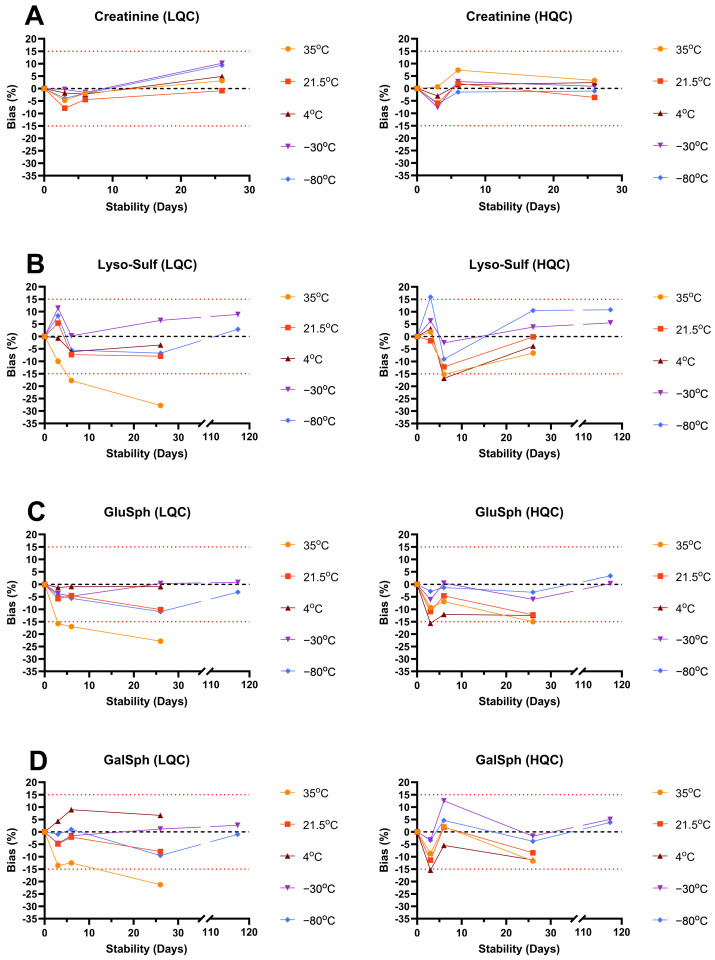
Long-term stability follow-up for (**A**) creatinine; (**B**) lyso-Sulf; (**C**) GluSph; and (**D**) GalSph at several temperatures and concentration levels. LQC: low-concentration quality control; HQC: high-concentration quality control; upper red dot line: maximum limit of 15% of bias compared to the nominal concentration; lower red dot line: lower limit of −15% of the bias compared to the nominal concentration.

**Figure 5:**
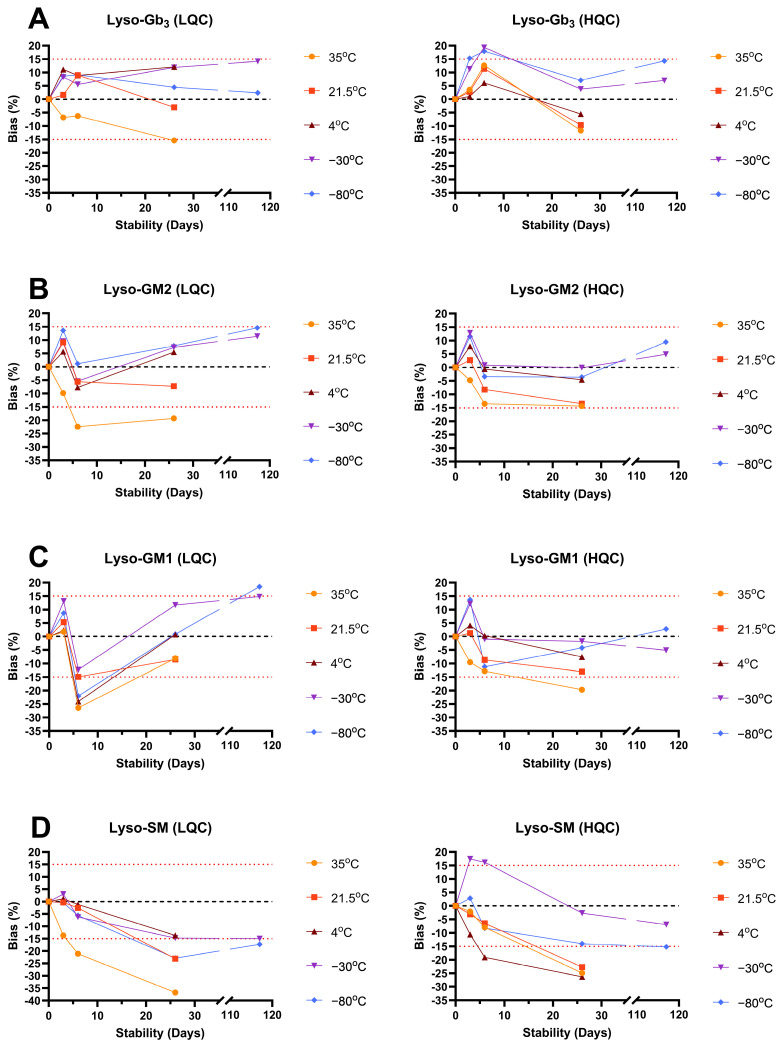
Long-term stability follow-up for (**A**) lyso-Gb_3_, (**B**) lyso-GM2, (**C**) lyso-GM1 and (**D**) lyso-SM at several temperatures and concentration levels. LQC: low-concentration quality control; HQC: high-concentration quality control; upper red dot line: maximum limit of 15% of bias compared to the nominal concentration; lower red dot line: lower limit of −15% of bias compared to the nominal concentration.

The intra- and interday accuracy, and the intra- and interday precision assays, are summarized in Table S4, while the LOD, LLOQ and linearity assays for sphingolipids and creatinine are shown in Table S5. The matrix effect and extraction recovery assays are shown in Table S6. The stability results are shown in Table S7, and in Figure 4 and Figure 5, while the freeze–thaw assay results are shown in Table S8. The sample dilution impact assays are shown in Table S9.

Briefly, no carry-over and no cross-interference between the targeted compounds and internal standards were observed during the validation and the sample analysis. Except for lyso-SM (bias%: 23.6% to 57.2%, but CVs% < 15%), the three intraday assays (3 × n = 5) revealed that the CVs% and biases% were <20% for the LLOQC and <15% for the LQC, MQC, and HQC. Nevertheless, the lyso-SM interday assays showed precision results for all the levels of QCs with CV% < 15%, except for LLOQC range (CV%: 22.0%). Considering this, the lyso-SM measurements were considered semi-quantitative for this method. The LOD and LOQ were established. Lyso-GM2 and lyso-GM1 had higher limit values compared to the other biomarkers and their calibration curves were adjusted according to this result. The Pearson’s correlation coefficients (r), and the coefficients of determination (R^2^) were ≥0.995 (n = 15) for the calibration curve in the validation of all the compounds.

The extraction recovery and matrix effect were investigated for the targeted sphingolipids and creatinine. Ideally, the internal standards selected for quantification should have similar physical and chemical properties and ionization efficiency compared to the analytes. Usually, stable isotope-labelled (SIL) standards are used [67]. SIL standards were commercially available for creatinine (d_3_-creatinine), lyso-SM (d_9_-lyso-SM), and GluSph (^13^C_6_-GluSph) during the study design. The N-Gly-lyso-Gb_3_ internal standard was used for lyso-Gb_3_ and its analogs, lyso-GM1, and lyso-GM2 due to their retention time proximity. The GalSph and GluSph analogs were corrected with ^13^C_6_-GluSph, and N-Gly-lyso-Sulf was used to correct lyso-Sulf due to their structural similarities. Due to the limitation of the availability of SIL standards, the methodology was developed to limit the matrix effect and to optimize the extraction recovery. The results obtained showed that the creatinine levels do not affect the extraction recovery, and the matrix effect seems limited. The creatinine matrix spiked with high concentrations of creatinine was excluded because the MS signal of creatinine saturated the instrument detector, and the results were not reliable. However, other levels have shown similar and acceptable results for creatinine. The extraction recovery was ≥63.5% and the matrix effect varied between −12.8% and 17.0% for all the biomarkers except lyso-Gb_3_, lyso-GM2, and lyso-GM1, which have higher enhancement effects, especially when the urine is more concentrated (matrix effect bias min-max range: 10.2% to 52.5%). However, within the same biomarker analysis, the matrix effect and recovery were similar independently of the creatinine and analyte levels.

The biomarker stability was assessed at 35 °C, 21.5 °C, 4 °C, −30 °C and −80 °C until 117 days for lysosphingolipids and 26 days for creatinine. These conditions were used to simulate different environmental impacts on the samples. Creatinine was stable for at least 26 days at all the concentrations and temperatures (bias between −7.6% to 10.2%). For lysosphingolipids, all the compounds were stable for at least 6 days at 35 °C but showed important degradation (>20% bias) at 26 days (nominal bias between −19.0% to −36.8%). At 21.5 °C and 4 °C, all the lysosphingolipids, except lyso-SM, were stable at least for 26 days. Lyso-SM showed higher bias (−13.7% to −26.3%) but good stability for at least 6 days. At −30 °C and −80 °C, only lysosphingolipids were evaluated over 26 days and were stable for at least 117 days. The autosampler (10 °C) stability showed nominal bias ≤7.3% for all the compounds.

The results have shown that the compounds are stable after three freeze–thaw cycles from −30 °C to 21.5 °C. Only lyso-GM1 had a bias over 20% for three and five freeze–thaw cycles, which might be caused by the large CV% for this molecule in these assays.

Finally, the results have shown that dilution factors of two and four were reliable because all the nominal biases were below 15.1%, with coefficients of variability below 9.2% between replicates. However, the MS signal of the compounds was reduced, and the results should respect the LOD and LLOQ established.

### 3.3. Normal Reference Values

To evaluate the normal reference values, 59 urine samples from healthy controls (24 males (<18 yrs: n = 11, ≥18 yrs: n = 13); 35 females (<18 yrs: n = 15, ≥18 yrs: n = 20)) were analyzed to establish the lysosphingolipid reference values normalized to creatinine according to the 95th percentile. Except for lyso-SM and GluSph, no lysosphingolipids were detected in the healthy controls. For the lyso-SM levels, no relation or significant differences were observed according to age and sex (*p*-value > 0.05). For GluSph, the levels were significantly different between male and female controls according to the Mann–Whitney U test (*p*-value < 0.0001). However, due to the limited Gaucher patient samples available to evaluate the normal reference values, the 95th percentile was established with the total cohort for the GluSph. The same strategy was used for all the lysosphingolipids targeted. For analogs related to lyso-Gb_3_ and GluSph, the reference values were established individually and as a group. The normal reference values are summarized in Table S10. Briefly, only the GluSph (37 pmol/mmol creatinine) and lyso-SM (293 pmol/mmol) levels were quantified in the healthy controls.

### 3.4. Lysosphingolipid Levels in Patients and Controls

Urine samples dried on filter paper from patients diagnosed with specific sphingolipidoses (n = 119) were analyzed, and the results normalized to creatinine (pmol/mmol creatinine) are summarized in Table S11. The lyso-Sulf, GluSph, GalSph, lyso-Gb_3_, lyso-GM1, lyso-GM2, and lyso-SM levels were measured in all the samples. Relative quantifications of analogs of Lyso-Gb_3_ and GluSph were performed with their putative biomarker curve.

The number of patient samples available was limited for some sphingolipidoses (MLD, Krabbe, GM1, GM2, and Niemann–Pick C) due to the low prevalence of these diseases. Although it is feasible to quantify lyso-Sulf, GalSph, lyso-GM1, lyso-GM2, and lyso-SM in DUS, further validation in larger patient cohorts is required to better evaluate the sensitivity and specificity before the clinical implementation of these biomarkers in urine. However, a slight amount of lyso-SM was detected in specimens obtained from all the patients with sphingolipidoses, pathological and healthy controls, and there was no significant difference observed between the Niemann–Pick type C patient sample (53 pmol/mmol creatinine) compared to the other sphingolipidoses. Niemann–Pick type C is a less severe disease form compared to types A and B and should not affect the enzyme activity of acid sphingomyelinase [17]. A previous study showed increased plasma levels of lyso-SM in Niemann–Pick types A and B. Lyso-SM 509, a recently detected biomarker for Niemann–Pick, was increased in Niemann–Pick type C in the same study [42]. The lyso-SM level was also increased in dried blood spots of Niemann–Pick B. No study evaluating the detection of Niemann–Pick A/B using urine specimens was found [68]. Furthermore, six female Fabry patients, one male Fabry patient, four MLD patients, four MPS patients and three female healthy controls showed levels over 293 pmol/creatinine. Consequently, more samples are needed to confirm the reference value proposed in this study and to evaluate the potential of lyso-SM for the monitoring and follow-up of Niemann–Pick patients using urine dried on filter paper. Lyso-GM1 and lyso-GM2 were not detected in any groups. These biomarkers were, respectively, detected in plasma samples of GM1 patients and GM2 patients in a previous study [69]. In our current study, samples from only two GM1 treated patients were available, and no large study was found analyzing lyso-GM1 and lyso-GM2 in GM1 and GM2 patient urine samples. For lyso-sulfatide, only quarters of a 5 cm filter paper disk from diagnosed MLD patients with a concentration of creatinine near the LLOQ were available. Only one MLD patient (unknown form) had elevated lyso-Sulf, while no lyso-Sulf was detected in the three other patients.

**Figure 6:**
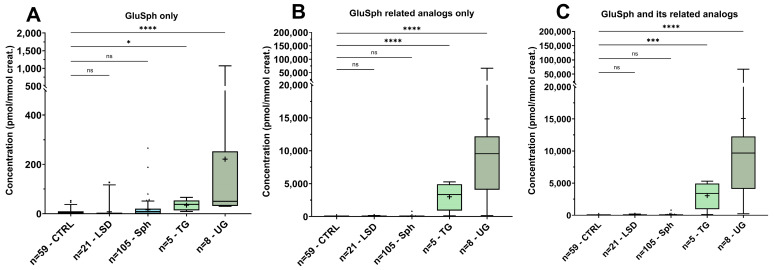
Box plots of the urinary levels of GluSph and the eight related analogs normalized to creatinine (pmol/mmol creatinine) in the healthy control group (CTRL, n = 59), pathological control group (LSD, n = 21), other sphingolipidoses group (Sph, n = 105), treated Gaucher patient group (TG, n = 5) and untreated Gaucher patient group (UG, n = 8) from urinary samples dried on filter paper; box plots represent the normalized urinary levels of (**A**) GluSph levels only; (**B**) GluSph related analog levels only; and (**C**) total levels of GluSph and its related analogs. The lower and upper limits shown by the box plots are the 25th and 75th percentiles, respectively. The center horizontal box line is the median. The symbol “+” is the mean. The whiskers correspond to the highest and lowest values at the 95th and 5th percentile, respectively. Values outside the 95th percentile are considered as outliers. Comparison of the CTRL group with other groups with the Kruskal–Wallis post hoc Dunn’s test: ns: non-significant, *: *p*-value ≤ 0.05; ***: *p*-value ≤ 0.001; ****: *p*-value ≤ 0.0001.

**Figure 7:**
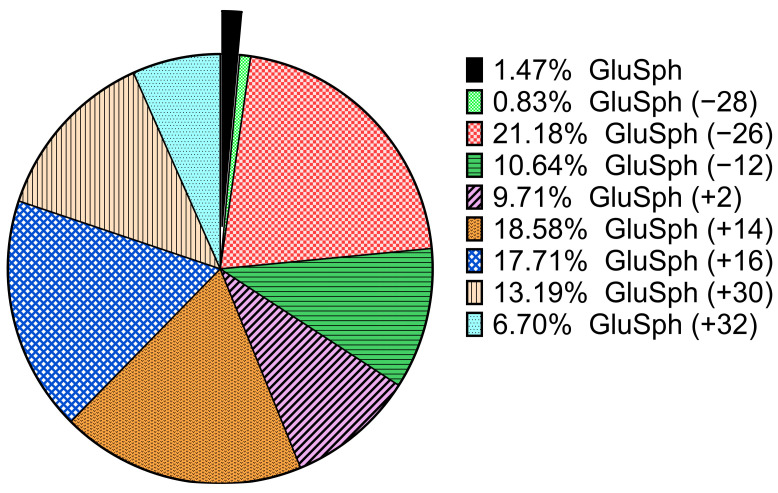
Mean distribution of GluSph and its eight related analogs (−28, −26, −12, +2, +14, +16, +30, +32) in the untreated Gaucher disease group (n = 5).

According to the Kruskal–Wallis test (*p*-value < 0.0001), there were significant differences between the median levels of GluSph and its related analogs between the Gaucher patients, other patients and controls. The post hoc Dunn test analysis of the Kruskal–Wallis test revealed significant differences between groups. The results are shown in Figure 6 and in Table S12. The GluSph levels were significantly different in the Gaucher patients compared to the other groups. As expected, marked elevations of GluSph and its related analogs were found in the untreated patients compared to the treated patients. However, the differentiation was less important when the statistical analysis was performed with only the GluSph values. Interestingly, some controls and other sphingolipidoses had a slight elevation of GluSph, which may impact the median rank in the Dunn’s test comparison between groups. Nevertheless, some related analogs were detected in other sphingolipidoses than Gaucher disease but were still negligible compared to levels from the Gaucher patients. The distribution of analogs −26 (21.18%), −12 (10.64%), +2 (9.71%), +14 (18.58%), +16 (17.71%), +30 (13.19%), +32 (6.70%) showed that the analog levels tend to be largely higher than GluSph (1.47%) itself (Figure 7).

**Figure 8:**
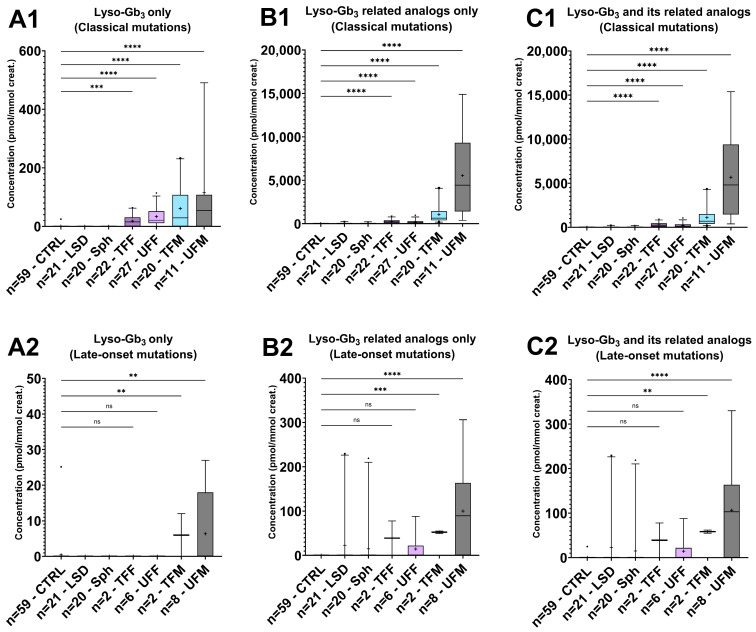
Box plots of the urinary levels of lyso-Gb_3_ and its eight related analogs normalized to creatinine (pmol/mmol creatinine) in the healthy control group (CTRL, n = 59), pathological control group (LSD, n = 21), other sphingolipidose groups (Sph, n = 20), treated Fabry female patient group (TFF, n = 22 with classical mutations (CM) and n = 2 with late-onset mutations (LO)), untreated Fabry female group (UFF, CM: n = 27, LO: n = 6), treated Fabry male group (TFM: CM: n = 20, LO: n = 2), and untreated Fabry male group (UFM: CM: n = 11, LO: n = 8) from urinary samples dried on filter paper; box plots represent normalized urinary levels of (**A1,A2**) lyso-Gb_3_ levels only in controls and patients with (1) CM and (2) LO; (**B1,B2**) lyso-Gb_3_ related analog levels only in controls compared to patients with (1) CM and (2) LO; and (**C1,C2**) total levels of lyso-Gb_3_ and its related analogs in controls compared to patients with (1) CM and (2) LO. The lower and upper limits shown by the box plots are the 25th and 75th percentiles, respectively. The center horizontal box line is the median. The symbol “+” is the mean. The whiskers correspond to the highest and lowest values at 95th and 5th percentile, respectively. Values outside the 95th percentile are considered as outliers. Comparison of the CTRL group with other groups with Kruskal–Wallis post hoc Dunn’s test: ns: non-significant, **: *p*-value ≤ 0.01; ***: *p*-value ≤ 0.001; ****: *p*-value ≤ 0.0001.

**Figure 9:**
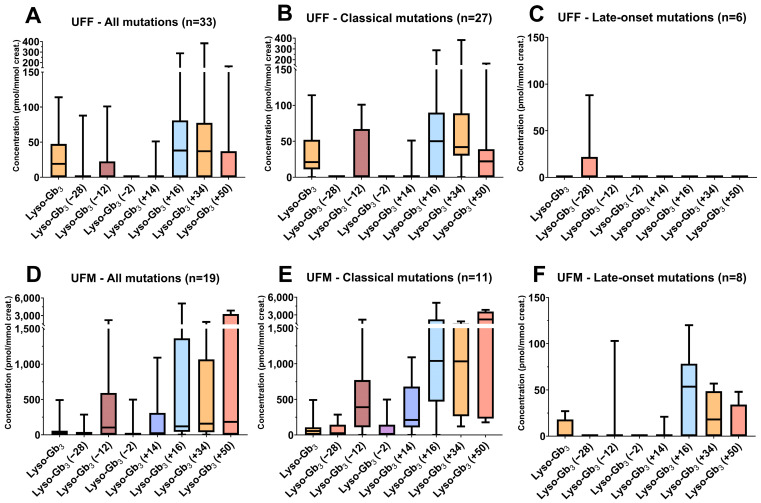
Box plots of the urinary levels of lyso-Gb_3_ and its eight related analogs (−28, −12, −2, +14, +16, +34, +50) normalized to creatinine (pmol/mmol creatinine) in the untreated Fabry female (UFF) and untreated Fabry male (UFM) groups according to the classical and nonclassical (late-onset phenotype) mutations. (**A**) Results regrouping all the UFF; (**B**) results regrouping only UFF with classical mutations; (**C**) results regrouping only UFF with nonclassical mutations; (**D**) results regrouping all the UFM; (**E**) results regrouping only UFM with classical mutations; and (**F**) results regrouping only UFM with nonclassical mutations. The lower and upper limits shown by the box plots are the 25th and 75th percentiles, respectively. The center horizontal box line is the median. The whiskers correspond to the highest and lowest values.

For lyso-Gb_3_ and its related analogs, the Dunn’s test results (comparison of the CTRL group to other groups for these biomarkers) and biomarker levels are shown in Figure 8 and summarized in Table S13. The Fabry patients were separated according to their sex and their predicted likely phenotype according to their mutation. Lyso-Gb_3_ and its related analogs were significantly different in the Fabry disease patients compared to the pathological and healthy controls, especially for patients with classical mutations. Fabry patients with classical mutations have higher levels of lyso-Gb_3_ and analog levels than patients with late-onset mutations. It was previously shown that patients with classical mutations are more likely to present an elevation of lyso-Gb_3_ and related analogs compared to patients with non-classical mutations (late-onset phenotypes) [40,41,70]. Considering that only five late-onset mutations from Fabry patients were evaluated in the current study, and that these were not equally distributed in each study group, this might have affected the biomarker profiles detected. All the patients carrying the N215S mutation (5/8 UFM; 1/6 UFF; 2/2 TFM), a cardiac variant mutation, had higher analog levels compared to the patients with other late-onset mutations. This was previously observed in another cohort of Fabry patients [41,70]. As also observed in the current study, patients with intronic mutations such as c.639+919G>A (1/2 TFF; 1/6 UFF; 2/8 UFM) or c.640-801G>A (2/6 UFF; 1/8 UFM) had no urinary lyso-Gb_3_ or related analogs increased. The male Fabry group tends to have higher levels of biomarkers compared to the female Fabry patient group, even if they are treated. The variation of the biomarker levels in the patient groups may partially be explained by the marked heterogeneity in the *GLA* mutations and the sex of the patients [71]. Unfortunately, the α-Gal enzyme activities for all the Fabry patients were not available and cannot be taken into consideration in our interpretation. Moreover, the X-linked inheritance of the disease, especially in heterozygous females, may lead to phenotypic variations according to the random inactivation of one of the X chromosomes in somatic cells [72]. The distributions of lyso-Gb_3_ and its analogs in the untreated patients regrouped according to their sex and mutation type (classical or non-classical) are shown in Figure 9. The lyso-Gb_3_-related analogs +16, +34, and +50 were the most abundant analogs in the male and female Fabry patients and were sometimes higher than lyso-Gb_3_ itself. They could also be quantified in patients with late-onset mutations, even if their excretion levels were reduced, especially in the UFM group [40,70]. Considering that there are over 1000 *GLA* gene variants, including pathogenic mutations, variants of unknown significance and benign mutations [21], larger cohort studies are needed to evaluate the analog profile in the urine samples of patients with other late-onset variants.

### 3.5. Receiver Operating Characteristics (ROC Curve)

The AUCs were used to assess the efficacy of lyso-Gb_3_, GluSph and their related analogs in discriminating untreated patients from healthy controls without considering their genotype. The sensitivity and specificity were evaluated at the optimal cut-off value suggested by the highest Youden index value. The AUCs, optimal cut-off levels selected, sensitivity and selectivity results are summarized in Table 3. The Youden index values are summarized in Table S14. The AUCs results were statistically significant in the analyzed subgroups (*p*-value < 0.0001), and over 0.75, suggesting that these urinary biomarkers are clinically useful for diagnostic reliability. For the untreated Fabry patients, the AUC results seem to be more favourable if the complete profile of lyso-Gb_3_ and its related analogs was used to discriminate patients from controls (UFF: AUC = 0.877; UFM: AUC = 0.920) compared to using lyso-Gb_3_ only (UFF: AUC = 0.811; UFM: AUC = 0.758). Similarly, for the untreated Gaucher patients, the AUCs results tend to be slightly favourable when using the complete profile of GluSph and its related analogs (UG: AUC = 1.000) compared to using GluSph only (UG: AUC = 0.966). Interestingly, the AUC value appears to improve in the UFM group when the analogs are included in the cut-off evaluation compared to the analysis of lyso-Gb_3_ itself. According to Table S1, 11/19 UFM patients have a classical mutation and 8/19 UFM patients have a late-onset mutation. However, three patients with classical mutations and six patients with late-onset mutations have an elevation of lyso-Gb_3_-related analogs, but not of lyso-Gb_3_. This observation was also made in the UFF (3/33 patients) cohorts, but not in the UG group. Even if larger cohorts are needed to optimize the cut-off values for high-risk screening and to confirm these results due to the limited number of variants studied, these data suggest that analyzing a complete profile of lysosphingolipids in urine is a more comprehensive approach than analyzing the lyso-Gb_3_ levels alone. For the UG patients, the analogs might be interesting in patients with low levels of GluSph, but this remains to be confirmed.

## 4. Conclusions

The first aim of this study was to develop and validate a new, robust and sensitive UPLC-MS/MS multiplex method for the analysis of 21 creatinine-normalized lysosphingolipid biomarkers in DUS according to the FDA recommendations. The second objective was to establish normal reference values to evaluate the clinical utility of this assay for high-risk screening, monitoring, and follow-up of patients for eight sphingolipidoses. This method provides an absolute quantification of lyso-Sulf, GluSph, GalSph, lyso-Gb_3_, lyso-GM1, lyso-GM2, and creatinine. The relative quantification of lyso-SM, seven related analogs of lyso-Gb_3_ and eight related analogs of GluSph was also performed. The results of this study using a urine filter paper matrix show the stability of urinary lysosphingolipids at 35 °C for 3 days, at room temperature and 4 °C for at least 26 days, and at −30 and −80 °C for at least 117 days. Moreover, the use of filter paper to collect, store and transport urine specimens to laboratories instead of using “liquid urine specimens” in sample tubes allows sample shipment by regular mail (avoiding the use of dry ice), thus reducing costs. Filter paper samples also facilitate long-term storage in the freezer. Furthermore, as demonstrated in the literature, urine filter paper collection can easily be performed at home for a neonatal or pediatric population [55,56,57,58,59,60,61,62,63]. The current results from Fabry and Gaucher patients show that using DUS to analyze lysosphingolipids can be a feasible approach applicable to the clinical field. These results show the importance of analyzing an analog profile related to lyso-Gb_3_ and GluSph to improve the biomarker sensitivity and reliability to monitor Fabry disease and Gaucher disease patients. Finally, another advantage is that the urine filter paper sample collection might facilitate patient recruitment for further collaborative studies on rare diseases.

However, this study has limitations. Except for the MLD patients, urine liquid samples were obtained from retrospective studies and were not directly collected on filter paper. The number of samples available from patients with MLD, Krabbe disease, ganglioside GM1, ganglioside GM2 and Niemann–Pick disease was small, thus limiting the interpretation of the clinical reliability of GalSph, lyso-GM1 and lyso-GM2, lyso-Sulf and lyso-SM in DUS. The interpretation of the results for lyso-Gb_3_ and its analog levels might be affected by the heterogeneity of the *GLA* variants in Fabry cohorts. It was not possible to compare the plasma, cerebrospinal fluid, dried blood spots and urine levels of these patients. Clinical information, such as the patient enzymatic activity, clinical manifestations and ethnicity, was limited and it was not possible to evaluate any further correlation. Future perspectives will involve the patients’ experience using filter paper urine sample collection. More clinical studies with larger cohorts are needed to measure these lysosphingolipids in urine samples from patients with various sphingolipidoses and gene variants.

## Figures and Tables

**Table 1:** UPLC and MS/MS parameters used for the analysis of the targeted lysosphingolipids and creatinine.

**UPLC Parameters**		**MS/MS Parameters**	
**Column**	**Halo^®^ HILIC 90 Å (USP: L3)**	**Ionization Mode**	**ESI+**
ID × Length	2.1 × 50 mm	Acquisition Mode	SRM
Particle Size	1.6 µm	Capillary Voltage	3.0 kV
Column Temperature	40 °C	Source Offset Voltage	90 V
Autosampler Temperature	10 °C	Source Temperature	150 °C
Flow Rate	0.8 mL/min	Desolvation Temperature	650 °C
Injection Volume	3 µL	Desolvation Gas Flow	1100 L/h
Weak Wash	1800 µL ACN	Cone Gas Flow	150 (L/h)
Strong Wash	600 µL 90/10 (H_2_O/ACN)	Collision Gas Flow	0.15 (mL/min)
Injection Mode	PLNO	Nebuliser Gas Flow	5.00 Bar
**Liquid Chromatographic Method**			
**Time (min)**	**Mobile Phase A**	**Mobile Phase B**	**Gradient types**
Initial	100%	0%	1 (Isocratic)
0.50	100%	0%	1 (Isocratic)
1.00	92%	8%	6 (Linear)
8.00	92%	8%	1 (Isocratic)
8.50	66%	34%	6 (Linear)
10.50	50%	50%	6 (Linear)
11.00	50%	50%	1 (Isocratic)
12.00	0%	100%	1 (Isocratic)
13.00	100%	0%	1 (Isocratic)

ID: internal diameter; PLNO: partial loop with needle overfill with fixed loop; UPLC: ultra-high-performance liquid chromatography; MS/MS: tandem mass spectrometry; HILIC: hydrophilic interaction chromatography; mobile phase A: (97/2.7/0.3 (ACN/H_2_O/FA) + 5 mm Amm. Form.); mobile phase B: (60/39.7/0.3 (ACN/H_2_O/FA) + 5 mm Amm. Form.); SRM: selected reaction monitoring; ACN: acetonitrile; H_2_O: water; ESI: electrospray ionisation.

**Table 2:** Selected reaction monitoring (SRM) transitions used to analyze the targeted lysosphingolipids and creatinine. The SRM transitions were separated into two detection windows according to their retention times to optimize the number of datapoints collected across the chromatographic peaks.

Compounds	Disease Targeted	SRM Transitions [M+H]^+^	Dwell Time	Cone	CE
Creatinine (M + 1)	n/a	*m*/*z* 115.07 > 45.05	0.01 s	30 V	5 V
d_3_-creatinine (M + 1)	ISTD	*m*/*z* 118.09 > 48.75	0.01 s	30 V	5 V
Lyso-Sulf	MLD	*m*/*z* 462.34 > 282.28	0.01 s	30 V	20 V
N-Gly-lyso-Sulf	ISTD	*m*/*z* 519.36 > 264.27	0.01 s	30 V	20 V
GalSph	Krabbe	*m*/*z* 462.34 > 282.28	0.01 s	30 V	20 V
GluSph	Gaucher	*m*/*z* 462.34 > 282.28	0.01 s	30 V	20 V
GluSph analog (−28)	Gaucher	*m*/*z* 434.31 > 254.25	0.01 s	30 V	20 V
GluSph analog (−26)	Gaucher	*m*/*z* 436.33 > 238.25	0.01 s	30 V	20 V
GluSph analog (−12)	Gaucher	*m*/*z* 450.31 > 252.23	0.01 s	30 V	20 V
GluSph analog (+2)	Gaucher	*m*/*z* 464.36 > 266.28	0.01 s	30 V	20 V
GluSph analog (+14)	Gaucher	*m*/*z* 476.32 > 278.25	0.01 s	30 V	20 V
GluSph analog (+16)	Gaucher	*m*/*z* 478.34 > 280.26	0.01 s	30 V	20 V
GluSph analog (+30)	Gaucher	*m*/*z* 492.32 > 294.24	0.01 s	30 V	20 V
GluSph analog (+32)	Gaucher	*m*/*z* 494.33 > 296.26	0.01 s	30 V	20 V
^13^C_6_-GluSph	ISTD	*m*/*z* 468.36 > 282.28	0.01 s	30 V	20 V
Lyso-SM	Niemann-Pick	*m*/*z* 465.35 > 184.07	0.01 s	30 V	21 V
D_9_-Lyso-SM	ISTD	*m*/*z* 474.40 > 193.13	0.01 s	30 V	21 V
Lyso-Gb_3_ analog (−28)	Fabry	*m*/*z* 758.42 > 254.25	0.01 s	30 V	35 V
Lyso-Gb_3_ analog (−12)	Fabry	*m*/*z* 774.41 > 252.23	0.01 s	30 V	35 V
Lyso-Gb_3_ analog (−2)	Fabry	*m*/*z* 784.43 > 280.26	0.01 s	30 V	35 V
Lyso-Gb_3_	Fabry	*m*/*z* 786.45 > 282.28	0.01 s	30 V	35 V
Lyso-Gb_3_ analog (+14)	Fabry	*m*/*z* 800.43 > 278.25	0.01 s	30 V	35 V
Lyso-Gb_3_ analog (+16)	Fabry	*m*/*z* 802.44 > 280.26	0.01 s	30 V	35 V
Lyso-Gb_3_ analog (+34)	Fabry	*m*/*z* 820.45 > 334.30	0.01 s	30 V	35 V
Lyso-Gb_3_ analog (+50)	Fabry	*m*/*z* 836.45 > 350.29	0.01 s	30 V	35 V
Lyso-GM2	GM2	*m*/*z* 1118.57 > 204.09	0.01 s	30 V	42 V
Lyso-GM1	GM1	*m*/*z* 1280.62 > 204.09	0.01 s	30 V	50 V
N-Gly-lyso-Gb_3_	ISTD	*m*/*z* 843.47 > 264.47	0.01 s	30 V	48 V

SRM: selected reaction monitoring; ISTD: internal standard; [M+H]^+^: protonated ion; CE: collision energy.

**Table 3:** Diagnostic reliability of lyso-Gb_3_, GluSph and the respective related analog evaluation according to their ROC curve results in untreated Fabry female (UFF), untreated Fabry male (UFM) and untreated Gaucher patients (UG) compared to healthy controls.

**Fabry ** **Disease**	**Biomarkers ** **Used**	**Lyso-Gb_3_** **Only**	**Lyso-Gb_3_** **Related Analogs Only**	**Lyso-Gb_3_** **and Its Related Analogs**
UFF	AUCs—ROC curve	0.811 ****	0.864 ****	0.877 ****
	95% CI	0.705 to 0.916	0.769 to 0.958	0.786 to 0.967
	Optimal cut-off value (pmol/mmol creatinine)	>5.36	>13.6	>27.4
	Sensitivity	63.6	72.7	75.8
	95% CI	46.6% to 77.8%	55.8% to 84.9%	59.0% to 87.2%
	Specificity	98.3	100	100
	95% CI	91.1% to 99.9%	94.0% to 100%	94.0% to 100%
	Highest Youden index value	0.619	0.727	0.758
UFM	AUCs—ROC curve	0.758 ***	0.921 ****	0.920 ****
	95% CI	0.610 to 0.907	0.823 to 1.00	0.821 to 1.00
	Optimal cut-off value (pmol/mmol creatinine)	>12.3	>40.6	>12.6
	Sensitivity	52.6	84.2	84.2
	95% CI	31.7% to 72.7%	62.4% to 94.5%	62.4% to 94.5%
	Specificity	98.3	100	98.3
	95% CI	91.1% to 99.9%	94.0% to 100%	91.1% to 99.9%
	Highest Youden index value	0.509	0.842	0.825
**Gaucher ** **Disease**	**Biomarkers ** **Used**	**GluSph ** **Only**	**GluSph** **Related Analogs Only**	**GluSph** **and its Related Analogs**
UG	AUCs—ROC curve	0.966 ****	1.000 ****	1.000 ****
	95% CI	0.925 to 1.00	1.000 to 1.000	1.000 to 1.000
	Optimal cut-off value (pmol/mmol creatinine)	>29.2	>85.5	>146
	Sensitivity	100	100	100
	95% CI	67.6% to 100%	67.6% to 100%	67.6% to 100%
	Specificity	91.7	100	98.3
	95% CI	83.8% to 97.3%	94.0% to 100%	91.1% to 99.9%
	Highest Youden index value	0.917	1.000	0.983

UFF: untreated Fabry female; UFM: untreated Fabry male; UG: untreated Gaucher 95%. CI: confidence interval; ***: *p*-value < 0.001; ****: *p*-value < 0.0001.

## Data Availability

The mass spectrometry data supporting these findings are provided in the Supplementary Materials and are stored into secured data repositories at the Faculty of Medicine and Health Sciences at the Université de Sherbrooke, Sherbrooke, Quebec.

## Supplementary Materials

The following supporting information can be downloaded at https://www.mdpi.com/article/10.3390/biom14121612/s1, Protocol S-1. Extraction recovery and matrix effect assays; Table S1. Demographic information of participants from the sphingolipidoses group to the study where the sex, mutation, phenotype, treatment, age and biomarker levels are described; Table S2: Demographic information of participants from the healthy and pathological control groups to the study where the sex, mutation, phenotype, treatment, age and biomarker levels are described; Table S3. Concentration of working solutions of the calibrators and QCs for the evaluation of the quality criteria of the methodology; Table S4. Intra- and interday accuracy and precision assays for the targeted sphingolipids and creatinine on urine filter paper; Table S5. Limits of detection, limits of quantification and evaluation of the linearity of the calibration curve using the coefficient of determination and Pearson correlation; Table S6. Extraction recovery and matrix effect assays for the targeted sphingolipids and creatinine on urine filter paper; Table S7. Long-term stability assays at several temperatures for the targeted sphingolipids and creatinine on urine filter paper; Table S8. Freeze–thaw cycle assays (n = 3) for the targeted sphingolipids and creatinine on urine filter paper at different concentrations for 0, 3 and 5 cycles; Table S9. Dilution factor assays (n = 5) for the targeted sphingolipids and creatinine on urine filter paper using a full, half and quarter 5 cm filter paper disk containing U-MQC; Table S10. Normal reference values were established according to the 95th percentile evaluation of lysosphingolipidose levels normalized to creatinine (pmol/mmol creatinine) in healthy control samples (n = 59); Table S11. Lysosphingolipids (A) lyso-Sulf, GalSph, lyso-GM2, lyso-GM1 and lyso-SM; (B) GluSph and the eight related analogs; (C) lyso-Gb_3_ and the seven related analogs; and (D) regrouped related analogs of lyso-Gb_3_ and GluSph levels in urine dried on filter paper normalized to creatinine (pmol/mmol creatinine) in sphingolipidoses (Fabry disease, Gaucher disease, MLD, GM1 and NPC) in pathological controls and healthy controls; Table S12. Kruskal–Wallis test and post hoc Dunn’s test statistical results for GluSph and its analogs in the CTRL, LSD, Sph, and Gaucher disease subgroups (treated Gaucher patients (TG) and untreated Gaucher patients (UG)); Table S13. Kruskal–Wallis test and post hoc Dunn’s test statistical results for GluSph and its analogs in CTRL, LSD, Sph, and Fabry disease subgroups (treated Fabry female (TFF), untreated Fabry female (UFF), treated Fabry male (TFM) and untreated Fabry male (UFM)); Table S14. Youden index evaluation of the ROC curve results obtained from (A) untreated Fabry female (UFF); (B) untreated Fabry male (UFM); and (C) untreated Gaucher (UG) patients.
