# Supplementary material for: UPLC-MS/MS High-Risk Screening for Sphingolipidoses Using Dried Urine Spots
Source: Biomolecules. 2024 Dec 17;14(12):1612. doi: 10.3390/biom14121612 (PMC11727146; doi:10.3390/biom14121612)
Supplement: Supplementary file 1 [file biomolecules-14-01612-s001.zip › Summary_Supplementary_Materials-October-30-2024.pdf]

Appendix A. Supplementary-Material

UPLC-MS/MS High Risk Screening for Sphingolipidoses Using Dried Urine Spots

Tristan Martineau[1], Bruno Maranda[1], and Christiane Auray-Blais[1*]

1 Division of Medical Genetics, Department of Pediatrics, Faculty of Medicine and Health Sciences, Université de Sherbrooke, Centre de recherche-CHUS, 3001, 12th Avenue North, Sherbrooke, Quebec, Canada J1H 5N4

.*Correspondence: Christiane.auray-blais@usherbrooke.ca; +1-819-346-1110, ext. 14706, FAX: +1-819-564-5217

Protocol S-1. Extraction recovery and matrix effect assays;

Table S-1. Demographic information of participants from the sphingolipidoses group to the study where sex, mutation, phenotype, treatment, age and biomarker levels are described;

Table S-2. Demographic information of participants from healthy and pathological control groups to the study where sex, mutation, phenotype, treatment, age and biomarker levels are described;

Table S-3. Concentration of working solutions of calibrators and QCs for the evaluation of quality criteria of the methodology

Table S- 4. Intra- and interday accuracy and precision assays for the targeted sphingolipids and creatinine on urine filter paper;

Table S-5. Limits of detection, limits of quantification and evaluation of the linearity of the calibration curve using coefficient of determination and Pearson correlation;

Table S-6. Extraction recovery and matrix effect assays for the targeted sphingolipids and creatinine on urine filter paper;

Table S-7. Long term stability assays at several temperatures for the targeted sphingolipids and creatinine on urine filter paper;

Table S-8. Freeze thaw cycle assays (n=3) for the targeted sphingolipids and creatinine on urine filter paper at different concentrations for 0, 3 and 5 cycles;

Table S-9. Dilution factor assays (n=5) for the targeted sphingolipids and creatinine on urine filter paper using full, half and quarter of the 5-cm filter paper disk containing U-MQC;

Table S-10. Normal reference values were established according to the 95th percentile evaluation of lysosphingolipidose levels normalized to creatinine (pmol/mmol creatinine) in healthy control samples (n=59);

Table S-11. Lysosphingolipids (A) Lyso-Sulf, GalSph, Lyso-GM2, Lyso-GM1 and Lyso-SM; B) GluSph and his eight related analogs; C) Lyso-Gb_3 and his seven related analogs; and D) Regrouped related analogs of Lyso-Gb_3 and GluSph) levels in urine dried on filter paper normalized to creatinine (pmol/mmol creatinine) in sphingolipidoses (Fabry disease, Gaucher disease, MLD, GM1 and NPC), in pathological controls and healthy controls;

Table S-12. Kruskal-Wallis test and posthoc Dunn's test statistical results for GluSph and its analogs in CTRL, LSD, Sph, and Gaucher disease subgroups (Treated Gaucher patients (TG) and Untreated Gaucher patients (UG));

Table S-13. Kruskal-Wallis test and posthoc Dunn's test statistical results for GluSph and its analogs in CTRL, LSD, Sph, and Fabry disease subgroups (Treated Fabry Female (TFF), Untreated Fabry Female (UFF), Treated Fabry Male (TFM) and Untreated Fabry Male (UFM);

Table S-14. Youden index evaluation of the ROC curve results obtained from: A) Untreated Fabry Female (UFF); B) Untreated Fabry Male (UFM); and C) Untreated Gaucher patients (UG).

Protocol S-1.

Three groups of samples were used: Group A represents S-LQCs, S-MQCs and S-HQCs (n=3); Group B represents equivalent matrix containing same concentration of metabolites as group A, but compounds were spiked in SPE post-extraction (n=3 by QC levels), and Group C represents blank extracts spiked with equivalent concentration of metabolites in SPE post-extraction (n=3 by QC levels). For the group A and B, to evaluate extraction recovery, responses (peak area/internal standard peak area) from group A and B were compared as described in equation (1):

$$Extraction\ Recovery = \frac{B}{A}\ x\ 100\% \quad (1)$$

To evaluate ion suppression or enhancement from matrix effect, responses (peak area/internal standard peak area) from group B and C were compared as described in equation (2):

$$Matrix\ Effect = \left(1 - \frac{B}{C}\right)\ x\ 100\% \quad (2)$$

To evaluate creatinine recovery, endogenous creatinine response was subtracted from spiked samples to eliminate the endogenous impact.

Table S-1.

Sphingolipidoses (Sph)								Measured levels (pmol/mmol creatinine)																									
Groups	Groups	Gender	Mutation- Variant	Type of mutation	Likely phenotype	Treatment	Age	Lyso-Sulf (+)	GluSph	GluSph (-28)	GluSph (-26)	GluSph (-12)	GluSph (+2)	GluSph (+14)	GluSph (+16)	GluSph (+30)	GluSph (+32)	Analogues	Combined	GalSph	Lyso-Gb3	Lyso-Gb3 (-28)	Lyso-Gb3 (-12)	Lyso-Gb3 (-2)	Lyso-Gb3 (+14)	Lyso-Gb3 (+16)	Lyso-Gb3 (+34)	Lyso-Gb3 (+50)	Analogues	Combined	Lyso-GM2	Lyso-GM1	Lyso-SM
Fabry Disease	UFF	female	A143P	Missence	Classical	No	49,7	0,0	9,0	0,0	0,0	0,0	0,0	0,0	0,0	0,0	0,0	0,0	9,0	0,0	19,0	0,0	97,0	0,0	0,0	82,0	64,0	37,0	280,0	299,0	0,0	0,0	38,0
		female	A348P	Missence	Classical	No	58,7	0,0	7,0	0,0	0,0	0,0	0,0	0,0	0,0	0,0	0,0	0,0	7,0	0,0	88,0	0,0	0,0	0,0	0,0	50,0	89,0	57,0	196,0	284,0	0,0	0,0	36,0
		female	A348P	Missence	Classical	No	55,1	0,0	9,0	0,0	0,0	0,0	0,0	0,0	0,0	0,0	0,0	0,0	9,0	0,0	0,0	0,0	0,0	0,0	0,0	0,0	30,0	0,0	30,0	30,0	0,0	0,0	37,0
		female	A348P	Missence	Classical	No	45,2	0,0	9,0	0,0	0,0	0,0	0,0	0,0	0,0	0,0	0,0	0,0	9,0	0,0	15,0	0,0	0,0	0,0	0,0	0,0	27,0	0,0	27,0	42,0	0,0	0,0	42,0
		female	c.640-2A>G	Intronic	Classical	No	26,3	0,0	24,0	0,0	0,0	0,0	0,0	0,0	0,0	0,0	0,0	0,0	24,0	0,0	67,0	0,0	101,0	0,0	0,0	109,0	76,0	40,0	326,0	393,0	0,0	0,0	66,0
		female	c.802-3_802-2delCA	Deletion	Classical	No	20,3	0,0	15,0	0,0	0,0	0,0	0,0	0,0	0,0	0,0	0,0	0,0	15,0	0,0	19,0	0,0	93,0	0,0	0,0	90,0	98,0	59,0	340,0	359,0	0,0	0,0	46,0
		female	G43V	Missence	Classical	No	37,2	0,0	19,0	0,0	0,0	0,0	0,0	0,0	0,0	0,0	0,0	0,0	19,0	0,0	67,0	0,0	67,0	0,0	0,0	80,0	79,0	37,0	263,0	330,0	0,0	0,0	50,0
		female	L106P (fs*13)	Deletion	Classical	No	38,2	0,0	34,0	0,0	0,0	0,0	0,0	0,0	0,0	0,0	0,0	0,0	34,0	0,0	47,0	0,0	0,0	0,0	0,0	144,0	145,0	0,0	289,0	336,0	0,0	0,0	116,0
		female	L191Q	Missence	Classical	No	43,9	0,0	0,0	0,0	0,0	0,0	0,0	0,0	0,0	0,0	0,0	0,0	0,0	0,0	0,0	0,0	0,0	0,0	0,0	0,0	0,0	0,0	0,0	0,0	0,0	0,0	266,0
		female	L191Q	Missence	Classical	No	23,0	0,0	54,0	0,0	0,0	0,0	0,0	0,0	0,0	0,0	0,0	0,0	54,0	0,0	24,0	0,0	69,0	0,0	20,0	92,0	71,0	31,0	283,0	307,0	0,0	0,0	69,0
		female	L191Q	Missence	Classical	No	19,9	0,0	45,0	0,0	0,0	0,0	0,0	0,0	0,0	0,0	0,0	0,0	45,0	0,0	86,0	0,0	97,0	0,0	51,0	288,0	382,0	164,0	982,0	1068,0	0,0	0,0	95,0
		female	P293T	Missence	Classical	No	36,5	0,0	8,0	0,0	0,0	0,0	0,0	0,0	0,0	0,0	0,0	0,0	8,0	0,0	14,0	0,0	45,0	0,0	10,0	52,0	40,0	28,0	175,0	189,0	0,0	0,0	22,0
		female	P293T	Missence	Classical	No	40,3	0,0	8,0	0,0	0,0	0,0	0,0	0,0	0,0	0,0	0,0	0,0	8,0	0,0	11,0	0,0	0,0	0,0	0,0	38,0	37,0	33,0	108,0	119,0	0,0	0,0	27,0
		female	Q321E	Missence	Classical	No	20,7	0,0	11,0	0,0	0,0	0,0	0,0	0,0	0,0	0,0	0,0	0,0	11,0	0,0	21,0	0,0	0,0	0,0	0,0	26,0	34,0	27,0	87,0	108,0	0,0	0,0	40,0
		female	Q321E	Missence	Classical	No	62,4	0,0	0,0	0,0	0,0	0,0	0,0	0,0	0,0	0,0	831,0	831,0	831,0	0,0	18,0	0,0	0,0	0,0	0,0	48,0	39,0	0,0	87,0	105,0	0,0	0,0	50,0
		female	Q321R	Missence	Classical	No	48,5	0,0	43,0	0,0	0,0	0,0	0,0	0,0	0,0	0,0	0,0	0,0	43,0	0,0	69,0	0,0	0,0	0,0	0,0	0,0	0,0	0,0	0,0	69,0	0,0	0,0	182,0
		female	R220X	Nonsense	Classical	No	37,1	0,0	11,0	0,0	0,0	0,0	0,0	0,0	0,0	0,0	0,0	0,0	11,0	0,0	29,0	0,0	0,0	0,0	0,0	61,0	42,0	22,0	125,0	154,0	0,0	0,0	36,0
		female	R220X	Nonsense	Classical	No	59,2	0,0	36,0	0,0	0,0	0,0	0,0	0,0	0,0	0,0	0,0	0,0	36,0	0,0	0,0	0,0	0,0	0,0	0,0	0,0	0,0	0,0	0,0	0,0	0,0	0,0	157,0
		female	R227Q	Missence	Classical	No	27,3	0,0	57,0	0,0	0,0	0,0	0,0	0,0	0,0	0,0	0,0	0,0	57,0	0,0	114,0	0,0	0,0	0,0	0,0	0,0	280,0	0,0	280,0	394,0	0,0	0,0	306,0
		female	R332D (fs*16)	Deletion	Classical	No	20,2	0,0	9,0	0,0	0,0	0,0	0,0	0,0	0,0	0,0	0,0	0,0	9,0	0,0	40,0	0,0	0,0	0,0	10,0	50,0	32,0	13,0	105,0	145,0	0,0	0,0	27,0
		female	R332D (fs*16)	Deletion	Classical	No	26,0	0,0	12,0	0,0	0,0	0,0	0,0	0,0	0,0	0,0	0,0	0,0	12,0	0,0	43,0	0,0	0,0	0,0	0,0	34,0	30,0	0,0	64,0	107,0	0,0	0,0	48,0
		female	R342Q	Missence	Classical	No	36,1	0,0	0,0	0,0	0,0	0,0	0,0	0,0	0,0	0,0	0,0	0,0	0,0	0,0	48,0	0,0	0,0	0,0	0,0	77,0	68,0	39,0	184,0	232,0	0,0	0,0	43,0
		female	R342X	Nonsense	Classical	No	22,5	0,0	13,0	0,0	0,0	0,0	0,0	0,0	0,0	0,0	0,0	0,0	13,0	0,0	52,0	0,0	97,0	0,0	32,0	131,0	180,0	57,0	497,0	549,0	0,0	0,0	36,0
		female	S345P	Missence	Classical	No	33,2	0,0	0,0	0,0	0,0	0,0	0,0	0,0	0,0	0,0	0,0	0,0	0,0	0,0	0,0	0,0	0,0	0,0	0,0	0,0	0,0	0,0	0,0	0,0	0,0	0,0	130,0
		female	S401F (fs*?)	Insertion	Classical	No	13,6	0,0	18,0	0,0	0,0	0,0	0,0	0,0	0,0	0,0	0,0	0,0	18,0	0,0	0,0	0,0	0,0	0,0	0,0	0,0	57,0	0,0	57,0	57,0	0,0	0,0	62,0
		female	S401F (fs*?)	Insertion	Classical	No	8,4	0,0	24,0	0,0	0,0	0,0	0,0	0,0	0,0	0,0	0,0	0,0	24,0	0,0	0,0	0,0	0,0	0,0	0,0	114,0	260,0	88,0	462,0	462,0	0,0	0,0	102,0
		female	S401F (fs*?)	Insertion	Classical	No	20,8	0,0	23,0	0,0	0,0	0,0	0,0	0,0	0,0	0,0	0,0	0,0	23,0	0,0	21,0	0,0	0,0	0,0	0,0	61,0	29,0	0,0	90,0	111,0	0,0	0,0	32,0
		Female	c.639+919G>A	Intronic	Late-onset	No	34,4	0,0	0,0	0,0	0,0	0,0	0,0	0,0	0,0	0,0	0,0	0,0	0,0	0,0	0,0	0,0	0,0	0,0	0,0	0,0	0,0	0,0	0,0	0,0	0,0	0,0	756,0
		Female	c.640-801G>A	Missense	Late-onset	No	43,5	0,0	32,0	0,0	0,0	0,0	0,0	0,0	0,0	0,0	0,0	0,0	32,0	0,0	0,0	0,0	0,0	0,0	0,0	0,0	0,0	0,0	0,0	0,0	0,0	0,0	118,0
		Female	c.640-801G>A	Missense	Late-onset	No	11,1	0,0	7,0	0,0	0,0	0,0	0,0	0,0	0,0	0,0	0,0	0,0	7,0	0,0	0,0	0,0	0,0	0,0	0,0	0,0	0,0	0,0	0,0	0,0	0,0	0,0	51,0
		Female	H46P	Intronic	Late-onset	No	52,1	0,0	0,0	0,0	0,0	0,0	0,0	0,0	0,0	0,0	0,0	0,0	0,0	0,0	0,0	0,0	0,0	0,0	0,0	0,0	0,0	0,0	0,0	0,0	0,0	0,0	60,0
		Female	H46P	Missense	Late-onset	No	18,3	0,0	18,0	0,0	0,0	0,0	0,0	0,0	0,0	0,0	0,0	0,0	18,0	0,0	0,0	0,0	0,0	0,0	0,0	0,0	0,0	0,0	0,0	0,0	0,0	0,0	61,0
		Female	N215S	Intronic	Late-onset	No	28,9	0,0	28,0	0,0	0,0	0,0	0,0	0,0	0,0	0,0	0,0	0,0	28,0	0,0	0,0	88,0	0,0	0,0	0,0	0,0	0,0	0,0	88,0	88,0	0,0	0,0	68,0
	TFF	Female	A285D	Missence	Classical	Fabrazyme	47,3	0,0	19,0	0,0	0,0	0,0	0,0	0,0	0,0	0,0	0,0	0,0	19,0	0,0	0,0	0,0	0,0	0,0	0,0	0,0	65,0	0,0	65,0	65,0	0,0	0,0	92,0
		Female	A348P	Missence	Classical	Fabrazyme	45,8	0,0	38,0	0,0	0,0	0,0	0,0	0,0	0,0	0,0	0,0	0,0	38,0	0,0	0,0	0,0	0,0	0,0	0,0	0,0	0,0	0,0	0,0	0,0	0,0	0,0	162,0
		Female	A348P	Missence	Classical	Replagal	53,5	0,0	10,0	0,0	0,0	0,0	0,0	0,0	0,0	0,0	0,0	0,0	10,0	0,0	63,0	0,0	0,0	0,0	0,0	123,0	70,0	0,0	193,0	256,0	0,0	0,0	40,0
		Female	A348P	Missence	Classical	Replagal	50,6	0,0	21,0	0,0	0,0	0,0	0,0	0,0	0,0	0,0	0,0	0,0	21,0	0,0	0,0	0,0	0,0	0,0	0,0	0,0	0,0	0,0	0,0	0,0	0,0	0,0	71,0
		Female	D101F (fs*21)	Deletion	Classical	Fabrazyme	50,8	0,0	41,0	0,0	0,0	0,0	0,0	0,0	0,0	0,0	0,0	0,0	41,0	0,0	32,0	0,0	0,0	0,0	0,0	128,0	155,0	145,0	428,0	460,0	0,0	0,0	88,0
		Female	E338K	Missence	Classical	Replagal	44,4	0,0	0,0	0,0	0,0	0,0	0,0	0,0	0,0	0,0	0,0	0,0	0,0	0,0	0,0	0,0	0,0	0,0	0,0	0,0	49,0	0,0	49,0	49,0	0,0	0,0	82,0
		Female	G43V	Missence	Classical	Fabrazyme	63,3	0,0	0,0	0,0	0,0	0,0	0,0	0,0	0,0	0,0	0,0	0,0	0,0	0,0	0,0	0,0	0,0	0,0	0,0	47,0	49,0	0,0	96,0	96,0	0,0	0,0	70,0
		Female	L414S	Missence	Classical	Fabrazyme	44,4	0,0	6,0	0,0	0,0	0,0	0,0	0,0	0,0	0,0	0,0	0,0	6,0	0,0	0,0	0,0	90,0	0,0	47,0	279,0	77,0	33,0	526,0	526,0	0,0	0,0	17,0
		Female	L414S	Missence	Classical	Galafold	38,5	0,0	6,0	0,0	0,0	0,0	0,0	0,0	0,0	0,0	0,0	0,0	6,0	0,0	56,0	0,0	91,0	0,0	19,0	126,0	126,0	49,0	411,0	467,0	0,0	0,0	30,0
		Female	L414S	Missence	Classical	Replagal	61,4	0,0	37,0	0,0	0,0	0,0	0,0	0,0	0,0	0,0	0,0	0,0	37,0	0,0	22,0	0,0	138,0	0,0	42,0	147,0	91,0	59,0	477,0	499,0	0,0	0,0	80,0
		Female	M187V (fs*6)	Deletion	Classical	Fabrazyme	53,0	0,0	12,0	0,0	0,0	0,0	0,0	0,0	0,0	0,0	0,0	0,0	12,0	0,0	27,0	0,0	88,0	0,0	0,0	118,0	105,0	64,0	375,0	402,0	0,0	0,0	45,0
		Female	c.640-2A>G	Intronic	Classical	Replagal	61,2	0,0	8,0	0,0	0,0	0,0	0,0	0,0	0,0	0,0	0,0	0,0	8,0	0,0	26,0	0,0	89,0	0,0	29,0	152,0	91,0	55,0	416,0	442,0	0,0	0,0	34,0
		Female	P293T	Missence	Classical	Galafold	55,5	0,0	16,0	11,0	0,0	0,0	0,0	0,0	0,0	0,0	0,0	11,0	27,0	0,0	38,0	0,0	0,0	0,0	0,0	51,0	156,0	77,0	284,0	322,0	0,0	0,0	71,0
		Female	P293T	Missence	Classical	Galafold	63,0	0,0	10,0	0,0	0,0	0,0	0,0	0,0	0,0	0,0	0,0	0,0	10,0	0,0	24,0	0,0	63,0	0,0	0,0	69,0	101,0	41,0	274,0	298,0	0,0	0,0	41,0
		Female	Q321R	Missence	Classical	Replagal	56,4	0,0	25,0	0,0	0,0	0,0	0,0	0,0	0,0	0,0	0,0	0,0	25,0	0,0	49,0	0,0	163,0	0,0	88,0	331,0	154,0	109,0	845,0	894,0	0,0	0,0	82,0
		Female	R220X	Nonsense	Classical	Replagal	61,5	0,0	33,0	0,0	0,0	0,0	0,0	0,0	0,0	0,0	0,0	0,0	33,0	0,0	0,0	0,0	0,0	0,0	0,0	0,0	0,0	0,0	0,0	0,0	0,0	0,0	129,0
		Female	S345P	Missence	Classical	Replagal	62,4	0,0	81,0	0,0	0,0	0,0	0,0	0,0	0,0	0,0	0,0	0,0	81,0	0,0	0,0	0,0	0,0	0,0	0,0	0,0	140,0	0,0	140,0	140,0	0,0	0,0	304,0
		Female	S345P	Missence	Classical	Galafold	59,2	0,0	0,0	0,0	0,0	0,0	0,0	0,0	0,0	0,0	0,0	0,0	0,0	0,0	0,0	0,0	0,0	0,0	0,0	0,0	79,0	0,0	79,0	79,0	0,0	0,0	132,0
		Female	V254del	Deletion	Classical	Galafold	61,1	0,0	13,0	0,0	0,0	0,0	0,0	0,0	0,0	0,0	0,0	0,0	13,0	0,0	31,0	0,0	0,0	0,0	0,0	48,0	75,0	74,0	197,0	228,0	0,0	0,0	55,0
		Female	W81S	Missence	Classical	Replagal	71,4	0,0	7,0	0,0	0,0	0,0	0,0	0,0	0,0	0,0	0,0	0,0	7,0	0,0	20,0	0,0	72,0	0,0	29,0	128,0	90,0	53,0	372,0	392,0	0,0	0,0	36,0
		Female	Y134S	Missence	Classical	Replagal	58,1	0,0	20,0	0,0	0,0	0,0	0,0	0,0	0,0	0,0	0,0	0,0	20,0	0,0	0,0	0,0	0,0	0,0	0,0	58,0	91,0	75,0	224,0	224,0	0,0	0,0	79,0
		Female	P293L	Missence	Late-onset	Fabrazyme	62,0	0,0	26,0	0,0	0,0	0,0	0,0	0,0	0,0	0,0	0,0	0,0	26,0	0,0	0,0	0,0	0,0	0,0	0,0	78,0	0,0	0,0	78,0	78,0	0,0	0,0	99,0
		Female	c.639+919G>A	Intronic	Late-onset	Replagal	63,2	0,0	0,0	0,0	0,0	0,0	0,0	0,0	0,0	0,0	0,0	0,0	0,0	0,0	0,0	0,0	0,0	0,0	0,0	0,0	0,0	0,0	0,0	0,0	0,0	0,0	85,0
	UFM	Male	S345P	Missence	Classical	No	14,7	0,0	0,0	0,0	0,0	0,0	0,0	0,0	0,0	0,0	0,0	0,0	0,0	0,0	0,0	0,0	379,0	0,0	107,0	468,0	1033,0	2251,0	4238,0	4238,0	0,0	0,0	126,0
		Male	R342Q	Missence	Classical	No	15,2	0,0	0,0	0,0	0,0	0,0	0,0	0,0	0,0	0,0	0,0	0,0	0,0	0,0	101,0	136,0	772,0	178,0	889,0	3744,0	1432,0	3598,0	10749,0	10850,0	0,0	0,0	62,0
		Male	R112C	Missence	Classical	No	15,9	0,0	0,0	0,0	0,0	0,0	0,0	22,0	63,0	40,0	0,0	125,0	125,0	0,0	46,0	23,0	109,0	21,0	114,0	690,0	279,0	177,0	1413,0	1459,0	0,0	0,0	37,0
		Male	W349X	Nonsense	Classical	No	10,5	0,0	0,0	0,0	0,0	0,0	0,0	0,0	0,0	0,0	0,0	0,0	0,0	0,0	54,0	144,0	844,0	0,0	679,0	2247,0	1852,0	3564,0	9330,0	9384,0	0,0	0,0	90,0
		Male	W349X	Nonsense	Classical	No	8,6	0,0	0,0	0,0	0,0	0,0	0,0	0,0	0,0	0,0	0,0	0,0	0,0	0,0	44,0	0,0	593,0	0,0	310,0	1039,0	1065,0	3258,0	6265,0	6309,0	0,0	0,0	138,0
		Male	S345P	Missence	Classical	No	23,9	0,0	0,0	0,0	0,0	0,0	0,0	0,0	0,0	0,0	0,0	0,0	0,0	0,0	0,0	0,0	0,0	0,0	0,0	0,0	157,0	230,0	387,0	387,0	0,0	0,0	162,0
		Male	R112C	Missence	Classical	No	25,8	0,0	0,0	0,0	0,0	0,0	0,0	0,0	0,0	0,0	0,0	0,0	0,0	0,0	0,0	0,0	0,0	0,0	0,0	107,0	120,0	182,0	409,0	409,0	0,0	0,0	148,0
		Male	Q321E	Missence	Classical	No	31,7	0,0	0,0	0,0	0,0	0,0	0,0	0,0	0,0	0,0	0,0	0,0	0,0	0,0	56,0	39,0	134,0	0,0	125,0	627,0	264,0	546,0	1735,0	1791,0	0,0	0,0	46,0
		Male	R220X	Nonsense	Classical	No	40,1	0,0	0,0	0,0	0,0	0,0	0,0	0,0	0,0	0,0	0,0	0,0	0,0	0,0	108,0	0,0	390,0	0,0	210,0	1500,0	1533,0	3531,0	7164,0	7272,0	0,0	0,0	153,0
		Male	Q321X	Nonsense	Classical	No	48,8	0,0	22,0	0,0	0,0	0,0	0,0	0,0	0,0	0,0	0,0	0,0	22,0	0,0	491,0	286,0	2212,0	497,0	1089,0	5048,0	1906,0	3853,0	14891,0	15382,0	0,0	0,0	102,0
		Male	Y134S	Missence	Classical	No	28,1	0,0	12,0	0,0	0,0	0,0	0,0	0,0	0,0	0,0	0,0	0,0	12,0	0,0	364,0	163,0	763,0	144,0	343,0	1364,0	465,0	1198,0	4440,0	4804,0	0,0	0,0	75,0
		Male	c.640-801G>A	Intronic	Late-onset	No	13,9	0,0	0,0	0,0	0,0	0,0	0,0	0,0	0,0	0,0	0,0	0,0	0,0	0,0	0,0	0,0	0,0	0,0	0,0	71,0	51,0	48,0	170,0	170,0	0,0	0,0	43,0
		Male	R356W	Missence	Late-onset	No	19,9	0,0	0,0	0,0	0,0	0,0	0,0	0,0	0,0	0,0	0,0	0,0	0,0	0,0	0,0	0,0	0,0	0,0	0,0	0,0	0,0	0,0	0,0	0,0	0,0	0,0	92,0
		Male	N215S	Missence	Late-onset	No	59,3	0,0	0,0	0,0	0,0	0,0	0,0	0,0	0,0	0,0	0,0	0,0	0,0	0,0	27,0	0,0	0,0	0,0	0,0	81,0	0,0	0,0	81,0	108,0	0,0	0,0	95,0
		Male	N215S	Missence	Late-onset	No	39,7	0,0	0,0	0,0	0,0	0,0	0,0	0,0	0,0	0,0	0,0	0,0	0,0	0,0	0,0	0,0	0,0	0,0	0,0	66,0	42,0	37,0	145,0	145,0	0,0	0,0	50,0
		Male	R356W	Missence	Late-onset	No	32,0	0,0	0,0	0,0	0,0	0,0	0,0	0,0	0,0	0,0	0,0	0,0	0,0	0,0	0,0	0,0	0,0	0,0	0,0	41,0	57,0	0,0	98,0	98,0	0,0	0,0	60,0
		Male	c.639+919G>A	Intronic	Late-onset	No	40,5	0,0	0,0	0,0	0,0	0,0	0,0	0,0	0,0	0,0	0,0	0,0	0,0	0,0	0,0	0,0	0,0	0,0	0,0	0,0	0,0	0,0	0,0	0,0	0,0	0,0	258,0
		Male	c.639+919G>A	Intronic	Late-onset	No	34,6	0,0	0,0	0,0	0,0	0,0	0,0	0,0	0,0	0,0	0,0	0,0	0,0	0,0	0,0	0,0	0,0	0,0	0,0	0,0	0,0	0,0	0,0	0,0	0,0	0,0	187,0
		Male	N215S	Missence	Late-onset	No	66,9	0,0	0,0	0,0	0,0	0,0	0,0	0,0	0,0	0,0	0,0	0,0	0,0	0,0	24,0	0,0	103,0	0,0	21,0	120,0	36,0	26,0	306,0	330,0	0,0	0,0	40,0
	TFM	Male	Q386X	Nonsense	Classical	Replagal	43,4	0,0	0,0	0,0	0,0	0,0	0,0	0,0	0,0	0,0	0,0	0,0	0,0	0,0	33,0	0,0	178,0	0,0	0,0	225,0	143,0	88,0	634,0	667,0	0,0	0,0	97,0
		Male	S345P	Missence	Classical	Replagal	58,5	0,0	0,0	0,0	0,0	0,0	0,0	0,0	0,0	0,0	0,0	0,0	0,0	0,0	0,0	0,0	0,0	0,0	0,0	80,0	150,0	138,0	368,0	368,0	0,0	0,0	104,0
		Male	R227Q	Missence	Classical	Fabrazyme	48,4	0,0	0,0	0,0	0,0	0,0	0,0	0,0	0,0	0,0	0,0	0,0	0,0	0,0	0,0	0,0	0,0	0,0	0,0	165,0	201,0	154,0	520,0	520,0	0,0	0,0	160,0
		Male	L414S	Missence	Classical	Fabrazyme	40,9	0,0	21,0	0,0	0,0	0,0	0,0	0,0	0,0	0,0	0,0	0,0	21,0	0,0	122,0	100,0	467,0	95,0	126,0	768,0	215,0	143,0	1914,0	2036,0	0,0	0,0	106,0
		Male	A348P	Missence	Classical	Fabrazyme	43,0	0,0	26,0	0,0	0,0	0,0	0,0	0,0	0,0	0,0	0,0	0,0	26,0	0,0	93,0	0,0	0,0	0,0	0,0	178,0	0,0	0,0	178,0	271,0	0,0	0,0	127,0
		Male	C12F (fs*105)	Deletion	Classical	Fabrazyme	45,3	0,0	15,0	0,0	0,0	0,0	0,0	0,0	0,0	0,0	0,0	0,0	15,0	0,0	0,0	0,0	120,0	0,0	41,0	211,0	99,0	73,0	544,0	544,0	0,0	0,0	67,0
		Male	W91S	Missence	Classical	Replagal	53,5	0,0	12,0	0,0	0,0	0,0	0,0	0,0	0,0	0,0	0,0	0,0	12,0	0,0	0,0	0,0	188,0	0,0	53,0	363,0	318,0	313,0	1235,0	1235,0	0,0	0,0	69,0
		Male	R342X	Nonsense	Classical	Replagal	45,0	0,0	0,0	0,0	0,0	0,0	0,0	0,0	0,0	0,0	0,0	0,0	0,0	0,0	136,0	0,0	187,0	0,0	85,0	338,0	179,0	139,0	928,0	1064,0	0,0	0,0	128,0
		Male	A348P	Missence	Classical	Fabrazyme	28,4	0,0	12,0	0,0	0,0	0,0	0,0	0,0	0,0	0,0	0,0	0,0	12,0	0,0	104,0	58,0	377,0	42,0	191,0	1124,0	795,0	939,0	3526,0	3630,0	0,0	0,0	59,0
		Male	M1T	Missence	Classical	Fabrazyme	60,1	0,0	23,0	0,0	0,0	0,0	0,0	0,0	36,0	0,0	0,0	36,0	59,0	0,0	233,0	79,0	554,0	142,0	479,0	2048,0	475,0	341,0	4118,0	4351,0	0,0	0,0	70,0
		Male	A348P	Missence	Classical	Fabrazyme	26,6	0,0	10,0	0,0	0,0	0,0	0,0	0,0	0,0	0,0	0,0	0,0	10,0	0,0	26,0	0,0	0,0	0,0	0,0	76,0	67,0	51,0	194,0	220,0	0,0	0,0	55,0
		Male	A348P	Missence	Classical	Fabrazyme	25,1	0,0	9,0	0,0	0,0	0,0	0,0	0,0	0,0	0,0	0,0	0,0	9,0	0,0	0,0	0,0	58,0	0,0	19,0	85,0	38,0	28,0	228,0	228,0	0,0	0,0	41,0
		Male	A348P	Missence	Classical	Fabrazyme	26,4	0,0	7,0	0,0	0,0	0,0	0,0	0,0	23,0	18,0	0,0	41,0	48,0	0,0	9,0	0,0	0,0	0,0	17,0	101,0	38,0	21,0	177,0	186,0	0,0	0,0	34,0
		Male	M290T	Missence	Classical	Galafold	54,8	0,0	14,0	0,0	0,0	0,0	0,0	30,0	0,0	0,0	0,0	30,0	44,0	0,0	73,0	81,0	352,0	0,0	90,0	566,0	230,0	214,0	1533,0	1606,0	0,0	0,0	66,0
		Male	Q321R	Missence	Classical	Fabrazyme	50,7	0,0	9,0	0,0	0,0	0,0	0,0	0,0	0,0	0,0	0,0	0,0	9,0	0,0	21,0	0,0	123,0	0,0	30,0	131,0	133,0	62,0	479,0	500,0	0,0	0,0	49,0
		Male	A143P	Missence	Classical	Replagal	54,0	0,0	0,0	0,0	0,0	0,0	0,0	0,0	0,0	0,0	0,0	0,0	0,0	0,0	0,0	0,0	0,0	0,0	0,0	153,0	208,0	149,0	510,0	510,0	0,0	0,0	109,0
		Male	L191Q	Missence	Classical	Replagal	52,1	0,0	16,0	0,0	0,0	0,0	0,0	0,0	0,0	0,0	0,0	0,0	16,0	0,0	200,0	71,0	336,0	140,0	172,0	787,0	252,0	161,0	1919,0	2119,0	0,0	0,0	81,0
		Male	P293L	Missence	Classical	Replagal	39,7	0,0	0,0	0,0	0,0	0,0	0,0	0,0	0,0	0,0	0,0	0,0	0,0	0,0	109,0	0,0	228,0	0,0	0,0	347,0	0,0	267,0	842,0	951,0	0,0	0,0	211,0
		Male	A348P	Missence	Classical	Fabrazyme	44,6	0,0	8,0	0,0	0,0	0,0	0,0	0,0	0,0	0,0	0,0	0,0	8,0	0,0	24,0	0,0	83,0	0,0	44,0	289,0	158,0	103,0	677,0	701,0	0,0	0,0	46,0
		Male	R220X	Nonsense	Classical	Fabrazyme	27,6	0,0	21,0	0,0	0,0	0,0	0,0	0,0	0,0	0,0	0,0	0,0	21,0	0,0	37,0	0,0	0,0	0,0	0,0	214,0	374,0	168,0	756,0	793,0	0,0	0,0	104,0
		Male	N215S	Missence	Late-onset	Galafold	41,3	0,0	5,0	0,0	0,0	0,0	0,0	0,0	0,0	0,0	0,0	0,0	5,0	0,0	12,0	0,0	0,0	0,0	0,0	35,0	15,0	0,0	50,0	62,0	0,0	0,0	26,0
		Male	N215S	Missence	Late-onset	Galafold	66,4	0,0	9,0	0,0	0,0	0,0	0,0	0,0	0,0	0,0	0,0	0,0	9,0	0,0	0,0	0,0	0,0	0,0	0,0	26,0	29,0	0,0	55,0	55,0	0,0	0,0	42,0
Gaucher Disease	UG	female	N370S/N370S	Missense	Type I	No	48.3	0,0	31,5	17,4	2529,4	1001,8	936,8	421,3	1123,4	2413,4	133,0	8576,6	8608,1	0,0	0,0	0,0	0,0	0,0	0,0	0,0	0,0	0,0	0,0	0,0	0,0	0,0	43,0
		female	N370S/N370S	Missense	Type I	No	61.7	0,0	30,6	69,6	754,3	441,7	358,2	714,2	1734,9	1466,0	206,6	5745,5	5776,1	0,0	0,0	0,0	0,0	0,0	0,0	0,0	0,0	0,0	0,0	0,0	0,0	0,0	38,0
		male	N370S/L444P	Missense	Type I	No	46.3	0,0	29,6	52,4	573,0	259,6	378,0	1218,2	597,2	324,1	108,6	3511,1	3540,8	0,0	0,0	0,0	0,0	0,0	0,0	22,0	0,0	0,0	22,0	22,0	0,0	0,0	12,8
		female	N370S/L444P	Missense	Type I	No	69.8	0,0	33,7	28,9	4520,2	1247,6	1155,8	679,1	1890,6	2700,1	186,5	12408,7	12442,3	0,0	0,0	0,0	0,0	0,0	0,0	0,0	0,0	0,0	0,0	0,0	0,0	0,0	61,0
		male	N370S/W378G	Missense	Type I	No	70.7	0,0	254,0	149,6	4207,8	2335,6	1049,2	605,2	1458,9	1384,2	299,8	11490,4	11744,4	0,0	0,0	0,0	0,0	0,0	0,0	0,0	0,0	0,0	0,0	0,0	0,0	0,0	202,9
		female	N370S/N370S	Missense	Type I	No	42.7	0,0	250,5	94,6	2851,3	1118,9	1172,1	1440,5	1467,1	1899,4	476,4	10520,4	10770,9	0,0	0,0	0,0	0,0	0,0	0,0	0,0	0,0	0,0	0,0	0,0	0,0	0,0	75,2
		female	N370S/R496H	Missense	Type I	No	52.4	0,0	66,8	0,0	0,0	0,0	0,0	93,3	0,0	78,0	0,0	171,3	238,1	0,0	0,0	0,0	0,0	0,0	0,0	0,0	0,0	0,0	0,0	0,0	0,0	0,0	146,0
		female	L444P/84GG	Missense/Insertion	Type I	No	44.0	0,0	1071,2	583,7	10080,6	6406,9	6643,7	17208,4	13062,4	5618,4	6654,2	66258,4	67329,6	0,0	0,0	0,0	0,0	0,0	0,0	219,7	0,0	0,0	219,7	219,7	0,0	0,0	43,1
	TG	female	L444P; L444P	Missense	Type I	ERT	9.2	0,0	66,0	0,0	1249,9	354,0	362,4	236,2	347,3	575,3	214,7	3339,9	3405,9	0,0	0,0	0,0	0,0	0,0	0,0	0,0	0,0	0,0	0,0	0,0	0,0	0,0	168,7
		male	N370S; W184R	Missense	Type I	ERT	16.0	0,0	42,3	84,0	581,1	359,8	382,7	1147,6	1008,2	636,7	336,9	4537,0	4579,3	0,0	0,0	0,0	0,0	0,0	0,0	30,5	0,0	0,0	30,5	30,5	0,0	0,0	23,0
		male	N370S; L444P	Missense	Type I	ERT	17.4	0,0	17,7	48,9	320,0	149,5	136,3	281,0	426,2	312,6	53,9	1728,3	1746,1	0,0	0,0	0,0	0,0	0,0	0,0	0,0	0,0	0,0	0,0	0,0	0,0	0,0	36,1
		female	N370S; R496N	Missense	Type I	ERT	16.8	0,0	9,5	0,0	19,4	0,0	17,4	17,8	33,4	17,0	0,0	105,0	114,4	0,0	0,0	0,0	0,0	0,0	0,0	0,0	0,0	0,0	0,0	0,0	0,0	0,0	21,6
		female	L444P; L444P	Missense	Type I	ERT	5.9	0,0	38,4	25,3	472,3	543,1	524,6	1453,7	1034,7	647,2	574,7	5275,6	5314,0	0,0	0,0	0,0	0,0	0,0	0,0	32,0	0,0	0,0	32,0	32,0	0,0	0,0	38,6
MLD	MLD	male	Unknown	Unknown	Adult Form	No	36.0	0,0	0,0	0,0	0,0	0,0	0,0	0,0	0,0	0,0	0,0	0,0	0,0	0,0	0,0	0,0	0,0	0,0	0,0	0,0	0,0	0,0	0,0	0,0	0,0	0,0	254,8
		male	Unknown	Unknown	Late Juvenile	No	8.5	0,0	0,0	0,0	0,0	0,0	0,0	0,0	0,0	0,0	0,0	0,0	0,0	0,0	0,0	0,0	0,0	0,0	0,0	0,0	0,0	0,0	0,0	0,0	0,0	0,0	581,5
		male	Unknown	Unknown	Juvenile	No	23.0	0,0	188,8	0,0	0,0	0,0	0,0	0,0	0,0	0,0	0,0	0,0	188,8	0,0	0,0	0,0	0,0	0,0	0,0	0,0	0,0	0,0	0,0	0,0	0,0	0,0	760,5
		Unknown	Unknown	Unknown	Unknown	No	Unknown	874,6	266,6	0,0	0,0	0,0	0,0	0,0	0,0	0,0	0,0	0,0	266,6	0,0	0,0	0,0	0,0	0,0	0,0	0,0	0,0	0,0	0,0	0,0	0,0	0,0	869,0
Niemann-Pick	NPC	Male	Unknown	Unknown	Type C	No	4.0	0,0	15,8	0,0	34,0	0,0	23,7	0,0	0,0	62,1	0,0	119,8	135,5	0,0	0,0	0,0	0,0	0,0	0,0	0,0	0,0	0,0	0,0	0,0	0,0	0,0	52,6
Gangliosidosis	GM1	Female	Unknown	Unknown	Unknown	yes (unknown)	1.8	0,0	0,0	0,0	49,2	0,0	0,0	0,0	0,0	0,0	0,0	49,2	49,2	0,0	0,0	0,0	0,0	0,0	0,0	0,0	0,0	0,0	0,0	0,0	0,0	0,0	53,3
		Male	Unknown	Unknown	Unknown	yes (unknown)	3.5	0,0	0,0	0,0	0,0	0,0	0,0	0,0	0,0	0,0	0,0	0,0	0,0	0,0	0,0	0,0	0,0	0,0	0,0	0,0	0,0	0,0	0,0	0,0	0,0	0,0	227,8

Table S-2.

Sphingolipidoses (Sph)								Measured levels (pmol/mmol creatinine)																									
Groups	Groups	Gender	Mutation - Variant	Type of mutation	Likely phenotype	Treatment	Age	Lyso-Sulf (+)	GluSph	GluSph (-28)	GluSph (-26)	GluSph (-12)	GluSph (+2)	GluSph (+14)	GluSph (+16)	GluSph (+30)	GluSph (+32)	Analogues	Combined	GalSph	Lyso-Gb3	Lyso-Gb3 (-28)	Lyso-Gb3 (-12)	Lyso-Gb3 (-2)	Lyso-Gb3 (+14)	Lyso-Gb3 (+16)	Lyso-Gb3 (+34)	Lyso-Gb3 (+50)	Analogues	Combined	Lyso-GM2	Lyso-GM1	Lyso-SM
Fabry Disease	UFF	female	A143P	Missence	Classical	No	49,7	0,0	9,0	0,0	0,0	0,0	0,0	0,0	0,0	0,0	0,0	0,0	9,0	0,0	19,0	0,0	97,0	0,0	0,0	82,0	64,0	37,0	280,0	299,0	0,0	0,0	38,0
		female	A348P	Missence	Classical	No	58,7	0,0	7,0	0,0	0,0	0,0	0,0	0,0	0,0	0,0	0,0	0,0	7,0	0,0	88,0	0,0	0,0	0,0	0,0	50,0	89,0	57,0	196,0	284,0	0,0	0,0	36,0
		female	A348P	Missence	Classical	No	55,1	0,0	9,0	0,0	0,0	0,0	0,0	0,0	0,0	0,0	0,0	0,0	9,0	0,0	0,0	0,0	0,0	0,0	0,0	0,0	30,0	0,0	30,0	30,0	0,0	0,0	37,0
		female	A348P	Missence	Classical	No	45,2	0,0	9,0	0,0	0,0	0,0	0,0	0,0	0,0	0,0	0,0	0,0	9,0	0,0	15,0	0,0	0,0	0,0	0,0	0,0	27,0	0,0	27,0	42,0	0,0	0,0	42,0
		female	c.640-2A>G	Intronic	Classical	No	26,3	0,0	24,0	0,0	0,0	0,0	0,0	0,0	0,0	0,0	0,0	0,0	24,0	0,0	67,0	0,0	101,0	0,0	0,0	109,0	76,0	40,0	326,0	393,0	0,0	0,0	66,0
		female	c.802-3_802-2delCA	Deletion	Classical	No	20,3	0,0	15,0	0,0	0,0	0,0	0,0	0,0	0,0	0,0	0,0	0,0	15,0	0,0	19,0	0,0	93,0	0,0	0,0	90,0	98,0	59,0	340,0	359,0	0,0	0,0	46,0
		female	G43V	Missence	Classical	No	37,2	0,0	19,0	0,0	0,0	0,0	0,0	0,0	0,0	0,0	0,0	0,0	19,0	0,0	67,0	0,0	67,0	0,0	0,0	80,0	79,0	37,0	263,0	330,0	0,0	0,0	50,0
		female	L106P (fs*13)	Deletion	Classical	No	38,2	0,0	34,0	0,0	0,0	0,0	0,0	0,0	0,0	0,0	0,0	0,0	34,0	0,0	47,0	0,0	0,0	0,0	0,0	144,0	145,0	0,0	289,0	336,0	0,0	0,0	116,0
		female	L191Q	MIssence	Classical	No	43,9	0,0	0,0	0,0	0,0	0,0	0,0	0,0	0,0	0,0	0,0	0,0	0,0	0,0	0,0	0,0	0,0	0,0	0,0	0,0	0,0	0,0	0,0	0,0	0,0	0,0	266,0
		female	L191Q	Missence	Classical	No	23,0	0,0	54,0	0,0	0,0	0,0	0,0	0,0	0,0	0,0	0,0	0,0	54,0	0,0	24,0	0,0	69,0	0,0	20,0	92,0	71,0	31,0	283,0	307,0	0,0	0,0	69,0
		female	L191Q	Missence	Classical	No	19,9	0,0	45,0	0,0	0,0	0,0	0,0	0,0	0,0	0,0	0,0	0,0	45,0	0,0	86,0	0,0	97,0	0,0	51,0	288,0	382,0	164,0	982,0	1068,0	0,0	0,0	95,0
		female	P293T	Missence	Classical	No	36,5	0,0	8,0	0,0	0,0	0,0	0,0	0,0	0,0	0,0	0,0	0,0	8,0	0,0	14,0	0,0	45,0	0,0	10,0	52,0	40,0	28,0	175,0	189,0	0,0	0,0	22,0
		female	P293T	Missence	Classical	No	40,3	0,0	8,0	0,0	0,0	0,0	0,0	0,0	0,0	0,0	0,0	0,0	8,0	0,0	11,0	0,0	0,0	0,0	0,0	38,0	37,0	33,0	108,0	119,0	0,0	0,0	27,0
		female	Q321E	Missence	Classical	No	20,7	0,0	11,0	0,0	0,0	0,0	0,0	0,0	0,0	0,0	0,0	0,0	11,0	0,0	21,0	0,0	0,0	0,0	0,0	26,0	34,0	27,0	87,0	108,0	0,0	0,0	40,0
		female	Q321E	Missence	Classical	No	62,4	0,0	0,0	0,0	0,0	0,0	0,0	0,0	0,0	0,0	831,0	831,0	831,0	0,0	18,0	0,0	0,0	0,0	0,0	48,0	39,0	0,0	87,0	105,0	0,0	0,0	50,0
		female	Q321R	Missence	Classical	No	48,5	0,0	43,0	0,0	0,0	0,0	0,0	0,0	0,0	0,0	0,0	0,0	43,0	0,0	69,0	0,0	0,0	0,0	0,0	0,0	0,0	0,0	0,0	69,0	0,0	0,0	182,0
		female	R220X	Nonsense	Classical	No	37,1	0,0	11,0	0,0	0,0	0,0	0,0	0,0	0,0	0,0	0,0	0,0	11,0	0,0	29,0	0,0	0,0	0,0	0,0	61,0	42,0	22,0	125,0	154,0	0,0	0,0	36,0
		female	R220X	Nonsense	Classical	No	59,2	0,0	36,0	0,0	0,0	0,0	0,0	0,0	0,0	0,0	0,0	0,0	36,0	0,0	0,0	0,0	0,0	0,0	0,0	0,0	0,0	0,0	0,0	0,0	0,0	0,0	157,0
		female	R227Q	Missence	Classical	No	27,3	0,0	57,0	0,0	0,0	0,0	0,0	0,0	0,0	0,0	0,0	0,0	57,0	0,0	114,0	0,0	0,0	0,0	0,0	0,0	280,0	0,0	280,0	394,0	0,0	0,0	306,0
		female	R332D (fs*16)	Deletion	Classical	No	20,2	0,0	9,0	0,0	0,0	0,0	0,0	0,0	0,0	0,0	0,0	0,0	9,0	0,0	40,0	0,0	0,0	0,0	10,0	50,0	32,0	13,0	105,0	145,0	0,0	0,0	27,0
		female	R332D (fs*16)	Deletion	Classical	No	26,0	0,0	12,0	0,0	0,0	0,0	0,0	0,0	0,0	0,0	0,0	0,0	12,0	0,0	43,0	0,0	0,0	0,0	0,0	34,0	30,0	0,0	64,0	107,0	0,0	0,0	48,0
		female	R342Q	Missence	Classical	No	36,1	0,0	0,0	0,0	0,0	0,0	0,0	0,0	0,0	0,0	0,0	0,0	0,0	0,0	48,0	0,0	0,0	0,0	0,0	77,0	68,0	39,0	184,0	232,0	0,0	0,0	43,0
		female	R342X	Nonsense	Classical	No	22,5	0,0	13,0	0,0	0,0	0,0	0,0	0,0	0,0	0,0	0,0	0,0	13,0	0,0	52,0	0,0	97,0	0,0	32,0	131,0	180,0	57,0	497,0	549,0	0,0	0,0	36,0
		female	S345P	Missence	Classical	No	33,2	0,0	0,0	0,0	0,0	0,0	0,0	0,0	0,0	0,0	0,0	0,0	0,0	0,0	0,0	0,0	0,0	0,0	0,0	0,0	0,0	0,0	0,0	0,0	0,0	0,0	130,0
		female	S401F (fs*?)	Insertion	Classical	No	13,6	0,0	18,0	0,0	0,0	0,0	0,0	0,0	0,0	0,0	0,0	0,0	18,0	0,0	0,0	0,0	0,0	0,0	0,0	0,0	57,0	0,0	57,0	57,0	0,0	0,0	62,0
		female	S401F (fs*?)	Insertion	Classical	No	8,4	0,0	24,0	0,0	0,0	0,0	0,0	0,0	0,0	0,0	0,0	0,0	24,0	0,0	0,0	0,0	0,0	0,0	0,0	114,0	260,0	88,0	462,0	462,0	0,0	0,0	102,0
		female	S401F (fs*?)	Insertion	Classical	No	20,8	0,0	23,0	0,0	0,0	0,0	0,0	0,0	0,0	0,0	0,0	0,0	23,0	0,0	21,0	0,0	0,0	0,0	0,0	61,0	29,0	0,0	90,0	111,0	0,0	0,0	32,0
		Female	c.639+919G>A	Intronic	Later-onset	No	34,4	0,0	0,0	0,0	0,0	0,0	0,0	0,0	0,0	0,0	0,0	0,0	0,0	0,0	0,0	0,0	0,0	0,0	0,0	0,0	0,0	0,0	0,0	0,0	0,0	0,0	756,0
		Female	c.640-801G>A	Missense	Later-onset	No	43,5	0,0	32,0	0,0	0,0	0,0	0,0	0,0	0,0	0,0	0,0	0,0	32,0	0,0	0,0	0,0	0,0	0,0	0,0	0,0	0,0	0,0	0,0	0,0	0,0	0,0	118,0
		Female	c.640-801G>A	Missense	Later-onset	No	11,1	0,0	7,0	0,0	0,0	0,0	0,0	0,0	0,0	0,0	0,0	0,0	7,0	0,0	0,0	0,0	0,0	0,0	0,0	0,0	0,0	0,0	0,0	0,0	0,0	0,0	51,0
		Female	H46P	Intronic	Later-onset	No	52,1	0,0	0,0	0,0	0,0	0,0	0,0	0,0	0,0	0,0	0,0	0,0	0,0	0,0	0,0	0,0	0,0	0,0	0,0	0,0	0,0	0,0	0,0	0,0	0,0	0,0	60,0
		Female	H46P	Missense	Later-onset	No	18,3	0,0	18,0	0,0	0,0	0,0	0,0	0,0	0,0	0,0	0,0	0,0	18,0	0,0	0,0	0,0	0,0	0,0	0,0	0,0	0,0	0,0	0,0	0,0	0,0	0,0	61,0
		Female	N215S	Intronic	Later-onset	No	28,9	0,0	28,0	0,0	0,0	0,0	0,0	0,0	0,0	0,0	0,0	0,0	28,0	0,0	0,0	88,0	0,0	0,0	0,0	0,0	0,0	0,0	88,0	88,0	0,0	0,0	68,0
	TFF	Female	A285D	Missence	Classical	Fabrazyme	47,3	0,0	19,0	0,0	0,0	0,0	0,0	0,0	0,0	0,0	0,0	0,0	19,0	0,0	0,0	0,0	0,0	0,0	0,0	0,0	65,0	0,0	65,0	65,0	0,0	0,0	92,0
		Female	A348P	Missence	Classical	Fabrazyme	45,8	0,0	38,0	0,0	0,0	0,0	0,0	0,0	0,0	0,0	0,0	0,0	38,0	0,0	0,0	0,0	0,0	0,0	0,0	0,0	0,0	0,0	0,0	0,0	0,0	0,0	162,0
		Female	A348P	Missence	Classical	Replagal	53,5	0,0	10,0	0,0	0,0	0,0	0,0	0,0	0,0	0,0	0,0	0,0	10,0	0,0	63,0	0,0	0,0	0,0	0,0	123,0	70,0	0,0	193,0	256,0	0,0	0,0	40,0
		Female	A348P	Missence	Classical	Replagal	50,6	0,0	21,0	0,0	0,0	0,0	0,0	0,0	0,0	0,0	0,0	0,0	21,0	0,0	0,0	0,0	0,0	0,0	0,0	0,0	0,0	0,0	0,0	0,0	0,0	0,0	71,0
		Female	D101F (fs*21)	Deletion	Classical	Fabrazyme	50,8	0,0	41,0	0,0	0,0	0,0	0,0	0,0	0,0	0,0	0,0	0,0	41,0	0,0	32,0	0,0	0,0	0,0	0,0	128,0	155,0	145,0	428,0	460,0	0,0	0,0	88,0
		Female	E338K	Missence	Classical	Replagal	44,4	0,0	0,0	0,0	0,0	0,0	0,0	0,0	0,0	0,0	0,0	0,0	0,0	0,0	0,0	0,0	0,0	0,0	0,0	0,0	49,0	0,0	49,0	49,0	0,0	0,0	82,0
		Female	G43V	Missence	Classical	Fabrazyme	63,3	0,0	0,0	0,0	0,0	0,0	0,0	0,0	0,0	0,0	0,0	0,0	0,0	0,0	0,0	0,0	0,0	0,0	0,0	47,0	49,0	0,0	96,0	96,0	0,0	0,0	70,0
		Female	L414S	Missence	Classical	Fabrazyme	44,4	0,0	6,0	0,0	0,0	0,0	0,0	0,0	0,0	0,0	0,0	0,0	6,0	0,0	0,0	0,0	90,0	0,0	47,0	279,0	77,0	33,0	526,0	526,0	0,0	0,0	17,0
		Female	L414S	Missence	Classical	Galafold	38,5	0,0	6,0	0,0	0,0	0,0	0,0	0,0	0,0	0,0	0,0	0,0	6,0	0,0	56,0	0,0	91,0	0,0	19,0	126,0	126,0	49,0	411,0	467,0	0,0	0,0	30,0
		Female	L414S	Missence	Classical	Replagal	61,4	0,0	37,0	0,0	0,0	0,0	0,0	0,0	0,0	0,0	0,0	0,0	37,0	0,0	22,0	0,0	138,0	0,0	42,0	147,0	91,0	59,0	477,0	499,0	0,0	0,0	80,0
		Female	M187V (fs*6)	Deletion	Classical	Fabrazyme	53,0	0,0	12,0	0,0	0,0	0,0	0,0	0,0	0,0	0,0	0,0	0,0	12,0	0,0	27,0	0,0	88,0	0,0	0,0	118,0	105,0	64,0	375,0	402,0	0,0	0,0	45,0
		Female	c.640-2A>G	Intronic	Classical	Replagal	61,2	0,0	8,0	0,0	0,0	0,0	0,0	0,0	0,0	0,0	0,0	0,0	8,0	0,0	26,0	0,0	89,0	0,0	29,0	152,0	91,0	55,0	416,0	442,0	0,0	0,0	34,0
		Female	P293T	Missence	Classical	Galafold	55,5	0,0	16,0	11,0	0,0	0,0	0,0	0,0	0,0	0,0	0,0	11,0	27,0	0,0	38,0	0,0	0,0	0,0	0,0	51,0	156,0	77,0	284,0	322,0	0,0	0,0	71,0
		Female	P293T	Missence	Classical	Galafold	63,0	0,0	10,0	0,0	0,0	0,0	0,0	0,0	0,0	0,0	0,0	0,0	10,0	0,0	24,0	0,0	63,0	0,0	0,0	69,0	101,0	41,0	274,0	298,0	0,0	0,0	41,0
		Female	Q321R	Missence	Classical	Replagal	56,4	0,0	25,0	0,0	0,0	0,0	0,0	0,0	0,0	0,0	0,0	0,0	25,0	0,0	49,0	0,0	163,0	0,0	88,0	331,0	154,0	109,0	845,0	894,0	0,0	0,0	82,0
		Female	R220X	Nonsense	Classical	Replagal	61,5	0,0	33,0	0,0	0,0	0,0	0,0	0,0	0,0	0,0	0,0	0,0	33,0	0,0	0,0	0,0	0,0	0,0	0,0	0,0	0,0	0,0	0,0	0,0	0,0	0,0	129,0
		Female	S345P	Missence	Classical	Replagal	62,4	0,0	81,0	0,0	0,0	0,0	0,0	0,0	0,0	0,0	0,0	0,0	81,0	0,0	0,0	0,0	0,0	0,0	0,0	0,0	140,0	0,0	140,0	140,0	0,0	0,0	304,0
		Female	S345P	Missence	Classical	Galafold	59,2	0,0	0,0	0,0	0,0	0,0	0,0	0,0	0,0	0,0	0,0	0,0	0,0	0,0	0,0	0,0	0,0	0,0	0,0	0,0	79,0	0,0	79,0	79,0	0,0	0,0	132,0
		Female	V254del	Deletion	Classical	Galafold	61,1	0,0	13,0	0,0	0,0	0,0	0,0	0,0	0,0	0,0	0,0	0,0	13,0	0,0	31,0	0,0	0,0	0,0	0,0	48,0	75,0	74,0	197,0	228,0	0,0	0,0	55,0
		Female	W81S	Missence	Classical	Replagal	71,4	0,0	7,0	0,0	0,0	0,0	0,0	0,0	0,0	0,0	0,0	0,0	7,0	0,0	20,0	0,0	72,0	0,0	29,0	128,0	90,0	53,0	372,0	392,0	0,0	0,0	36,0
		Female	Y134S	Missence	Classical	Replagal	58,1	0,0	20,0	0,0	0,0	0,0	0,0	0,0	0,0	0,0	0,0	0,0	20,0	0,0	0,0	0,0	0,0	0,0	0,0	58,0	91,0	75,0	224,0	224,0	0,0	0,0	79,0
		Female	P293L	Missence	Later-onset	Fabrazyme	62,0	0,0	26,0	0,0	0,0	0,0	0,0	0,0	0,0	0,0	0,0	0,0	26,0	0,0	0,0	0,0	0,0	0,0	0,0	78,0	0,0	0,0	78,0	78,0	0,0	0,0	99,0
		Female	c.639+919G>A	Intronic	Later-onset	Replagal	63,2	0,0	0,0	0,0	0,0	0,0	0,0	0,0	0,0	0,0	0,0	0,0	0,0	0,0	0,0	0,0	0,0	0,0	0,0	0,0	0,0	0,0	0,0	0,0	0,0	0,0	85,0
	UFM	Male	S345P	Missence	Classical	No	14,7	0,0	0,0	0,0	0,0	0,0	0,0	0,0	0,0	0,0	0,0	0,0	0,0	0,0	0,0	0,0	379,0	0,0	107,0	468,0	1033,0	2251,0	4238,0	4238,0	0,0	0,0	126,0
		Male	R342Q	Missence	Classical	No	15,2	0,0	0,0	0,0	0,0	0,0	0,0	0,0	0,0	0,0	0,0	0,0	0,0	0,0	101,0	136,0	772,0	178,0	889,0	3744,0	1432,0	3598,0	10749,0	10850,0	0,0	0,0	62,0
		Male	R112C	Missence	Classical	No	15,9	0,0	0,0	0,0	0,0	0,0	0,0	22,0	63,0	40,0	0,0	125,0	125,0	0,0	46,0	23,0	109,0	21,0	114,0	690,0	279,0	177,0	1413,0	1459,0	0,0	0,0	37,0
		Male	W349X	Nonsense	Classical	No	10,5	0,0	0,0	0,0	0,0	0,0	0,0	0,0	0,0	0,0	0,0	0,0	0,0	0,0	54,0	144,0	844,0	0,0	679,0	2247,0	1852,0	3564,0	9330,0	9384,0	0,0	0,0	90,0
		Male	W349X	Nonsense	Classical	No	8,6	0,0	0,0	0,0	0,0	0,0	0,0	0,0	0,0	0,0	0,0	0,0	0,0	0,0	44,0	0,0	593,0	0,0	310,0	1039,0	1065,0	3258,0	6265,0	6309,0	0,0	0,0	138,0
		Male	S345P	Missence	Classical	No	23,9	0,0	0,0	0,0	0,0	0,0	0,0	0,0	0,0	0,0	0,0	0,0	0,0	0,0	0,0	0,0	0,0	0,0	0,0	0,0	157,0	230,0	387,0	387,0	0,0	0,0	162,0
		Male	R112C	Missence	Classical	No	25,8	0,0	0,0	0,0	0,0	0,0	0,0	0,0	0,0	0,0	0,0	0,0	0,0	0,0	0,0	0,0	0,0	0,0	0,0	107,0	120,0	182,0	409,0	409,0	0,0	0,0	148,0
		Male	Q321E	Missence	Classical	No	31,7	0,0	0,0	0,0	0,0	0,0	0,0	0,0	0,0	0,0	0,0	0,0	0,0	0,0	56,0	39,0	134,0	0,0	125,0	627,0	264,0	546,0	1735,0	1791,0	0,0	0,0	46,0
		Male	R220X	Nonsense	Classical	No	40,1	0,0	0,0	0,0	0,0	0,0	0,0	0,0	0,0	0,0	0,0	0,0	0,0	0,0	108,0	0,0	390,0	0,0	210,0	1500,0	1533,0	3531,0	7164,0	7272,0	0,0	0,0	153,0
		Male	Q321X	Nonsense	Classical	No	48,8	0,0	22,0	0,0	0,0	0,0	0,0	0,0	0,0	0,0	0,0	0,0	22,0	0,0	491,0	286,0	2212,0	497,0	1089,0	5048,0	1906,0	3853,0	14891,0	15382,0	0,0	0,0	102,0
		Male	Y134S	Missence	Classical	No	28,1	0,0	12,0	0,0	0,0	0,0	0,0	0,0	0,0	0,0	0,0	0,0	12,0	0,0	364,0	163,0	763,0	144,0	343,0	1364,0	465,0	1198,0	4440,0	4804,0	0,0	0,0	75,0
		Male	c.640-801G>A	Intronic	Later-onset	No	13,9	0,0	0,0	0,0	0,0	0,0	0,0	0,0	0,0	0,0	0,0	0,0	0,0	0,0	0,0	0,0	0,0	0,0	0,0	71,0	51,0	48,0	170,0	170,0	0,0	0,0	43,0
		Male	R356W	Missence	Later-onset	No	19,9	0,0	0,0	0,0	0,0	0,0	0,0	0,0	0,0	0,0	0,0	0,0	0,0	0,0	0,0	0,0	0,0	0,0	0,0	0,0	0,0	0,0	0,0	0,0	0,0	0,0	92,0
		Male	N215S	Missence	Later-onset	No	59,3	0,0	0,0	0,0	0,0	0,0	0,0	0,0	0,0	0,0	0,0	0,0	0,0	0,0	27,0	0,0	0,0	0,0	0,0	81,0	0,0	0,0	81,0	108,0	0,0	0,0	95,0
		Male	N215S	Missence	Later-onset	No	39,7	0,0	0,0	0,0	0,0	0,0	0,0	0,0	0,0	0,0	0,0	0,0	0,0	0,0	0,0	0,0	0,0	0,0	0,0	66,0	42,0	37,0	145,0	145,0	0,0	0,0	50,0
		Male	R356W	Missence	Later-onset	No	32,0	0,0	0,0	0,0	0,0	0,0	0,0	0,0	0,0	0,0	0,0	0,0	0,0	0,0	0,0	0,0	0,0	0,0	0,0	41,0	57,0	0,0	98,0	98,0	0,0	0,0	60,0
		Male	c.639+919G>A	Intronic	Later-onset	No	40,5	0,0	0,0	0,0	0,0	0,0	0,0	0,0	0,0	0,0	0,0	0,0	0,0	0,0	0,0	0,0	0,0	0,0	0,0	0,0	0,0	0,0	0,0	0,0	0,0	0,0	258,0
		Male	c.639+919G>A	Intronic	Later-onset	No	34,6	0,0	0,0	0,0	0,0	0,0	0,0	0,0	0,0	0,0	0,0	0,0	0,0	0,0	0,0	0,0	0,0	0,0	0,0	0,0	0,0	0,0	0,0	0,0	0,0	0,0	187,0
		Male	N215S	Missence	Later-onset	No	66,9	0,0	0,0	0,0	0,0	0,0	0,0	0,0	0,0	0,0	0,0	0,0	0,0	0,0	24,0	0,0	103,0	0,0	21,0	120,0	36,0	26,0	306,0	330,0	0,0	0,0	40,0
	TFM	Male	Q386X	Nonsense	Classical	Replagal	43,4	0,0	0,0	0,0	0,0	0,0	0,0	0,0	0,0	0,0	0,0	0,0	0,0	0,0	33,0	0,0	178,0	0,0	0,0	225,0	143,0	88,0	634,0	667,0	0,0	0,0	97,0
		Male	S345P	Missence	Classical	Replagal	58,5	0,0	0,0	0,0	0,0	0,0	0,0	0,0	0,0	0,0	0,0	0,0	0,0	0,0	0,0	0,0	0,0	0,0	0,0	80,0	150,0	138,0	368,0	368,0	0,0	0,0	104,0
		Male	R227Q	Missence	Classical	Fabrazyme	48,4	0,0	0,0	0,0	0,0	0,0	0,0	0,0	0,0	0,0	0,0	0,0	0,0	0,0	0,0	0,0	0,0	0,0	0,0	165,0	201,0	154,0	520,0	520,0	0,0	0,0	160,0
		Male	L414S	Missence	Classical	Fabrazyme	40,9	0,0	21,0	0,0	0,0	0,0	0,0	0,0	0,0	0,0	0,0	0,0	21,0	0,0	122,0	100,0	467,0	95,0	126,0	768,0	215,0	143,0	1914,0	2036,0	0,0	0,0	106,0
		Male	A348P	Missence	Classical	Fabrazyme	43,0	0,0	26,0	0,0	0,0	0,0	0,0	0,0	0,0	0,0	0,0	0,0	26,0	0,0	93,0	0,0	0,0	0,0	0,0	178,0	0,0	0,0	178,0	271,0	0,0	0,0	127,0
		Male	C12F (fs*105)	Deletion	Classical	Fabrazyme	45,3	0,0	15,0	0,0	0,0	0,0	0,0	0,0	0,0	0,0	0,0	0,0	15,0	0,0	0,0	0,0	120,0	0,0	41,0	211,0	99,0	73,0	544,0	544,0	0,0	0,0	67,0
		Male	W91S	Missence	Classical	Replagal	53,5	0,0	12,0	0,0	0,0	0,0	0,0	0,0	0,0	0,0	0,0	0,0	12,0	0,0	0,0	0,0	188,0	0,0	53,0	363,0	318,0	313,0	1235,0	1235,0	0,0	0,0	69,0
		Male	R342X	Nonsense	Classical	Replagal	45,0	0,0	0,0	0,0	0,0	0,0	0,0	0,0	0,0	0,0	0,0	0,0	0,0	0,0	136,0	0,0	187,0	0,0	85,0	338,0	179,0	139,0	928,0	1064,0	0,0	0,0	128,0
		Male	A348P	Missence	Classical	Fabrazyme	28,4	0,0	12,0	0,0	0,0	0,0	0,0	0,0	0,0	0,0	0,0	0,0	12,0	0,0	104,0	58,0	377,0	42,0	191,0	1124,0	795,0	939,0	3526,0	3630,0	0,0	0,0	59,0
		Male	M1T	Missence	Classical	Fabrazyme	60,1	0,0	23,0	0,0	0,0	0,0	0,0	0,0	36,0	0,0	0,0	36,0	59,0	0,0	233,0	79,0	554,0	142,0	479,0	2048,0	475,0	341,0	4118,0	4351,0	0,0	0,0	70,0
		Male	A348P	Missence	Classical	Fabrazyme	26,6	0,0	10,0	0,0	0,0	0,0	0,0	0,0	0,0	0,0	0,0	0,0	10,0	0,0	26,0	0,0	0,0	0,0	0,0	76,0	67,0	51,0	194,0	220,0	0,0	0,0	55,0
		Male	A348P	Missence	Classical	Fabrazyme	25,1	0,0	9,0	0,0	0,0	0,0	0,0	0,0	0,0	0,0	0,0	0,0	9,0	0,0	0,0	0,0	58,0	0,0	19,0	85,0	38,0	28,0	228,0	228,0	0,0	0,0	41,0
		Male	A348P	Missence	Classical	Fabrazyme	26,4	0,0	7,0	0,0	0,0	0,0	0,0	0,0	23,0	18,0	0,0	41,0	48,0	0,0	9,0	0,0	0,0	0,0	17,0	101,0	38,0	21,0	177,0	186,0	0,0	0,0	34,0
		Male	M290T	Missence	Classical	Galafold	54,8	0,0	14,0	0,0	0,0	0,0	0,0	30,0	0,0	0,0	0,0	30,0	44,0	0,0	73,0	81,0	352,0	0,0	90,0	566,0	230,0	214,0	1533,0	1606,0	0,0	0,0	66,0
		Male	Q321R	Missence	Classical	Fabrazyme	50,7	0,0	9,0	0,0	0,0	0,0	0,0	0,0	0,0	0,0	0,0	0,0	9,0	0,0	21,0	0,0	123,0	0,0	30,0	131,0	133,0	62,0	479,0	500,0	0,0	0,0	49,0
		Male	A143P	Missence	Classical	Replagal	54,0	0,0	0,0	0,0	0,0	0,0	0,0	0,0	0,0	0,0	0,0	0,0	0,0	0,0	0,0	0,0	0,0	0,0	0,0	153,0	208,0	149,0	510,0	510,0	0,0	0,0	109,0
		Male	L191Q	Missence	Classical	Replagal	52,1	0,0	16,0	0,0	0,0	0,0	0,0	0,0	0,0	0,0	0,0	0,0	16,0	0,0	200,0	71,0	336,0	140,0	172,0	787,0	252,0	161,0	1919,0	2119,0	0,0	0,0	81,0
		Male	P293L	Missence	Classical	Replagal	39,7	0,0	0,0	0,0	0,0	0,0	0,0	0,0	0,0	0,0	0,0	0,0	0,0	0,0	109,0	0,0	228,0	0,0	0,0	347,0	0,0	267,0	842,0	951,0	0,0	0,0	211,0
		Male	A348P	Missence	Classical	Fabrazyme	44,6	0,0	8,0	0,0	0,0	0,0	0,0	0,0	0,0	0,0	0,0	0,0	8,0	0,0	24,0	0,0	83,0	0,0	44,0	289,0	158,0	103,0	677,0	701,0	0,0	0,0	46,0
		Male	R220X	Nonsense	Classical	Fabrazyme	27,6	0,0	21,0	0,0	0,0	0,0	0,0	0,0	0,0	0,0	0,0	0,0	21,0	0,0	37,0	0,0	0,0	0,0	0,0	214,0	374,0	168,0	756,0	793,0	0,0	0,0	104,0
		Male	N215S	Missence	Later-onset	Galafold	41,3	0,0	5,0	0,0	0,0	0,0	0,0	0,0	0,0	0,0	0,0	0,0	5,0	0,0	12,0	0,0	0,0	0,0	0,0	35,0	15,0	0,0	50,0	62,0	0,0	0,0	26,0
		Male	N215S	Missence	Later-onset	Galafold	66,4	0,0	9,0	0,0	0,0	0,0	0,0	0,0	0,0	0,0	0,0	0,0	9,0	0,0	0,0	0,0	0,0	0,0	0,0	26,0	29,0	0,0	55,0	55,0	0,0	0,0	42,0
Gaucher Disease	UG	female	N370S/N370S	Missense	Type I	No	48.3	0,0	31,5	17,4	2529,4	1001,8	936,8	421,3	1123,4	2413,4	133,0	8576,6	8608,1	0,0	0,0	0,0	0,0	0,0	0,0	0,0	0,0	0,0	0,0	0,0	0,0	0,0	43,0
		female	N370S/N370S	Missense	Type I	No	61.7	0,0	30,6	69,6	754,3	441,7	358,2	714,2	1734,9	1466,0	206,6	5745,5	5776,1	0,0	0,0	0,0	0,0	0,0	0,0	0,0	0,0	0,0	0,0	0,0	0,0	0,0	38,0
		male	N370S/L444P	Missense	Type I	No	46.3	0,0	29,6	52,4	573,0	259,6	378,0	1218,2	597,2	324,1	108,6	3511,1	3540,8	0,0	0,0	0,0	0,0	0,0	0,0	22,0	0,0	0,0	22,0	22,0	0,0	0,0	12,8
		female	N370S/L444P	Missense	Type I	No	69.8	0,0	33,7	28,9	4520,2	1247,6	1155,8	679,1	1890,6	2700,1	186,5	12408,7	12442,3	0,0	0,0	0,0	0,0	0,0	0,0	0,0	0,0	0,0	0,0	0,0	0,0	0,0	61,0
		male	N370S/W378G	Missense	Type I	No	70.7	0,0	254,0	149,6	4207,8	2335,6	1049,2	605,2	1458,9	1384,2	299,8	11490,4	11744,4	0,0	0,0	0,0	0,0	0,0	0,0	0,0	0,0	0,0	0,0	0,0	0,0	0,0	202,9
		female	N370S/N370S	Missense	Type I	No	42.7	0,0	250,5	94,6	2851,3	1118,9	1172,1	1440,5	1467,1	1899,4	476,4	10520,4	10770,9	0,0	0,0	0,0	0,0	0,0	0,0	0,0	0,0	0,0	0,0	0,0	0,0	0,0	75,2
		female	N370S/R496H	Missense	Type I	No	52.4	0,0	66,8	0,0	0,0	0,0	0,0	93,3	0,0	78,0	0,0	171,3	238,1	0,0	0,0	0,0	0,0	0,0	0,0	0,0	0,0	0,0	0,0	0,0	0,0	0,0	146,0
		female	L444P/84GG	Missense/Insertion	Type I	No	44.0	0,0	1071,2	583,7	10080,6	6406,9	6643,7	17208,4	13062,4	5618,4	6654,2	66258,4	67329,6	0,0	0,0	0,0	0,0	0,0	0,0	219,7	0,0	0,0	219,7	219,7	0,0	0,0	43,1
	TG	female	L444P; L444P	Missense	Type I	ERT	9.2	0,0	66,0	0,0	1249,9	354,0	362,4	236,2	347,3	575,3	214,7	3339,9	3405,9	0,0	0,0	0,0	0,0	0,0	0,0	0,0	0,0	0,0	0,0	0,0	0,0	0,0	168,7
		male	N370S; W184R	Missense	Type I	ERT	16.0	0,0	42,3	84,0	581,1	359,8	382,7	1147,6	1008,2	636,7	336,9	4537,0	4579,3	0,0	0,0	0,0	0,0	0,0	0,0	30,5	0,0	0,0	30,5	30,5	0,0	0,0	23,0
		male	N370S; L444P	Missense	Type I	ERT	17.4	0,0	17,7	48,9	320,0	149,5	136,3	281,0	426,2	312,6	53,9	1728,3	1746,1	0,0	0,0	0,0	0,0	0,0	0,0	0,0	0,0	0,0	0,0	0,0	0,0	0,0	36,1
		female	N370S; R496N	Missense	Type I	ERT	16.8	0,0	9,5	0,0	19,4	0,0	17,4	17,8	33,4	17,0	0,0	105,0	114,4	0,0	0,0	0,0	0,0	0,0	0,0	0,0	0,0	0,0	0,0	0,0	0,0	0,0	21,6
		female	L444P; L444P	Missense	Type I	ERT	5.9	0,0	38,4	25,3	472,3	543,1	524,6	1453,7	1034,7	647,2	574,7	5275,6	5314,0	0,0	0,0	0,0	0,0	0,0	0,0	32,0	0,0	0,0	32,0	32,0	0,0	0,0	38,6
MLD	MLD	male	Unknown	Unknown	Adult Form	No	36.0	0,0	0,0	0,0	0,0	0,0	0,0	0,0	0,0	0,0	0,0	0,0	0,0	0,0	0,0	0,0	0,0	0,0	0,0	0,0	0,0	0,0	0,0	0,0	0,0	0,0	254,8
		male	Unknown	Unknown	Late Juvenile	No	8.5	0,0	0,0	0,0	0,0	0,0	0,0	0,0	0,0	0,0	0,0	0,0	0,0	0,0	0,0	0,0	0,0	0,0	0,0	0,0	0,0	0,0	0,0	0,0	0,0	0,0	581,5
		male	Unknown	Unknown	Juvenile	No	23.0	0,0	188,8	0,0	0,0	0,0	0,0	0,0	0,0	0,0	0,0	0,0	188,8	0,0	0,0	0,0	0,0	0,0	0,0	0,0	0,0	0,0	0,0	0,0	0,0	0,0	760,5
		Unknown	Unknown	Unknown	Unknown	No	Unknown	874,6	266,6	0,0	0,0	0,0	0,0	0,0	0,0	0,0	0,0	0,0	266,6	0,0	0,0	0,0	0,0	0,0	0,0	0,0	0,0	0,0	0,0	0,0	0,0	0,0	869,0
Niemann-Pick	NPC	Male	Unknown	Unknown	Type C	No	4.0	0,0	15,8	0,0	34,0	0,0	23,7	0,0	0,0	62,1	0,0	119,8	135,5	0,0	0,0	0,0	0,0	0,0	0,0	0,0	0,0	0,0	0,0	0,0	0,0	0,0	52,6
Gangliosidosis	GM1	Female	Unknown	Unknown	Unknown	yes (unknown)	1.8	0,0	0,0	0,0	49,2	0,0	0,0	0,0	0,0	0,0	0,0	49,2	49,2	0,0	0,0	0,0	0,0	0,0	0,0	0,0	0,0	0,0	0,0	0,0	0,0	0,0	53,3
		Male	Unknown	Unknown	Unknown	yes (unknown)	3.5	0,0	0,0	0,0	0,0	0,0	0,0	0,0	0,0	0,0	0,0	0,0	0,0	0,0	0,0	0,0	0,0	0,0	0,0	0,0	0,0	0,0	0,0	0,0	0,0	0,0	227,8

Table S-3

A) Working solution	Calibrators								U-QCs				S-QCs			
**Creatinine (mmol/L) H_2O**	**1**	**2**	**3**	**4**	**5**	**6**	**7**	**8**	**LLOQC**	**LQC**	**MQC**	**HQC**	**LLOQC**	**LQC**	**MQC**	**HQC**
Creatinine	5	10	20	50	75	100	150	200	N/A	N/A	N/A	N/A	5	15	70	150
**Multiplex lysospingolipids (nmol/L) 80:20 MeOH:DMSO**	**1**	**2**	**3**	**4**	**5**	**6**	**7**	**8**	**LLOQ**	**LQC**	**MQC**	**HQC**	**LLOQ**	**LQC**	**MQC**	**HQC**
Lyso-Sulf	7.5	20	40	70	100	140	190	250	7.5	30	100	200	7.5	30	100	200
GluSph	7.5	20	40	70	100	140	190	250	7.5	30	100	200	7.5	30	100	200
GalSph	7.5	20	40	70	100	140	190	250	7.5	30	100	200	7.5	30	100	200
Lyso-Gb_3	7.5	20	40	70	100	140	190	250	7.5	30	100	200	7.5	30	100	200
Lyso-GM1	80	160	320	560	800	1120	1520	2000	80	240	800	1600	80	240	800	1600
Lyso-GM2	30	80	160	280	400	560	760	1000	30	120	400	800	30	120	400	800
Lyso-SM	7.5	20	40	70	100	140	190	250	7.5	30	100	200	7.5	30	100	200
**B) Final concentration on filter paper**	**Calibrators**								**U-QCs**				**S-QCs**			
**Creatinine (mmol/L) H_2O**	**1**	**2**	**3**	**4**	**5**	**6**	**7**	**8**	**LLOQC**	**LQC**	**MQC**	**HQC**	**LLOQC**	**LQC**	**MQC**	**HQC**
Creatinine	0.5	1	2	5	7.5	10	15	20	0.5	1.5	7.0	15.0	0.5	1.5	7.0	15.0
**Multiplex lysospingolipids (nmol/L) 80:20 MeOH:DMSO**	**1**	**2**	**3**	**4**	**5**	**6**	**7**	**8**	**LLOQC**	**LQC**	**MQC**	**HQC**	**LLOQC**	**LQC**	**MQC**	**HQC**
Lyso-Sulf	0.75	2	4	7	10	14	19	25	0.75	3.0	10.0	20.0	0.75	3	10	20
GluSph	0.75	2	4	7	10	14	19	25	0.75	3.0	10.0	20.0	0.75	3	10	20
GalSph	0.75	2	4	7	10	14	19	25	0.75	3.0	10.0	20.0	0.75	3	10	20
Lyso-Gb_3	0.75	2	4	7	10	14	19	25	0.75	3.0	10.0	20.0	0.75	3	10	20
Lyso-GM1	8	16	32	56	80	112	152	200	8	24	80	160	8	24	80	160
Lyso-GM2	3	8	16	28	40	56	76	100	3	12	40	80	3	12	40	80
Lyso-SM	0.75	2	4	7	10	14	19	25	0.75	3.0	10.0	20.0	0.75	3	10	20

U-: Urine; S-: Spiked; QCs: Quality controls; LLOQC: Lower limit of quantification; LQC: Low concentration; MQC: Medium concentration; HQC: High concentration

Table S-4.

Compounds	Precision (CV%)				Accuracy (Bias %)			
**Intraday 1 (n=5)**	**LLOQ**	**LQC**	**MQC**	**HQC**	**LLOQ**	**LQC**	**MQC**	**HQC**
Lyso-Sulfatide	5,4%	6,5%	7,3%	5,6%	1,9%	7,6%	10,0%	3,7%
GluSph	3,1%	7,7%	7,1%	2,5%	2,1%	8,4%	10,3%	3,4%
GalSph	5,7%	6,1%	6,2%	2,6%	0,5%	3,9%	1,9%	-1,9%
Lyso-Gb_3	5,0%	9,8%	5,2%	3,5%	14,7%	18,1%	15,3%	11,8%
Lyso-GM2	12,1%	10,4%	9,2%	6,2%	14,0%	7,7%	9,9%	2,0%
Lyso-GM1	5,2%	12,4%	11,4%	7,0%	7,3%	8,6%	7,6%	8,6%
Lyso-SM	2,5%	6,7%	5,2%	0,9%	-9,8%	52,1%	71,2%	47,8%
Creatinine	4,4%	4,9%	6,2%	4,0%	-15,5%	1,1%	-0,2%	-3,2%
**Intraday 2 (n=5)**	**LLOQ**	**LQC**	**MQC**	**HQC**	**LLOQ**	**LQC**	**MQC**	**HQC**
Lyso-Sulfatide	5,1%	8,0%	3,3%	1,8%	9,6%	1,9%	1,7%	-1,1%
GluSph	5,7%	6,3%	2,8%	3,3%	5,1%	4,1%	4,3%	0,2%
GalSph	4,4%	5,6%	2,0%	3,5%	5,2%	0,6%	-0,3%	-3,3%
Lyso-Gb_3	8,8%	3,4%	3,0%	4,2%	19,7%	8,0%	5,9%	2,5%
Lyso-GM2	6,5%	8,6%	7,3%	4,0%	23,6%	-1,7%	0,5%	-8,8%
Lyso-GM1	9,1%	11,2%	7,3%	4,2%	4,0%	-3,2%	-1,3%	-10,1%
Lyso-SM	2,2%	4,6%	2,4%	2,8%	4,1%	36,0%	51,3%	32,2%
Creatinine	2,7%	7,4%	3,8%	5,5%	-10,1%	6,7%	-2,6%	-3,1%
**Intraday 3 (n=5)**	**LLOQ**	**LQC**	**MQC**	**HQC**	**LLOQ**	**LQC**	**MQC**	**HQC**
Lyso-Sulfatide	7,6%	5,8%	5,1%	5,1%	7,4%	13,2%	13,2%	3,9%
GluSph	10,3%	4,2%	3,7%	4,4%	6,0%	9,9%	8,9%	1,8%
GalSph	5,8%	4,4%	3,1%	4,5%	4,3%	4,9%	2,4%	-3,7%
Lyso-Gb_3	8,4%	4,7%	5,7%	4,9%	19,0%	14,3%	14,2%	9,5%
Lyso-GM2	4,4%	5,3%	4,3%	5,5%	6,8%	11,5%	13,8%	3,7%
Lyso-GM1	4,9%	5,4%	4,0%	5,5%	1,7%	7,8%	12,2%	4,7%
Lyso-SM	4,9%	3,5%	3,6%	3,8%	46,1%	44,2%	51,8%	26,5%
Creatinine	5,4%	2,8%	3,3%	5,8%	-5,45%	0,29%	-4,34%	-5,71%
**Interday (n=3)**	**LLOQ**	**LQC**	**MQC**	**HQC**	**LLOQ**	**LQC**	**MQC**	**HQC**
Lyso-Sulfatide	6,5%	7,8%	6,9%	4,9%	6,0%	7,6%	7,9%	2,7%
GluSph	6,8%	6,1%	5,2%	3,5%	4,3%	7,4%	7,2%	2,0%
GalSph	5,3%	5,3%	3,8%	3,4%	2,8%	3,1%	0,8%	-2,7%
Lyso-Gb_3	7,2%	7,0%	5,9%	5,5%	18,3%	13,1%	10,8%	7,9%
Lyso-GM2	10,0%	9,3%	8,4%	7,7%	15,5%	5,7%	6,7%	-1,0%
Lyso-GM1	6,7%	10,5%	9,1%	9,9%	4,1%	4,1%	6,1%	0,9%
Lyso-SM	22,0%	6,5%	6,9%	7,3%	14,9%	43,5%	57,2%	34,7%
Creatinine	6,23%	5,81%	4,70%	4,98%	-9,67%	3,03%	-2,41%	-3,92%
FDA recommandation	≤20%	≤15%	≤15%	≤15%	≤20%	≤15%	≤15%	≤15%

LLOQ: Lower limit of quantitation quality control; LQC: Low concentration quality control; MQC: Medium concentration quality control; HQC: High concentration quality control;

Table S-5.

	Limit		Linearity	
**Biomarkers**	**LOD (n=10)**	**LLOQ (n=10)**	**r (n=15)**	**R^2 (n=15)**
Creatinine (+1)	50 nM	170 nM	0.9987 (0.9979-0.9997)	0.9975 (0.9958-0.9994)
Lyso-Sulf (+)	81 pM	269 pM	0.9987 (0.9979-0.9999)	0.9974 (0.9957-0.9997)
GalSph	48 pM	160 pM	0.9988 (0.9973-0.9996)	0.9976 (0.9947-0.9993)
GluSph	15 pM	49 pM	0.9988 (0.9981-0.9993)	0.9979 (0.9964-0.9996)
GluSph (-28)	6 pM	21 pM		
GluSph (-26)	31 pM	103 pM		
GluSph (-12)	33 pM	111 pM		
GluSph (+2)	36 pM	120 pM		
GluSph (+14)	27 pM	91 pM		
GluSph (+16)	28 pM	94 pM		
GluSph (+30)	21 pM	70 pM		
GluSph (+32)	19 pM	65 pM		
LysoGb$_3$	34 pM	115 pM	0.9983 (0.9975-0.9995)	0.9967 (0.9951-0.9989)
LysoGb$_3$ (-28)	92 pM	306 pM		
LysoGb$_3$ (-12)	164 pM	547 pM		
LysoGb$_3$ (-2)	89 pM	297 pM		
LysoGb$_3$ (+14)	58 pM	194 pM		
LysoGb$_3$ (+16)	69 pM	230 pM		
LysoGb$_3$ (+34)	62 pM	207 pM		
LysoGb$_3$ (+50)	87 pM	289 pM		
LysoGM2	481 pM	1602 pM	0.9982 (0.9972-0.9992)	0.9965 (0.9951-0.9985)
Lyso-GM1	1447 pM	4823 pM	0.9982 (0.9968-0.9993)	0.9967 (0.9936-0.9996)
LysoSM	60 pM	210 pM	0.9982 (0.9977-0.9992)	0.9964 (0.9954-0.9983)
FDA recommandation	N/A	N/A	N/A	≥0,9950
LOD: Limit of detection; LLOQ: Lower limit of quantification; r: Pearson correlation; R^2 : Coefficient of determination				

Table S-6.

Extraction Recovery (%)									
Spiked (n=3)	Creatinine index	Creatinine	Lyso-Sulf	Glusph	Galsph	Lyso-Gb_3	Lyso-GM2	Lyso-GM1	Lyso-SM
	1.5 mmol/l	90,1%	66,3%	81,8%	79,3%	65,8%	81,4%	86,5%	70,1%
	7.0 mmol/l	70,2%	77,2%	85,3%	84,1%	71,5%	81,0%	89,0%	74,3%
L	15 mmol/l	N/A	77,2%	87,9%	87,4%	81,1%	79,3%	72,6%	75,9%
	1.5 mmol/l	90,7%	74,5%	88,4%	88,5%	74,3%	88,0%	88,0%	81,6%
	7.0 mmol/l	82,5%	85,3%	91,9%	88,0%	85,5%	91,7%	85,6%	87,1%
M	15 mmol/l	N/A	87,8%	89,6%	88,1%	84,3%	84,3%	82,7%	87,2%
	1.5 mmol/l	87,1%	69,1%	88,3%	87,5%	75,1%	88,1%	86,1%	84,2%
	7.0 mmol/l	63,5%	81,1%	85,6%	82,1%	87,1%	78,4%	76,6%	81,9%
H	15 mmol/l	N/A	83,8%	86,4%	84,8%	92,5%	80,6%	78,4%	86,1%
**Matrix Effect (%)**									
Spiked (n=3)	Creatinine index	Creatinine	Lyso-Sulf	Glusph	Galsph	Lyso-Gb_3	Lyso-GM2	Lyso-GM1	Lyso-SM
	1.5 mmol/l	8,7%	8,7%	2,2%	-7,1%	10,2%	19,8%	28,9%	-2,4%
	7.0 mmol/l	-3,1%	14,5%	0,8%	-10,7%	25,1%	25,7%	44,2%	-6,1%
L	15 mmol/l	N/A	13,8%	2,6%	-4,0%	21,6%	29,8%	48,2%	-3,3%
	1.5 mmol/l	0,8%	11,2%	2,7%	-3,6%	10,4%	14,1%	20,7%	-0,9%
	7.0 mmol/l	4,9%	16,1%	4,2%	-7,3%	28,4%	27,6%	45,2%	0,0%
M	15 mmol/l	N/A	15,1%	2,8%	-4,9%	21,7%	29,5%	51,2%	-0,3%
	1.5 mmol/l	1,6%	10,9%	2,9%	-4,9%	19,9%	17,4%	23,5%	5,0%
	7.0 mmol/l	1,2%	16,5%	1,2%	-12,8%	37,2%	26,6%	43,4%	1,9%
H	15 mmol/l	N/A	17,0%	1,8%	-9,6%	34,1%	33,9%	52,5%	4,1%
L: Low concentration; M: Medium concentration; H: High concentration; N/A: not available									

Table S-7.

			Temperature											
			Autosampler (10°C)		35°C		21.5°C		4°C		-30°C		-80°C	
Compound	QC levels	Stability (Days)	BN% (n=5)	CV (%)	BN% (n=5)	CV (%)	BN% (n=5)	CV (%)	BN% (n=5)	CV (%)	BN% (n=5)	CV (%)	BN% (n=5)	CV (%)
Lyso-Sulfatide	U-LQC	0	0	5.8	0,0	3,0	0,0	12,0	0,0	4,0	0,0	8.3	0,0	3,0
		1	1.1	7.2	N/A	N/A	N/A	N/A	N/A	N/A	N/A	N/A	N/A	N/A
		3	N/A	N/A	-10,0	10.2	5.4	10.3	-0.6	6.8	11.4	11.5	8.3	2.2
		6	N/A	N/A	-17.7	20.3	-7.3	5.4	-6.1	5.3	0.2	9.5	-5.5	13.2
		26	N/A	N/A	-27.8	9,0	-8,0	7.1	-3.4	7.8	6.5	9,0	-6.7	7.9
		117	N/A	N/A	N/A	N/A	N/A	N/A	N/A	N/A	8.8	1.4	2.9	5.8
	U-HQC	0	0	5.1	0,0	5.4	0,0	6.2	0,0	1.7	0,0	10.2	0,0	8,0
		1	1.3	6.6	N/A	N/A	N/A	N/A	N/A	N/A	N/A	N/A	N/A	N/A
		3	N/A	N/A	1.8	1.5	-1.7	5.5	3.1	1.6	6.3	9.3	15.9	14.5
		6	N/A	N/A	-15.2	6.1	-12.2	4,0	-16.8	10.8	-2.5	2.1	-9.1	3.4
		26	N/A	N/A	-6.6	5,0	-0.1	4,0	-3.8	5.3	3.8	7.2	10.5	8.8
		117	N/A	N/A	N/A	N/A	N/A	N/A	N/A	N/A	5.5	5.1	10.7	11.7
Glucosylsphingosine	U-LQC	0	0	4.2	0,0	2.9	0,0	7,0	0,0	4.9	0,0	6.6	0,0	6.4
		1	3.3	3.8	N/A	N/A	N/A	N/A	N/A	N/A	N/A	N/A	N/A	N/A
		3	N/A	N/A	-15.7	3.7	-5.8	5.5	-1.3	8.6	-3.7	8.4	-4.5	3.8
		6	N/A	N/A	-17,0	6.4	-4.5	3.2	-0.9	4.9	-4.8	6.1	-5.6	7.6
		26	N/A	N/A	-22.8	7.1	-10.1	4.3	-0.9	4.7	0.4	6.4	-11,0	2.5
		117	N/A	N/A	N/A	N/A	N/A	N/A	N/A	N/A	0.8	2.3	-3.1	1,0
	U-HQC	0	0	4.4	0,0	2.5	0,0	5.3	0,0	11.2	0,0	7,0	0,0	4.3
		1	2.7	3.4	N/A	N/A	N/A	N/A	N/A	N/A	N/A	N/A	N/A	N/A
		3	N/A	N/A	-9.3	2.9	-10.9	3.3	-15.6	4.5	-6.1	5,0	-2.8	10.9
		6	N/A	N/A	-6.9	6.7	-4.6	3.7	-12.1	3.5	0.5	2.8	-1.3	0.6
		26	N/A	N/A	-15,0	6.3	-12.3	0.8	-12.5	1.3	-6,0	5.6	-3.2	7.6
		117	N/A	N/A	N/A	N/A	N/A	N/A	N/A	N/A	0.3	4.1	3.3	6.9
Galactosylsphingosine	U-LQC	0	0	4.4	0,0	3.1	0,0	6.9	0,0	6.2	0,0	6.1	0,0	6.4
		1	4.1	3.2	N/A	N/A	N/A	N/A	N/A	N/A	N/A	N/A	N/A	N/A
		3	N/A	N/A	-13.6	4,0	-4.9	4.4	4.3	7.7	-4.6	8.4	-1,0	4.3
		6	N/A	N/A	-12.5	8,0	-2.1	3.5	8.9	3,0	-1.5	6.4	1,0	6,0
		26	N/A	N/A	-21.3	4.2	-7.9	4.2	6.7	5.3	1.1	6.3	-9.5	3.3
		117	N/A	N/A	N/A	N/A	N/A	N/A	N/A	N/A	2.7	3.9	-1,0	2.7
	U-HQC	0	0	4.5	0,0	1.7	0,0	5,0	0,0	9.9	0,0	6.6	0,0	5.2
		1	3.3	3.6	N/A	N/A	N/A	N/A	N/A	N/A	N/A	N/A	N/A	N/A
		3	N/A	N/A	-8.7	3.6	-11.4	3.7	-15.4	4.6	-3.2	4.8	-3.3	10.6
		6	N/A	N/A	2.1	7.1	1.9	4.5	-5.5	1.7	12.5	3.2	4.6	1.1
		26	N/A	N/A	-11.7	6,0	-8.4	0.8	-11.3	1.6	-1.7	5.5	-3.8	6,0
		117	N/A	N/A	N/A	N/A	N/A	N/A	N/A	N/A	5.1	5,0	3.8	6.9
Globotriaosylsphingosine	U-LQC	0	0	4.7	0,0	0.8	0,0	9.1	0,0	5.4	0,0	5.3	0,0	4.9
		1	7.0	3.6	N/A	N/A	N/A	N/A	N/A	N/A	N/A	N/A	N/A	N/A
		3	N/A	N/A	-6.8	3.1	1.6	7.6	11.1	8.3	8.3	9.1	8.6	3.3
		6	N/A	N/A	-6.3	10.4	9,0	4.2	8.9	4.6	5.5	7.6	9.1	7.5
		26	N/A	N/A	-15.4	4.8	-3.1	4,0	12.1	2.2	11.9	10.2	4.4	2.9
		117	N/A	N/A	N/A	N/A	N/A	N/A	N/A	N/A	14.2	6.5	2.4	3.2
	U-HQC	0	0	4.9	0,0	2.3	0,0	5.2	0,0	11.9	0,0	6,0	0,0	7.2
		1	7.3	4.9	N/A	N/A	N/A	N/A	N/A	N/A	N/A	N/A	N/A	N/A
		3	N/A	N/A	3.5	5.3	2.8	3.1	1.1	3.6	11.3	6.2	15.3	13.1
		6	N/A	N/A	12.6	5.7	11.4	4.9	6.1	6,0	19.4	3.4	17.9	1.4
		26	N/A	N/A	-11.7	6.9	-9.7	3.1	-5.5	4.3	3.8	5.4	7.1	5.5
		117	N/A	N/A	N/A	N/A	N/A	N/A	N/A	N/A	7,0	0.5	14.3	1.7
Lyso-GM2	U-LQC	0	0	5.3	0,0	7,0	0,0	9.6	0,0	8.2	0,0	6.1	0,0	15.7
		1	3.3	1.4	N/A	N/A	N/A	N/A	N/A	N/A	N/A	N/A	N/A	N/A
		3	N/A	N/A	-9.9	1.9	9.1	5.7	5.6	9.7	9.6	12.3	13.6	2.1
		6	N/A	N/A	-22.4	8.3	-5.6	3,0	-7.7	4.4	-5.4	7,0	1.2	2,0
		26	N/A	N/A	-19.3	7.7	-7.3	2.3	5.5	5.3	7.2	10.7	7.8	5.2
		117	N/A	N/A	N/A	N/A	N/A	N/A	N/A	N/A	11.4	3.5	14.6	5.8
	U-HQC	0	0	5.5	0,0	6.2	0,0	2.9	0,0	17.4	0,0	7.3	0,0	5.2
		1	1.4	2.7	N/A	N/A	N/A	N/A	N/A	N/A	N/A	N/A	N/A	N/A
		3	N/A	N/A	-4.7	6.5	2.7	5.2	7.9	0.6	12.8	10,0	11.5	17.5
		6	N/A	N/A	-13.5	3.1	-8.2	1.7	-0.7	12.3	0.8	3.7	-3.4	17.6
		26	N/A	N/A	-14.3	4.8	-13.5	6.9	-4.6	3.7	-0.1	7.2	-3.6	4.7
		117	N/A	N/A	N/A	N/A	N/A	N/A	N/A	N/A	4.9	1.3	9.4	2,0
Lyso-GM1	U-LQC	0	0	5.4	0,0	8,0	0,0	8,0	0,0	8,0	0,0	8,0	0,0	8,0
		1	3.0	2,0	N/A	N/A	N/A	N/A	N/A	N/A	N/A	N/A	N/A	N/A
		3	N/A	N/A	1.8	2.5	5.3	9.6	2.3	10.6	13.1	11.8	8.7	6.5
		6	N/A	N/A	-26.4	12.3	-14.9	6.9	-24.1	12.3	-12.3	9.1	-22,0	8.7
		26	N/A	N/A	-8.1	3.1	-8.5	3,0	0.7	6.5	11.7	12.1	0.9	2.6
		117	N/A	N/A	N/A	N/A	N/A	N/A	N/A	N/A	14.8	7.9	18.5	1.9
	U-HQC	0	0	5.5	0,0	6.2	0,0	6.1	0,0	8.5	0,0	8.9	0,0	4.9
		1	3.6	3.3	N/A	N/A	N/A	N/A	N/A	N/A	N/A	N/A	N/A	N/A
		3	N/A	N/A	0.2	8.4	1.3	5.5	4.1	1.7	12.2	10,0	13.6	17.6
		6	N/A	N/A	-15,0	3.8	-8.7	3.3	0.3	22.3	-1,0	6.3	-11.2	11.5
		26	N/A	N/A	-19,0	1.2	-13,0	5.3	-7.6	5.3	-1.8	6.6	-4.2	7.2
		117	N/A	N/A	N/A	N/A	N/A	N/A	N/A	N/A	-5.1	6.6	2.8	1.4
Lyso-SM	U-LQC	0	0	3.5	0,0	1.9	0,0	8.2	0,0	4.8	0,0	9.2	0,0	4.8
		1	1.8	2.3	N/A	N/A	N/A	N/A	N/A	N/A	N/A	N/A	N/A	N/A
		3	N/A	N/A	-13.7	3,0	-0.3	5.4	1.1	6.5	3,0	12.6	-0.5	4.7
		6	N/A	N/A	-21.1	10.6	-2.6	3,0	-1.2	3.7	-6.3	8.8	-5.7	5.9
		26	N/A	N/A	-36.8	7.7	-23.1	4.5	-13.7	6.5	-14.7	9,0	-22.9	4,0
		117	N/A	N/A	N/A	N/A	N/A	N/A	N/A	N/A	-15,0	2.5	-17.3	2.7
	U-HQC	0	0	3.8	0,0	2.7	0,0	5,0	0,0	10.9	0,0	7.6	0,0	5.9
		1	-2.0	4.2	N/A	N/A	N/A	N/A	N/A	N/A	N/A	N/A	N/A	N/A
		3	N/A	N/A	-2.1	4.3	-3.1	3.3	-10.6	4.7	17.5	6.9	2.9	12.6
		6	N/A	N/A	-8,0	6.5	-6.5	2.9	-19.1	4.8	16.1	2.4	-8.3	1.3
		26	N/A	N/A	-24.9	6.3	-22.7	3.4	-26.3	2.1	-2.7	6,0	-14,0	9.1
		117	N/A	N/A	N/A	N/A	N/A	N/A	N/A	N/A	-7,0	3.2	-15.2	7.9
Creatinine	U-LQC	0	0	2.1	0,0	8.7	0,0	8.7	0,0	8.7	0,0	8.7	0,0	8.7
		1	2.3	2.2	N/A	N/A	N/A	N/A	N/A	N/A	N/A	N/A	N/A	N/A
		3	N/A	N/A	-4.7	8.3	-7.9	2.1	-1.8	2.1	-0.4	6.4	-3.9	2.9
		6	N/A	N/A	-1.9	12.7	-4.4	1.5	-2.2	1.9	-1.5	4.7	-1.9	8.2
		26	N/A	N/A	3.2	9.5	-0.9	0.6	4.9	8.7	10.2	5.8	9.4	5.9
		117	N/A	N/A	N/A	N/A	N/A	N/A	N/A	N/A	N/A	N/A	N/A	N/A
	U-HQC	0	0	2.6	0,0	3.3	0,0	3.3	0,0	3.3	0,0	3.3	0,0	3.3
		1	2.7	2.0	N/A	N/A	N/A	N/A	N/A	N/A	N/A	N/A	N/A	N/A
		3	N/A	N/A	0.7	9.4	-5.9	3.6	-3,0	10.5	-7.6	2.9	-6.2	1.4
		6	N/A	N/A	7.4	5.7	2,0	6.7	1.5	2.2	2.8	11.8	-1.4	5.6
		26	N/A	N/A	3.2	7.9	-3.6	3.5	2.3	7,0	0.9	5.2	-1.1	3.7
		117	N/A	N/A	N/A	N/A	N/A	N/A	N/A	N/A	N/A	N/A	N/A	N/A
FDA recommandation			≤15.0%	N/A	≤15.0%	N/A	≤15.0%	N/A	≤15.0%	N/A	≤15.0%	N/A	≤15.0%	N/A

N/A: Not available; BN: Bias nominal (%) CV: Coefficient of variation (%)

Table S-8.

		U-LQC (n=3)			U-HQC (n=3)		
Compounds	FT cycles	0X	3X	5X	0X	3X	5X
Creatinine	BN (%)	0,0%	1,7%	5,1%	0,0%	-5,0%	-2,9%
	CV (%)	10,1%	7,7%	6,2%	6,6%	3,9%	0,6%
Lyso-Sulf	BN (%)	0,0%	4,6%	2,7%	0,0%	2,8%	18,1%
	CV (%)	1,9%	8,1%	6,5%	5,1%	11,9%	4,6%
GluSph	BN (%)	0,0%	3,6%	-0,5%	0,0%	-2,1%	7,2%
	CV (%)	5,0%	4,5%	8,6%	4,2%	5,3%	3,8%
GalSph	BN (%)	0,0%	3,1%	0,4%	0,0%	0,1%	9,0%
	CV (%)	6,1%	3,0%	6,5%	4,7%	5,2%	3,2%
Lyso-Gb_3	BN (%)	0,0%	-1,4%	-0,6%	0,0%	3,2%	12,7%
	CV (%)	2,2%	12,5%	6,9%	4,7%	8,1%	4,0%
Lyso-GM2	BN (%)	0,0%	-5,9%	-1,2%	0,0%	10,7%	18,0%
	CV (%)	8,2%	9,3%	4,0%	6,4%	10,9%	11,5%
Lyso-GM1	BN (%)	0,0%	-6,6%	4,8%	0,0%	22,7%	22,7%
	CV (%)	1,5%	12,5%	6,5%	9,1%	5,9%	11,0%
Lyso-SM	BN (%)	0,0%	2,7%	-2,9%	0,0%	-3,4%	6,8%
	CV (%)	3,6%	3,7%	7,8%	3,7%	5,1%	2,5%
FT: Freeze-thaw; U: urine; QC: Quality control; LQC: Low concentration QC; HQC: High concentration QC; BN: Bias nominal (%); CV: Coefficient of variation (%)							

Table S-9.

Dilution factors		U-MQC (n=5) Disk 5 cm	Half disk (1:2)	Quarter disk (1:4)
Creatinine	Bias (%)	0,0%	4,9%	-1,1%
	CV (%)	7,2%	4,8%	2,3%
Lyso-Sulf	Bias (%)	0,0%	-3,3%	-3,1%
	CV (%)	2,6%	7,9%	5,9%
GluSph	Bias (%)	0,0%	-4,0%	-4,6%
	CV (%)	5,7%	7,2%	8,4%
GalSph	Bias (%)	0,0%	-6,3%	-15,1%
	CV (%)	5,1%	7,3%	8,6%
Lyso-Gb_3	Bias (%)	0,0%	-6,6%	7,4%
	CV (%)	5,5%	6,7%	9,6%
Lyso-GM2	Bias (%)	0,0%	-10,1%	-10,5%
	CV (%)	7,1%	7,9%	9,1%
Lyso-GM1	Bias (%)	0,0%	2,8%	5,7%
	CV (%)	6,1%	7,4%	9,2%
Lyso-SM	Bias (%)	0,0%	-3,3%	-3,3%
	CV (%)	5,4%	7,6%	9,1%

U: urine; QC: Quality control; MQC: Medium concentration QC; CV: Coefficient of variation (%)

Table S-10.

Healthy controls			Urine biomarkers (pmol/mmol creat)																									
Sample	Gender	Age	Lyso-Sulf	GluSph	GluSph (-28)	GluSph (-26)	GluSph (-12)	GluSph (+2)	GluSph (+14)	GluSph (+16)	GluSph (+3nd)	GluSph (+32)	Regrouped GluSph related analogs	GluSph and its related analogs	GalSph	Lyso-Gb3	Lyso-Gb3 (-28)	Lyso-Gb3 (-12)	Lyso-Gb3 (-2)	Lyso-Gb3 (+14)	Lyso-Gb3 (+16)	Lyso-Gb3 (+34)	Lyso-Gb3 (+5nd)	Regrouped Lyso-Gb3 related analogs	Lyso-Gb3 and its related analogs	Lyso-GM2	Lyso-GM1	Lyso-SM
1	female	2,6	nd	nd	nd	nd	nd	nd	nd	nd	nd	nd	nd	nd	nd	nd	nd	nd	nd	nd	nd	nd	nd	nd	nd	nd	nd	290
2	female	7,0	nd	8	nd	nd	nd	nd	nd	nd	nd	nd	nd	8	nd	nd	nd	nd	nd	nd	nd	nd	nd	nd	nd	nd	nd	59
3	female	7,1	nd	10	nd	nd	nd	nd	nd	nd	nd	nd	nd	10	nd	nd	nd	nd	nd	nd	nd	nd	nd	nd	nd	nd	nd	84
4	female	8,6	nd	nd	nd	nd	nd	nd	nd	nd	nd	nd	nd	nd	nd	nd	nd	nd	nd	nd	nd	nd	nd	nd	nd	nd	nd	216
5	female	8,8	nd	7	nd	nd	nd	nd	nd	nd	nd	nd	nd	7	nd	nd	nd	nd	nd	nd	nd	nd	nd	nd	nd	nd	nd	43
6	female	9,3	nd	6	nd	nd	nd	nd	nd	nd	nd	nd	nd	6	nd	nd	nd	nd	nd	nd	nd	nd	nd	nd	nd	nd	nd	36
7	female	10,0	nd	13	nd	nd	nd	nd	nd	nd	nd	nd	nd	13	nd	nd	nd	nd	nd	nd	nd	nd	nd	nd	nd	nd	nd	81
8	female	10,1	nd	7	nd	nd	nd	nd	nd	nd	nd	nd	nd	7	nd	nd	nd	nd	nd	nd	nd	nd	nd	nd	nd	nd	nd	57
9	female	10,4	nd	nd	nd	nd	nd	nd	nd	nd	nd	nd	nd	nd	nd	nd	nd	nd	nd	nd	nd	nd	nd	nd	nd	nd	nd	203
10	female	14,1	nd	37	nd	nd	nd	nd	nd	nd	nd	nd	nd	37	nd	nd	nd	nd	nd	nd	nd	nd	nd	nd	nd	nd	nd	324
11	female	14,6	nd	5	nd	nd	nd	nd	nd	nd	nd	nd	nd	5	nd	nd	nd	nd	nd	nd	nd	nd	nd	nd	nd	nd	nd	33
12	female	15,9	nd	nd	nd	nd	nd	nd	nd	nd	nd	nd	nd	nd	nd	nd	nd	nd	nd	nd	nd	nd	nd	nd	nd	nd	nd	48
13	female	16,2	nd	28	nd	nd	nd	nd	nd	nd	nd	nd	nd	28	nd	nd	nd	nd	nd	nd	nd	nd	nd	nd	nd	nd	nd	121
14	female	16,8	nd	12	nd	nd	nd	nd	nd	nd	nd	nd	nd	12	nd	nd	nd	nd	nd	nd	nd	nd	nd	nd	nd	nd	nd	70
15	female	17,8	nd	12	nd	nd	nd	nd	nd	nd	nd	nd	nd	12	nd	nd	nd	nd	nd	nd	nd	nd	nd	nd	nd	nd	nd	120
16	female	18,1	nd	24	nd	nd	nd	nd	nd	nd	nd	nd	nd	24	nd	nd	nd	nd	nd	nd	nd	nd	nd	nd	nd	nd	nd	165
17	female	23,4	nd	11	nd	nd	nd	nd	nd	nd	nd	nd	nd	11	nd	nd	nd	nd	nd	nd	nd	nd	nd	nd	nd	nd	nd	45
18	female	27,3	nd	8	nd	nd	nd	nd	nd	nd	nd	nd	nd	8	nd	nd	nd	nd	nd	nd	nd	nd	nd	nd	nd	nd	nd	68
19	female	30,1	nd	nd	nd	nd	nd	nd	nd	nd	nd	nd	nd	nd	nd	nd	nd	nd	nd	nd	nd	nd	nd	nd	nd	nd	nd	0
20	female	30,2	nd	nd	nd	nd	nd	nd	nd	nd	nd	nd	nd	nd	nd	nd	nd	nd	nd	nd	nd	nd	nd	nd	nd	nd	nd	193
21	female	30,5	nd	4	nd	nd	nd	nd	nd	nd	nd	nd	nd	4	nd	nd	nd	nd	nd	nd	nd	nd	nd	nd	nd	nd	nd	31
22	female	30,5	nd	9	nd	nd	nd	nd	nd	nd	nd	nd	nd	9	nd	nd	nd	nd	nd	nd	nd	nd	nd	nd	nd	nd	nd	74
23	female	33,5	nd	nd	nd	nd	nd	nd	nd	nd	nd	nd	nd	nd	nd	nd	nd	nd	nd	nd	nd	nd	nd	nd	nd	nd	nd	366
24	female	36,1	nd	11	nd	nd	nd	nd	nd	nd	nd	nd	nd	11	nd	nd	nd	nd	nd	nd	nd	nd	nd	nd	nd	nd	nd	55
25	female	36,5	nd	37	nd	nd	nd	nd	nd	nd	nd	nd	nd	37	nd	nd	nd	nd	nd	nd	nd	nd	nd	nd	nd	nd	nd	182
26	female	37,6	nd	13	nd	nd	nd	nd	nd	nd	nd	nd	nd	13	nd	nd	nd	nd	nd	nd	nd	nd	nd	nd	nd	nd	nd	66
27	female	37,8	nd	10	nd	nd	nd	nd	nd	nd	nd	nd	nd	10	nd	nd	nd	nd	nd	nd	nd	nd	nd	nd	nd	nd	nd	66
28	female	40,1	nd	nd	nd	nd	nd	nd	nd	nd	nd	nd	nd	nd	nd	25	nd	nd	nd	nd	nd	nd	nd	nd	25	nd	nd	108
29	female	41,6	nd	47	nd	nd	nd	nd	nd	nd	nd	nd	nd	47	nd	nd	nd	nd	nd	nd	nd	nd	nd	nd	nd	nd	nd	109
30	female	42,7	nd	53	nd	nd	nd	nd	nd	nd	nd	nd	nd	53	nd	nd	nd	nd	nd	nd	nd	nd	nd	nd	nd	nd	nd	492
31	female	45,8	nd	17	nd	nd	nd	nd	nd	nd	nd	nd	nd	17	nd	nd	nd	nd	nd	nd	nd	nd	nd	nd	nd	nd	nd	131
32	female	46,4	nd	8	nd	nd	nd	nd	nd	nd	nd	nd	nd	8	nd	nd	nd	nd	nd	nd	nd	nd	nd	nd	nd	nd	nd	68
33	female	47,1	nd	7	nd	nd	nd	nd	nd	nd	nd	nd	nd	7	nd	nd	nd	nd	nd	nd	nd	nd	nd	nd	nd	nd	nd	55
34	female	61,1	nd	7	nd	nd	nd	nd	nd	nd	nd	nd	nd	7	nd	nd	nd	nd	nd	nd	nd	nd	nd	nd	nd	nd	nd	58
35	female	70,6	nd	6	nd	nd	nd	nd	nd	nd	nd	nd	nd	6	nd	nd	nd	nd	nd	nd	nd	nd	nd	nd	nd	nd	nd	50
36	male	3,3	nd	nd	nd	nd	nd	nd	nd	nd	nd	nd	nd	nd	nd	nd	nd	nd	nd	nd	nd	nd	nd	nd	nd	nd	nd	254
37	male	3,7	nd	nd	nd	nd	nd	nd	nd	nd	nd	nd	nd	nd	nd	nd	nd	nd	nd	nd	nd	nd	nd	nd	nd	nd	nd	84
38	male	4,1	nd	nd	nd	nd	nd	nd	nd	nd	nd	nd	nd	nd	nd	nd	nd	nd	nd	nd	nd	nd	nd	nd	nd	nd	nd	102
39	male	4,5	nd	nd	nd	nd	nd	nd	nd	nd	nd	nd	nd	nd	nd	nd	nd	nd	nd	nd	nd	nd	nd	nd	nd	nd	nd	109
40	male	6,1	nd	nd	nd	nd	nd	nd	nd	nd	nd	nd	nd	nd	nd	nd	nd	nd	nd	nd	nd	nd	nd	nd	nd	nd	nd	53
41	male	6,3	nd	nd	nd	nd	nd	nd	nd	nd	nd	nd	nd	nd	nd	nd	nd	nd	nd	nd	nd	nd	nd	nd	nd	nd	nd	52
42	male	8,8	nd	6	nd	nd	nd	nd	nd	nd	nd	nd	nd	7	nd	nd	nd	nd	nd	nd	nd	nd	nd	nd	nd	nd	nd	46
43	male	11,8	nd	6	nd	nd	nd	nd	nd	nd	nd	nd	nd	nd	nd	nd	nd	nd	nd	nd	nd	nd	nd	nd	nd	nd	nd	47
44	male	13,1	nd	5	nd	nd	nd	nd	nd	nd	nd	nd	nd	nd	nd	nd	nd	nd	nd	nd	nd	nd	nd	nd	nd	nd	nd	39
45	male	15,3	nd	4	nd	nd	nd	nd	nd	nd	nd	nd	nd	4	nd	nd	nd	nd	nd	nd	nd	nd	nd	nd	nd	nd	nd	28
46	male	16,2	nd	nd	nd	nd	nd	nd	nd	nd	nd	nd	nd	5	nd	nd	nd	nd	nd	nd	nd	nd	nd	nd	nd	nd	nd	35
47	male	19,5	nd	5	nd	nd	nd	nd	nd	nd	nd	nd	nd	nd	nd	nd	nd	nd	nd	nd	nd	nd	nd	nd	nd	nd	nd	46
48	male	27,0	nd	nd	nd	nd	nd	nd	nd	nd	nd	nd	nd	nd	nd	nd	nd	nd	nd	nd	nd	nd	nd	nd	nd	nd	nd	67
49	male	27,8	nd	8	nd	nd	nd	nd	nd	nd	nd	nd	nd	nd	nd	nd	nd	nd	nd	nd	nd	nd	nd	nd	nd	nd	nd	66
50	male	29,0	nd	nd	nd	nd	nd	nd	nd	nd	nd	nd	nd	nd	nd	nd	nd	nd	nd	nd	nd	nd	nd	nd	nd	nd	nd	44
51	male	32,5	nd	nd	nd	nd	nd	nd	nd	nd	nd	nd	nd	6	nd	nd	nd	nd	nd	nd	nd	nd	nd	nd	nd	nd	nd	86
52	male	33,2	nd	nd	nd	nd	nd	nd	nd	nd	nd	nd	nd	nd	nd	nd	nd	nd	nd	nd	nd	nd	nd	nd	nd	nd	nd	95
53	male	33,7	nd	nd	nd	nd	nd	nd	nd	nd	nd	nd	nd	6	nd	nd	nd	nd	nd	nd	nd	nd	nd	nd	nd	nd	nd	49
54	male	34,5	nd	nd	nd	nd	nd	nd	nd	nd	nd	nd	nd	nd	nd	nd	nd	nd	nd	nd	nd	nd	nd	nd	nd	nd	nd	35
55	male	36,8	nd	nd	nd	nd	nd	nd	nd	nd	nd	nd	nd	6	nd	nd	nd	nd	nd	nd	nd	nd	nd	nd	nd	nd	nd	60
56	male	44,3	nd	nd	nd	nd	nd	nd	nd	nd	nd	nd	nd	nd	nd	nd	nd	nd	nd	nd	nd	nd	nd	nd	nd	nd	nd	155
57	male	50,7	nd	6	nd	nd	nd	nd	nd	nd	nd	nd	nd	6	nd	nd	nd	nd	nd	nd	nd	nd	nd	nd	nd	nd	nd	52
58	male	54,4	nd	nd	nd	nd	nd	nd	nd	nd	nd	nd	nd	5	nd	nd	nd	nd	nd	nd	nd	nd	nd	nd	nd	nd	nd	79
59	male	60,7	nd	6	nd	nd	nd	nd	nd	nd	nd	nd	nd	nd	nd	nd	nd	nd	nd	nd	nd	nd	nd	nd	nd	nd	nd	49
**95th percentile**			**nd**	**37**	**nd**	**nd**	**nd**	**nd**	**nd**	**nd**	**nd**	**nd**	**nd**	**37**	**nd**	**nd**	**nd**	**nd**	**nd**	**nd**	**nd**	**nd**	**nd**	**nd**	**nd**	**nd**	**nd**	**293**

nd: not detected

Table S-11A.

A			Age range		Lyso-Sulf		GalSph		Lyso-GM2		Lyso-GM1		Lyso-SM	
			years		pmol/mmol creat.		pmol/mmol creat.		pmol/mmol creat.		pmol/mmol creat.		pmol/mmol creat.	
**SPHINGOLIPIDOSES (n=119)**		**n**	**Median**	**Range [min-max]**	**Median**	**Range [min-max]**	**Median**	**Range [min-max]**	**Median**	**Range [min-max]**	**Median**	**Range [min-max]**	**Median**	**Range [min-max]**
**Fabry disease**	**Untreated female (UFF)**	**33**	34.4	[8.4-62.4]	nd	n/a	nd	n/a	nd	n/a	nd	n/a	51	[22-756]
	Classical mutations	27	36.1	[8.4-62.4]	nd	n/a	nd	n/a	nd	n/a	nd	n/a	48	[22-306]
	Late-onset mutations	6	31.7	[11.1-52.1]	nd	n/a	nd	n/a	nd	n/a	nd	n/a	65	[51-756]
	**Treated female (TFF)**	**24**	57.2	[38.5-71.4]	nd	n/a	nd	n/a	nd	n/a	nd	n/a	75	[17-304]
	Classical mutations	22	56.0	[38.5-71.4]	nd	n/a	nd	n/a	nd	n/a	nd	n/a	71	[17-304]
	Late-onset mutations	2	62.6	[62.0-63.2]	nd	n/a	nd	n/a	nd	n/a	nd	n/a	92	[85-99]
	**Untreated male (UFM)**	**19**	28.1	[8.6-66.9]	nd	n/a	nd	n/a	nd	n/a	nd	n/a	92	[37-258]
	Classical mutations	11	23.9	[8.6-48.8]	nd	n/a	nd	n/a	nd	n/a	nd	n/a	102	[37-162]
	Late-onset mutations	8	37.1	[13.9-66.9]	nd	n/a	nd	n/a	nd	n/a	nd	n/a	76	[40-258]
	**Treated male (TFM)**	**22**	44.8	[25.1-66.4]	nd	n/a	nd	n/a	nd	n/a	nd	n/a	70	[26-211]
	Classical mutations	20	44.8	[25.1-60.1]	nd	n/a	nd	n/a	nd	n/a	nd	n/a	76	[34-211]
	Late-onset mutations	2	53.9	[41.3-66.4]	nd	n/a	nd	n/a	nd	n/a	nd	n/a	34	[26-42]
**Gaucher Disease**	**Untreated**	**9**	50.3	[42.8-70.7]	nd	n/a	nd	n/a	nd	n/a	nd	n/a	52	[13-203]
	**Treated**	**5**	16.0	[5.9-17.4]	nd	n/a	nd	n/a	nd	n/a	nd	n/a	36	[22-169]
**MLD**	**Treated**	**4**	n/a	n/a	nd	[nd-875]	nd	n/a	nd	n/a	nd	n/a	671	[255-869]
**GM1**	**Treated**	**2**	2.6	[1,8-3,5]	nd	n/a	nd	n/a	nd	n/a	nd	n/a	141	[53-228]
**NPC**	**Treated**	**1**	4.1	n/a	nd	n/a	nd	n/a	nd	n/a	nd	n/a	63	n/a
**PATHOLOGICAL CONTROLS (n=21)**		**n**	**Median**	**Range [min-max]**	**Median**	**Range [min-max]**	**Median**	**Range [min-max]**	**Median**	**Range [min-max]**	**Median**	**Range [min-max]**	**Median**	**Range [min-max]**
**Pompe**	**Treated**	**2**	6.2	[5.5-6.8]	nd	n/a	nd	n/a	nd	n/a	nd	n/a	68	[46-91]
**MPS**	**Type I (Hurler syndrome)**	**4**	9.3	[7.0-16.0]	nd	n/a	nd	n/a	nd	n/a	nd	n/a	83	[26-722]
	**Type II (Hunter syndrome)**	**5**	6.0	[5.0-14.9]	nd	n/a	nd	n/a	nd	n/a	nd	n/a	48	[37-65]
	**Type IIIB (Sanfilippo syndrome)**	**1**	1.8	n/a	nd	n/a	nd	n/a	nd	n/a	nd	n/a	155	n/a
	**Type IVA (Morquio syndrome)**	**6**	13.6	[3.1-36.3]	nd	n/a	nd	n/a	nd	n/a	nd	n/a	82	[28-860]
	**Type VI**	**2**	23.1	[4.0-42.2]	nd	n/a	nd	n/a	nd	n/a	nd	n/a	63	[31-96]
	**Type VII**	**1**	40.5	n/a	nd	n/a	nd	n/a	nd	n/a	nd	n/a	50	n/a
**HEALTHY CONTROLS (n=60)**		**n**	**Median**	**Range [min-max]**	**Median**	**Range [min-max]**	**Median**	**Range [min-max]**	**Median**	**Range [min-max]**	**Median**	**Range [min-max]**	**Median**	**Range [min-max]**
**Controls**	**All**	**59**	27.2	[2.6-70.6]	nd	n/a	nd	n/a	nd	n/a	nd	n/a	67	[nd-492]
	Male	24	23.3	[3.3-60.7]	nd	n/a	nd	n/a	nd	n/a	nd	n/a	46	[28-58]
	Female	35	27.3	[2.6-70.6]	nd	n/a	nd	n/a	nd	n/a	nd	n/a	105	[nd-492]
**NORMAL REFERENCE VALUE (95th percentile)**			**n/a**		**nd**		**nd**		**nd**		**nd**		**293**	

Table S-11B.

B			GluSph		GluSph (-28]		GluSph (-26]		GluSph (-12]		GluSph(+2]		GluSph (+14]		GluSph (+16]		GluSph (+30]		GluSph (+32]	
			pmol/mmol creat.		pmol/mmol creat.		pmol/mmol creat.		pmol/mmol creat.		pmol/mmol creat.		pmol/mmol creat.		pmol/mmol creat.		pmol/mmol creat.		pmol/mmol creat.	
**SPHINGOLIPIDOSES (n=119)**		**n**	**Median**	**Range [min-max]**	**Median**	**Range [min-max]**	**Median**	**Range [min-max]**	**Median**	**Range [min-max]**	**Median**	**Range [min-max]**	**Median**	**Range [min-max]**	**Median**	**Range [min-max]**	**Median**	**Range [min-max]**	**Median**	**Range [min-max]**
**Fabry disease**	**Untreated female (UFF)**	**33**	12	[nd-57]	nd	n/a	nd	n/a	nd	n/a	nd	n/a	nd	n/a	nd	n/a	nd	n/a	nd	[nd-831]
	Classical mutations	27	12	[nd-57]	nd	n/a	nd	n/a	nd	n/a	nd	n/a	nd	n/a	nd	n/a	nd	n/a	nd	[nd-831]
	Late-onset mutations	6	13	[nd-32]	nd	n/a	nd	n/a	nd	n/a	nd	n/a	nd	n/a	nd	n/a	nd	n/a	nd	n/a
	**Treated female (TFF)**	**24**	13	[nd-81]	nd	[nd-11]	nd	n/a	nd	n/a	nd	n/a	nd	n/a	nd	n/a	nd	n/a	nd	n/a
	Classical mutations	22	13	[nd-81]	nd	[nd-11]	nd	n/a	nd	n/a	nd	n/a	nd	n/a	nd	n/a	nd	n/a	nd	n/a
	Late-onset mutations	2	13	[nd-26]	nd	n/a	nd	n/a	nd	n/a	nd	n/a	nd	n/a	nd	n/a	nd	n/a	nd	n/a
	**Untreated male (UFM)**	**19**	nd	[nd-22]	nd	n/a	nd	n/a	nd	n/a	nd	n/a	nd	[nd-22]	nd	[nd-63]	nd	[nd-40]	nd	n/a
	Classical mutations	11	nd	[nd-22]	nd	n/a	nd	n/a	nd	n/a	nd	n/a	nd	[nd-22]	nd	[nd-63]	nd	[nd-40]	nd	n/a
	Late-onset mutations	8	nd	n/a	nd	n/a	nd	n/a	nd	n/a	nd	n/a	nd	n/a	nd	n/a	nd	n/a	nd	n/a
	**Treated male (TFM)**	**22**	9	[nd-26]	nd	n/a	nd	n/a	nd	n/a	nd	n/a	nd	[nd-30]	nd	[nd-36]	nd	[nd-18]	nd	n/a
	Classical mutations	20	10	[nd-26]	nd	n/a	nd	n/a	nd	n/a	nd	n/a	nd	[nd-30]	nd	[nd-36]	nd	[nd-18]	nd	n/a
	Late-onset mutations	2	7	[5-9]	nd	n/a	nd	n/a	nd	n/a	nd	n/a	nd	n/a	nd	n/a	nd	n/a	nd	n/a
**Gaucher Disease**	**Untreated**	**9**	50	[30-1071]	61	[nd-584]	2690	[nd-10081]	1060	[nd-6407]	993	[nd-6644]	697	[93-17208]	1463	[nd-13062]	1683	[78-5618]	197	[nd-6654]
	**Treated**	**5**	38	[9-66]	25	[nd-84]	472	[19-1250]	354	[nd-543]	362	[17-525]	281	[18-1454]	426	[33-1035]	575	[17-647]	215	[nd-575]
**MLD**	**Treated**	**4**	94	[nd-267]	nd	n/a	nd	n/a	nd	n/a	nd	n/a	nd	n/a	nd	n/a	nd	n/a	nd	n/a
**GM1**	**Treated**	**2**	nd	n/a	nd	n/a	25	[nd-49]	nd	n/a	nd	n/a	nd	n/a	nd	n/a	nd	n/a	nd	n/a
**NPC**	**Treated**	**1**	16	n/a	nd	n/a	34	n/a	nd	n/a	24	n/a	nd	n/a	nd	n/a	62	n/a	nd	n/a
**PATHOLOGICAL CONTROLS (n=21)**		**n**	**Median**	**Range [min-max]**	**Median**	**Range [min-max]**	**Median**	**Range [min-max]**	**Median**	**Range [min-max]**	**Median**	**Range [min-max]**	**Median**	**Range [min-max]**	**Median**	**Range [min-max]**	**Median**	**Range [min-max]**	**Median**	**Range [min-max]**
**Pompe**	**Treated**	**2**	20	[13-27]	nd	n/a	41	[19-62]	nd	n/a	nd	n/a	nd	n/a	nd	n/a	67	[39-95]	nd	n/a
**MPS**	**Type I (Hurler syndrome)**	**4**	nd	[nd-127]	nd	n/a	nd	n/a	nd	n/a	nd	n/a	nd	n/a	nd	n/a	nd	n/a	nd	n/a
	**Type II (Hunter syndrome)**	**5**	nd	n/a	nd	n/a	nd	n/a	nd	n/a	nd	n/a	nd	n/a	nd	n/a	nd	[nd-30]	nd	n/a
	**Type IIIB (Sanfilippo syndrome)**	**1**	nd	n/a	nd	n/a	nd	n/a	nd	n/a	nd	n/a	nd	n/a	nd	n/a	nd	n/a	nd	n/a
	**Type IVA (Morquio syndrome)**	**6**	nd	n/a	nd	n/a	nd	n/a	nd	n/a	nd	n/a	nd	n/a	nd	n/a	nd	n/a	nd	n/a
	**Type VI**	**2**	nd	n/a	nd	n/a	nd	n/a	nd	n/a	nd	n/a	nd	n/a	nd	n/a	nd	n/a	nd	n/a
	**Type VII**	**1**	nd	n/a	nd	n/a	nd	n/a	nd	n/a	nd	n/a	62	n/a	61	n/a	46	n/a	nd	n/a
**HEALTHY CONTROLS (n=60)**		**n**	**Median**	**Range [min-max]**	**Median**	**Range [min-max]**	**Median**	**Range [min-max]**	**Median**	**Range [min-max]**	**Median**	**Range [min-max]**	**Median**	**Range [min-max]**	**Median**	**Range [min-max]**	**Median**	**Range [min-max]**	**Median**	**Range [min-max]**
**Controls**	**All**	**59**	6	[nd-53]	nd	n/a	nd	n/a	nd	n/a	nd	n/a	nd	n/a	nd	n/a	nd	n/a	nd	n/a
	Male	24	nd	[nd-8]	nd	n/a	nd	n/a	nd	n/a	nd	n/a	nd	n/a	nd	n/a	nd	n/a	nd	n/a
	Female	35	8	[nd-53]	nd	n/a	nd	n/a	nd	n/a	nd	n/a	nd	n/a	nd	n/a	nd	n/a	nd	n/a
**NORMAL REFERENCE VALUE (95th percentile)**			**37**		**nd**		**nd**		**nd**		**nd**		**nd**		**nd**		**nd**		**nd**	

Table S-11C.

C			Lyso-Gb_3		Lyso-Gb_3 (-28)		Lyso-Gb_3 (-12)		Lyso-Gb_3 (-2)		Lyso-Gb_3 (+14)		Lyso-Gb_3 (+16)		Lyso-Gb_3 (+34)		Lyso-Gb_3 (+50)	
			pmol/mmol creat.		pmol/mmol creat.		pmol/mmol creat.		pmol/mmol creat.		pmol/mmol creat.		pmol/mmol creat.		pmol/mmol creat.		pmol/mmol creat.	
**SPHINGOLIPIDOSES (n=119)**		**n**	**Median**	**Range [min-max]**	**Median**	**Range [min-max]**	**Median**	**Range [min-max]**	**Median**	**Range [min-max]**	**Median**	**Range [min-max]**	**Median**	**Range [min-max]**	**Median**	**Range [min-max]**	**Median**	**Range [min-max]**
**Fabry disease**	**Untreated female (UFF)**	**33**	19	[nd-114]	nd	[nd-88]	nd	[nd-101]	nd	n/a	nd	[nd-51]	38	[nd-288]	37	[nd-382]	nd	[nd-164]
	Classical mutations	27	21	[nd-114]	nd	n/a	nd	[nd-101]	nd	n/a	nd	[nd-51]	5nd	[nd-288]	42	[nd-382]	22	[nd-164]
	Late-onset mutations	6	nd	n/a	nd	[nd-88]	nd	n/a	nd	n/a	nd	n/a	nd	n/a	nd	n/a	nd	n/a
	**Treated female (TFF)**	**24**	5	[nd-63]	nd	n/a	nd	[nd-163]	nd	n/a	nd	[nd-88]	64	[nd-331]	78	[nd-156]	37	[nd-145]
	Classical mutations	22	15	[nd-63]	nd	n/a	nd	[nd-163]	nd	n/a	nd	[nd-88]	64	[nd-331]	85	[nd-156]	44	[nd-145]
	Late-onset mutations	2	nd	n/a	nd	n/a	nd	n/a	nd	n/a	nd	n/a	39	[nd-78]	nd	n/a	nd	n/a
	**Untreated male (UFM)**	**19**	24	[nd-491]	nd	[nd-286]	103	[nd-2212]	nd	[nd-497]	21	[nd-1089]	120	[nd-5048]	157	[nd-1906]	182	[nd-3853]
	Classical mutations	11	54	[nd-491]	23	[nd-286]	390	[nd-2212]	nd	[nd-497]	210	[nd-1089]	1039	[nd-5048]	1033	[120-1906]	2251	[177-3853]
	Late-onset mutations	8	nd	[nd-27]	nd	n/a	nd	[nd-103]	nd	n/a	nd	[nd-21]	54	[nd-120]	18	[nd-57]	nd	[nd-48]
	**Treated male (TFM)**	**22**	25	[nd-233]	nd	[nd-100]	102	[nd-554]	nd	[nd-142]	18	[nd-479]	213	[26-2048]	154	[nd-795]	139	[nd-939]
	Classical mutations	20	30	[nd-233]	nd	[nd-100]	122	[nd-554]	nd	[nd-142]	25	[nd-479]	220	[76-2048]	169	[nd-795]	141	[nd-939]
	Late-onset mutations	2	6	[nd-12]	nd	n/a	nd	n/a	nd	n/a	nd	n/a	31	[26-35]	22	[15-29]	nd	n/a
**Gaucher Disease**	**Untreated**	**9**	nd	n/a	nd	n/a	nd	n/a	nd	n/a	nd	n/a	nd	[nd-220]	nd	n/a	nd	n/a
	**Treated**	**5**	nd	n/a	nd	n/a	nd	n/a	nd	n/a	nd	n/a	nd	[nd-32]	nd	n/a	nd	n/a
**MLD**	**Treated**	**4**	nd	n/a	nd	n/a	nd	n/a	nd	n/a	nd	n/a	nd	n/a	nd	n/a	nd	n/a
**GM1**	**Treated**	**2**	nd	n/a	nd	n/a	nd	n/a	nd	n/a	nd	n/a	nd	n/a	nd	n/a	nd	n/a
**NPC**	**Treated**	**1**	nd	n/a	nd	n/a	nd	n/a	nd	n/a	nd	n/a	nd	n/a	nd	n/a	nd	n/a
**PATHOLOGICAL CONTROLS (n=21)**		**n**	**Median**	**Range [min-max]**	**Median**	**Range [min-max]**	**Median**	**Range [min-max]**	**Median**	**Range [min-max]**	**Median**	**Range [min-max]**	**Median**	**Range [min-max]**	**Median**	**Range [min-max]**	**Median**	**Range [min-max]**
**Pompe**	**Treated**	**2**	nd	n/a	nd	n/a	nd	n/a	nd	n/a	nd	n/a	nd	n/a	nd	n/a	nd	n/a
**MPS**	**Type I (Hurler syndrome)**	**4**	nd	n/a	nd	n/a	nd	n/a	nd	n/a	nd	n/a	nd	n/a	nd	n/a	nd	n/a
	**Type II (Hunter syndrome)**	**5**	nd	n/a	nd	n/a	nd	[nd-118]	nd	n/a	nd	n/a	nd	[nd-112]	nd	[nd-32]	nd	n/a
	**Type IIIB (Sanfilippo syndrome)**	**1**	nd	n/a	nd	n/a	nd	n/a	nd	n/a	nd	n/a	nd	n/a	nd	n/a	nd	n/a
	**Type IVA (Morquio syndrome)**	**6**	nd	n/a	nd	n/a	nd	n/a	nd	n/a	nd	n/a	nd	n/a	nd	n/a	nd	n/a
	**Type VI**	**2**	nd	n/a	nd	n/a	nd	n/a	nd	n/a	nd	n/a	nd	n/a	nd	n/a	nd	n/a
	**Type VII**	**1**	nd	n/a	nd	n/a	nd	n/a	nd	n/a	nd	n/a	51	n/a	nd	n/a	nd	n/a
**HEALTHY CONTROLS (n=60)**		**n**	**Median**	**Range [min-max]**	**Median**	**Range [min-max]**	**Median**	**Range [min-max]**	**Median**	**Range [min-max]**	**Median**	**Range [min-max]**	**Median**	**Range [min-max]**	**Median**	**Range [min-max]**	**Median**	**Range [min-max]**
**Controls**	**All**	**59**	nd	[nd-25]	nd	n/a	nd	n/a	nd	n/a	nd	n/a	nd	n/a	nd	n/a	nd	n/a
	Male	24	nd	n/a	nd	n/a	nd	n/a	nd	n/a	nd	n/a	nd	n/a	nd	n/a	nd	n/a
	Female	35	nd	[nd-25]	nd	n/a	nd	n/a	nd	n/a	nd	n/a	nd	n/a	nd	n/a	nd	n/a
**NORMAL REFERENCE VALUE (95th percentile)**			**nd**		**nd**		**nd**		**nd**		**nd**		**nd**		**nd**		**nd**	

Table S-11D.

D			Regouped GluSph related analogs		GluSph and its related analogs		Regouped Lyso-Gb_3 related analogs		Lyso-Gb_3 and its related analogs	
			pmol/mmol creat.		pmol/mmol creat.		pmol/mmol creat.		pmol/mmol creat.	
**SPHINGOLIPIDOSES (n=119)**		**n**	**Median**	**Range [min-max]**	**Median**	**Range [min-max]**	**Median**	**Range [min-max]**	**Median**	**Range [min-max]**
**Fabry disease**	**Untreated female (UFF)**	**33**	nd	[nd-831]	13	[nd-831]	90	[nd-982]	111	[nd-1068]
	Classical mutations	27	nd	[nd-831]	13	[nd-831]	125	[nd-982]	154	[nd-1068]
	Late-onset mutations	6	nd	n/a	13	[nd-32]	nd	[nd-88]	nd	[nd-88]
	**Treated female (TFF)**	**24**	nd	[nd-11]	13	[nd-81]	200	[nd-845]	226	[nd-894]
	Classical mutations	22	nd	[nd-11]	13	[nd-81]	213	[nd-845]	242	[nd-894]
	Late-onset mutations	2	nd	n/a	13	[nd-26]	39	[nd-78]	39	[nd-78]
	**Untreated male (UFM)**	**19**	nd	[nd-125]	nd	[nd-125]	409	[nd-15382]	409	[nd-15382]
	Classical mutations	11	nd	[nd-125]	nd	[nd-125]	4440	[nd-14891]	4440	[387-15382]
	Late-onset mutations	8	nd	n/a	nd	n/a	90	[nd-306]	90	[nd-330]
	**Treated male (TFM)**	**22**	nd	[nd-41]	10	[nd-59]	589	[50-4118]	606	[55-4351]
	Classical mutations	20	nd	[nd-41]	2	[nd-59]	656	[177-4118]	684	[186-4351]
	Late-onset mutations	2	nd	n/a	6	[5-9]	53	[50-55]	59	[55-62]
**Gaucher Disease**	**Untreated**	**9**	9549	[171-66258]	9690	[238-67330]	nd	[nd-220]	nd	[nd-220]
	**Treated**	**5**	3340	[105-5276]	3406	[114-5314]	nd	[nd-32]	nd	[nd-32]
**MLD**	**Treated**	**4**	nd	n/a	nd	[nd-167]	nd	n/a	nd	n/a
**GM1**	**Treated**	**2**	25	[0-25]	25	[0-25]	nd	n/a	nd	n/a
**NPC**	**Treated**	**1**	120	n/a	136	n/a	32	n/a	32	n/a
**PATHOLOGICAL CONTROLS (n=21)**		**n**	**Median**	**Range [min-max]**	**Median**	**Range [min-max]**	**Median**	**Range [min-max]**	**Median**	**Range [min-max]**
**Pompe**	**Treated**	**2**	108	[59-158]	128	[72-185]	nd	n/a	nd	n/a
**MPS**	**Type I (Hurler syndrome)**	**4**	nd	n/a	nd	[0-127]	nd	n/a	nd	n/a
	**Type II (Hunter syndrome)**	**5**	nd	[nd-30]	nd	[nd-30]	nd	[nd-229]	nd	[nd-229]
	**Type IIIB (Sanfilippo syndrome)**	**1**	nd	n/a	nd	n/a	nd	n/a	nd	n/a
	**Type IVA (Morquio syndrome)**	**6**	nd	n/a	nd	n/a	nd	n/a	nd	n/a
	**Type VI**	**2**	nd	n/a	nd	n/a	nd	n/a	nd	n/a
	**Type VII**	**1**	169	n/a	169	n/a	51	n/a	51	n/a
**HEALTHY CONTROLS (n=60)**		**n**	**Median**	**Range [min-max]**	**Median**	**Range [min-max]**	**Median**	**Range [min-max]**	**Median**	**Range [min-max]**
**Controls**	**All**	**59**	nd	n/a	6	[nd-53]	nd	n/a	nd	[nd-25]
	Male	24	nd	n/a	6	[nd-8]	nd	n/a	nd	n/a
	Female	35	nd	n/a	8	[nd-53]	nd	n/a	nd	[nd-25]
**NORMAL REFERENCE VALUE (95th percentile)**			**nd**		**37**		**nd**		**nd**	

Table S-12.

	GluSph	GluSph related analogs only	GluSph and its related analogs
**Kruskal-Wallis test**	**P-value**	**P-value**	**P-value**
5 groups comparison	****	****	****
**Dunn's multiple comparisons test**	**P-value**	**P-value**	**P-value**
CTRL vs. LSD	ns	ns	ns
CTRL vs. Sph	ns	ns	ns
CTRL vs. TG	*	****	***
CTRL vs. UG	****	****	****
LSD vs. Sph	**	ns	ns
LSD vs. TG	**	****	***
LSD vs. UG	****	****	****
Sph vs. TG	ns	****	*
Sph vs. UG	**	****	***
TG vs. UG	ns	ns	ns

P-value signifiance: ns: >0.05; *: <0.05; **: <0.01; ***: <0.001; ****: <0.0001

Table S-13A.

Classical mutations	Lyso-Gb_3	Lyso-Gb_3 related analogs only	Lyso-Gb_3 and its related analogs
**Kruskal-Wallis test**	**P-Value**	**P-Value**	**P-Value**
7 groups comparaisons	****	****	****
**Dunn's multiple comparisons test**	**P-Value**	**P-Value**	**P-Value**
n=59 - CTRL vs. n=21 - LSD	ns	ns	ns
n=59 - CTRL vs. n=20 - Sph	ns	ns	ns
n=59 - CTRL vs. n=22 - TFF	***	****	****
n=59 - CTRL vs. n=27 - UFF	****	****	****
n=59 - CTRL vs. n=20 - TFM	****	****	****
n=59 - CTRL vs. n=11 - UFM	****	****	****
n=21 - LSD vs. n=20 - Sph	ns	ns	ns
n=21 - LSD vs. n=22 - TFF	*	***	***
n=21 - LSD vs. n=27 - UFF	****	**	***
n=21 - LSD vs. n=20 - TFM	****	****	****
n=21 - LSD vs. n=11 - UFM	****	****	****
n=20 - Sph vs. n=22 - TFF	*	***	***
n=20 - Sph vs. n=27 - UFF	****	**	***
n=20 - Sph vs. n=20 - TFM	****	****	****
n=20 - Sph vs. n=11 - UFM	****	****	****
n=22 - TFF vs. n=27 - UFF	ns	ns	ns
n=22 - TFF vs. n=20 - TFM	ns	ns	ns
n=22 - TFF vs. n=11 - UFM	ns	ns	ns
n=27 - UFF vs. n=20 - TFM	ns	ns	ns
n=27 - UFF vs. n=11 - UFM	ns	*	ns
n=20 - TFM vs. n=11 - UFM	ns	ns	ns

P-value signifiance: ns: >0.05; *: <0.05; **: <0.01; ***: <0.001; ****: <0.0001

Table S-13B.

Later-Onset mutations	Lyso-Gb_3	Lyso-Gb_3 related analogs only	Lyso-Gb_3 and its related analogs
**Kruskal-Wallis test**	**P-Value**	**P-Value**	**P-Value**
7 groups comparaisons	****	****	****
**Dunn's multiple comparisons test**	**P-Value**	**P-Value**	**P-Value**
n=59 - CTRL vs. n=21 - LSD	ns	ns	ns
n=59 - CTRL vs. n=20 - Sph	ns	ns	ns
n=59 - CTRL vs. n=2 - TFF	ns	ns	ns
n=59 - CTRL vs. n=6 - UFF	ns	ns	ns
n=59 - CTRL vs. n=2 - TFM	**	**	**
n=59 - CTRL vs. n=8 - UFM	*	****	****
n=21 - LSD vs. n=20 - Sph	ns	ns	ns
n=21 - LSD vs. n=2 - TFF	ns	ns	ns
n=21 - LSD vs. n=6 - UFF	ns	ns	ns
n=21 - LSD vs. n=2 - TFM	**	*	*
n=21 - LSD vs. n=8 - UFM	*	**	*
n=20 - Sph vs. n=2 - TFF	ns	ns	ns
n=20 - Sph vs. n=6 - UFF	ns	ns	ns
n=20 - Sph vs. n=2 - TFM	**	ns	ns
n=20 - Sph vs. n=8 - UFM	*	*	*
n=2 - TFF vs. n=6 - UFF	ns	ns	ns
n=2 - TFF vs. n=2 - TFM	ns	ns	ns
n=2 - TFF vs. n=8 - UFM	ns	ns	ns
n=6 - UFF vs. n=2 - TFM	*	ns	ns
n=6 - UFF vs. n=8 - UFM	ns	ns	ns
n=2 - TFM vs. n=8 - UFM	ns	ns	ns

P-value signifiance: ns: >0.05; *: <0.05; **: <0.01; ***: <0.001; ****: <0.0001

Table S-14.

A)

UFF					
**Lyso-Gb$_3$ only**					
Cutoff value (pmol/mmol creatinine)	Sensitivity%	95% CI	Specificity%	95% CI	Youden index
**> 5.36**	**63.6**	**46.6% to 77.8%**	**98.3**	**91.1% to 99.9%**	**0.619**
> 12.3	60.6	43.7% to 75.3%	98.3	91.1% to 99.9%	0.589
> 14.2	57.6	40.8% to 72.8%	98.3	91.1% to 99.9%	0.559
> 16.4	54.5	38.0% to 70.2%	98.3	91.1% to 99.9%	0.528
> 18.4	51.5	35.2% to 67.5%	98.3	91.1% to 99.9%	0.498
> 18.7	48.5	32.5% to 64.8%	98.3	91.1% to 99.9%	0.468
> 19.7	45.5	29.8% to 62.0%	98.3	91.1% to 99.9%	0.438
> 21.1	42.4	27.2% to 59.2%	98.3	91.1% to 99.9%	0.407
> 22.9	39.4	24.7% to 56.3%	98.3	91.1% to 99.9%	0.377
> 24.7	36.4	22.2% to 53.4%	98.3	91.1% to 99.9%	0.347
> 27.3	36.4	22.2% to 53.4%	100	94.0% to 100%	0.364
> 35.0	33.3	19.8% to 50.4%	100	94.0% to 100%	0.333
> 41.9	30.3	17.4% to 47.3%	100	94.0% to 100%	0.303
> 45.4	27.3	15.1% to 44.2%	100	94.0% to 100%	0.273
> 47.9	24.2	12.8% to 41.0%	100	94.0% to 100%	0.242
> 50.2	21.2	10.7% to 37.8%	100	94.0% to 100%	0.212
> 59.3	18.2	8.61% to 34.4%	100	94.0% to 100%	0.182
> 66.8	15.2	6.65% to 30.9%	100	94.0% to 100%	0.152
> 68.1	12.1	4.82% to 27.3%	100	94.0% to 100%	0.121
> 77.6	9.09	3.14% to 23.6%	100	94.0% to 100%	0.0909
> 87.0	6.06	1.08% to 19.6%	100	94.0% to 100%	0.0606
> 101	3.03	0.155% to 15.3%	100	94.0% to 100%	0.0303
**Lyso-Gb$_3$ related analogs only**					
Cutoff value (pmol/mmol creatinine)	Sensitivity%	95% CI	Specificity%	95% CI	Youden index
**> 13.6**	**72.7**	**55.8% to 84.9%**	**100**	**94.0% to 100%**	**0.727**
> 28.4	69.7	52.7% to 82.6%	100	94.0% to 100%	0.697
> 43.5	66.7	49.6% to 80.2%	100	94.0% to 100%	0.667
> 60.9	63.6	46.6% to 77.8%	100	94.0% to 100%	0.636
> 75.5	60.6	43.7% to 75.3%	100	94.0% to 100%	0.606
> 87.0	57.6	40.8% to 72.8%	100	94.0% to 100%	0.576
> 87.9	54.5	38.0% to 70.2%	100	94.0% to 100%	0.545
> 89.2	51.5	35.2% to 67.5%	100	94.0% to 100%	0.515
> 97.8	48.5	32.5% to 64.8%	100	94.0% to 100%	0.485
> 107	45.5	29.8% to 62.0%	100	94.0% to 100%	0.455
> 117	42.4	27.2% to 59.2%	100	94.0% to 100%	0.424
> 151	39.4	24.7% to 56.3%	100	94.0% to 100%	0.394
> 180	36.4	22.2% to 53.4%	100	94.0% to 100%	0.364
> 189	33.3	19.8% to 50.4%	100	94.0% to 100%	0.333
> 229	30.3	17.4% to 47.3%	100	94.0% to 100%	0.303
> 271	27.3	15.1% to 44.2%	100	94.0% to 100%	0.273
> 280	24.2	12.8% to 41.0%	100	94.0% to 100%	0.242
> 282	21.2	10.7% to 37.8%	100	94.0% to 100%	0.212
> 286	18.2	8.61% to 34.4%	100	94.0% to 100%	0.182
> 308	15.2	6.65% to 30.9%	100	94.0% to 100%	0.152
> 333	12.1	4.82% to 27.3%	100	94.0% to 100%	0.121
> 401	9.09	3.14% to 23.6%	100	94.0% to 100%	0.0909
> 479	6.06	1.08% to 19.6%	100	94.0% to 100%	0.0606
> 740	3.03	0.155% to 15.3%	100	94.0% to 100%	0.0303
**Lyso-Gb$_3$ and its related analogs**					
Cutoff value (pmol/mmol creatinine)	Sensitivity%	95% CI	Specificity%	95% CI	Youden index
> 12.6	75.8	59.0% to 87.2%	98.3	91.1% to 99.9%	0.741
**> 27.4**	**75.8**	**59.0% to 87.2%**	**100**	**94.0% to 100%**	**0.758**
> 35.7	72.7	55.8% to 84.9%	100	94.0% to 100%	0.727
> 49.6	69.7	52.7% to 82.6%	100	94.0% to 100%	0.697
> 63.3	66.7	49.6% to 80.2%	100	94.0% to 100%	0.667
> 78.6	63.6	46.6% to 77.8%	100	94.0% to 100%	0.636
> 97.0	60.6	43.7% to 75.3%	100	94.0% to 100%	0.606
> 107	57.6	40.8% to 72.8%	100	94.0% to 100%	0.576
> 108	54.5	38.0% to 70.2%	100	94.0% to 100%	0.545
> 109	51.5	35.2% to 67.5%	100	94.0% to 100%	0.515
> 115	48.5	32.5% to 64.8%	100	94.0% to 100%	0.485
> 132	45.5	29.8% to 62.0%	100	94.0% to 100%	0.455
> 150	42.4	27.2% to 59.2%	100	94.0% to 100%	0.424
> 172	39.4	24.7% to 56.3%	100	94.0% to 100%	0.394
> 211	36.4	22.2% to 53.4%	100	94.0% to 100%	0.364
> 258	33.3	19.8% to 50.4%	100	94.0% to 100%	0.333
> 291	30.3	17.4% to 47.3%	100	94.0% to 100%	0.303
> 303	27.3	15.1% to 44.2%	100	94.0% to 100%	0.273
> 319	24.2	12.8% to 41.0%	100	94.0% to 100%	0.242
> 333	21.2	10.7% to 37.8%	100	94.0% to 100%	0.212
> 347	18.2	8.61% to 34.4%	100	94.0% to 100%	0.182
> 376	15.2	6.65% to 30.9%	100	94.0% to 100%	0.152
> 394	12.1	4.82% to 27.3%	100	94.0% to 100%	0.121
> 429	9.09	3.14% to 23.6%	100	94.0% to 100%	0.0909
> 505	6.06	1.08% to 19.6%	100	94.0% to 100%	0.0606
> 809	3.03	0.155% to 15.3%	100	94.0% to 100%	0.0303

B)

UFM					
**Lyso-Gb3 only**					
Cutoff value (pmol/mmol creatinine)	Sensitivity%	95% CI	Specificity%	95% CI	Youden index
**> 12.3**	**52.6**	**31.7% to 72.7%**	**98.3**	**91.1% to 99.9%**	**0.509**
> 24.8	47.4	27.3% to 68.3%	98.3	91.1% to 99.9%	0.457
> 26.0	47.4	27.3% to 68.3%	100	94.0% to 100%	0.474
> 35.7	42.1	23.1% to 63.7%	100	94.0% to 100%	0.421
> 45.1	36.8	19.1% to 59.0%	100	94.0% to 100%	0.368
> 49.6	31.6	15.4% to 54.0%	100	94.0% to 100%	0.316
> 54.6	26.3	11.8% to 48.8%	100	94.0% to 100%	0.263
> 78.2	21.1	8.51% to 43.3%	100	94.0% to 100%	0.211
> 104	15.8	5.52% to 37.6%	100	94.0% to 100%	0.158
> 236	10.5	1.87% to 31.4%	100	94.0% to 100%	0.105
> 427	5.26	0.270% to 24.6%	100	94.0% to 100%	0.0526
**Lyso-Gb3 related analogs only**					
Cutoff value (pmol/mmol creatinine)	Sensitivity%	95% CI	Specificity%	95% CI	Youden index
**> 40.6**	**84.2**	**62.4% to 94.5%**	**100**	**94.0% to 100%**	**0.842**
> 89.9	78.9	56.7% to 91.5%	100	94.0% to 100%	0.789
> 122	73.7	51.2% to 88.2%	100	94.0% to 100%	0.737
> 158	68.4	46.0% to 84.6%	100	94.0% to 100%	0.684
> 238	63.2	41.0% to 80.9%	100	94.0% to 100%	0.632
> 346	57.9	36.3% to 76.9%	100	94.0% to 100%	0.579
> 398	52.6	31.7% to 72.7%	100	94.0% to 100%	0.526
> 911	47.4	27.3% to 68.3%	100	94.0% to 100%	0.474
> 1574	42.1	23.1% to 63.7%	100	94.0% to 100%	0.421
> 2986	36.8	19.1% to 59.0%	100	94.0% to 100%	0.368
> 4339	31.6	15.4% to 54.0%	100	94.0% to 100%	0.316
> 5352	26.3	11.8% to 48.8%	100	94.0% to 100%	0.263
> 6714	21.1	8.51% to 43.3%	100	94.0% to 100%	0.211
> 8247	15.8	5.52% to 37.6%	100	94.0% to 100%	0.158
> 10039	10.5	1.87% to 31.4%	100	94.0% to 100%	0.105
> 12820	5.26	0.270% to 24.6%	100	94.0% to 100%	0.0526
**Lyso-Gb3 and his related analogs**					
Cutoff value (pmol/mmol creatinine)	Sensitivity%	95% CI	Specificity%	95% CI	Youden index
**> 12.6**	**84.2**	**62.4% to 94.5%**	**98.3**	**91.1% to 99.9%**	**0.825**
> 61.8	84.2	62.4% to 94.5%	100	94.0% to 100%	0.842
> 103	78.9	56.7% to 91.5%	100	94.0% to 100%	0.789
> 127	73.7	51.2% to 88.2%	100	94.0% to 100%	0.737
> 158	68.4	46.0% to 84.6%	100	94.0% to 100%	0.684
> 251	63.2	41.0% to 80.9%	100	94.0% to 100%	0.632
> 358	57.9	36.3% to 76.9%	100	94.0% to 100%	0.579
> 398	52.6	31.7% to 72.7%	100	94.0% to 100%	0.526
> 934	47.4	27.3% to 68.3%	100	94.0% to 100%	0.474
> 1624	42.1	23.1% to 63.7%	100	94.0% to 100%	0.421
> 3014	36.8	19.1% to 59.0%	100	94.0% to 100%	0.368
> 4521	31.6	15.4% to 54.0%	100	94.0% to 100%	0.316
> 5556	26.3	11.8% to 48.8%	100	94.0% to 100%	0.263
> 6791	21.1	8.51% to 43.3%	100	94.0% to 100%	0.211
> 8327	15.8	5.52% to 37.6%	100	94.0% to 100%	0.158
> 10116	10.5	1.87% to 31.4%	100	94.0% to 100%	0.105
> 13116	5.26	0.270% to 24.6%	100	94.0% to 100%	0.0526

C)

UG					
**GluSph only**					
Cutoff value (pmol/mmol creatinine)	Sensitivity%	95% CI	Specificity%	95% CI	Youden index
> 7.38	100	67.6% to 100%	64.4	51.7% to 75.4%	0.644
> 7.48	100	67.6% to 100%	66.1	53.4% to 76.9%	0.661
> 7.75	100	67.6% to 100%	67.8	55.1% to 78.3%	0.678
> 8.18	100	67.6% to 100%	69.5	56.9% to 79.7%	0.695
> 8.41	100	67.6% to 100%	71.2	58.6% to 81.2%	0.712
> 8.49	100	67.6% to 100%	72.9	60.4% to 82.6%	0.729
> 9.26	100	67.6% to 100%	74.6	62.2% to 83.9%	0.746
> 10.1	100	67.6% to 100%	76.3	64.0% to 85.3%	0.763
> 10.3	100	67.6% to 100%	78	65.9% to 86.6%	0.78
> 11.0	100	67.6% to 100%	79.7	67.7% to 88.0%	0.797
> 11.6	100	67.6% to 100%	81.4	69.6% to 89.3%	0.814
> 12.0	100	67.6% to 100%	83.1	71.5% to 90.5%	0.831
> 12.5	100	67.6% to 100%	84.7	73.5% to 91.8%	0.847
> 13.2	100	67.6% to 100%	86.4	75.5% to 93.0%	0.864
> 15.1	100	67.6% to 100%	88.1	77.5% to 94.1%	0.881
> 20.4	100	67.6% to 100%	89.8	79.5% to 95.3%	0.898
> 26.2	100	67.6% to 100%	91.5	81.6% to 96.3%	0.915
**> 29.2**	**100**	**67.6% to 100%**	**93.2**	**83.8% to 97.3%**	**0.932**
> 30.5	87.5	52.9% to 99.4%	93.2	83.8% to 97.3%	0.807
> 31.5	75	40.9% to 95.6%	93.2	83.8% to 97.3%	0.682
> 33.0	62.5	30.6% to 86.3%	93.2	83.8% to 97.3%	0.557
> 35.6	50	21.5% to 78.5%	93.2	83.8% to 97.3%	0.432
> 37.3	50	21.5% to 78.5%	94.9	86.1% to 98.6%	0.449
> 42.1	50	21.5% to 78.5%	96.6	88.5% to 99.4%	0.466
> 49.9	50	21.5% to 78.5%	98.3	91.0% to 99.9%	0.483
> 60.0	50	21.5% to 78.5%	100	93.9% to 100%	0.500
> 159	37.5	13.7% to 69.4%	100	93.9% to 100%	0.375
> 253	25	4.44% to 59.1%	100	93.9% to 100%	0.250
> 663	12.5	0.641% to 47.1%	100	93.9% to 100%	0.125
**GluSph related analogs only**					
Cutoff value (pmol/mmol creatinine)	Sensitivity%	95% CI	Specificity%	95% CI	Youden index
**> 85.5**	**100**	**67.6% to 100%**	**100**	**93.9% to 100%**	**1,000**
> 1841	87.5	52.9% to 99.4%	100	93.9% to 100%	0.875
> 4629	75	40.9% to 95.6%	100	93.9% to 100%	0.75
> 7162	62.5	30.6% to 86.3%	100	93.9% to 100%	0.625
> 9549	50	21.5% to 78.5%	100	93.9% to 100%	0.5
> 11005	37.5	13.7% to 69.4%	100	93.9% to 100%	0.375
> 11950	25	4.44% to 59.1%	100	93.9% to 100%	0.25
> 39334	12.5	0.641% to 47.1%	100	93.9% to 100%	0.125
**GluSph and its related analogs**					
Cutoff value (pmol/mmol creatinine)	Sensitivity%	95% CI	Specificity%	95% CI	Youden index
> 1.80	100	67.6% to 100%	40.7	29.1% to 53.4%	0.407
> 3.65	100	67.6% to 100%	42.4	30.6% to 55.1%	0.424
> 4.22	100	67.6% to 100%	44.1	32.2% to 56.7%	0.441
> 4.94	100	67.6% to 100%	45.8	33.7% to 58.3%	0.458
> 5.30	100	67.6% to 100%	47.5	35.3% to 60.0%	0.475
> 5.48	100	67.6% to 100%	49.2	36.8% to 61.6%	0.492
> 5.57	100	67.6% to 100%	50.8	38.4% to 63.2%	0.508
> 5.71	100	67.6% to 100%	52.5	40.0% to 64.7%	0.525
> 5.89	100	67.6% to 100%	54.2	41.7% to 66.3%	0.542
> 6.07	100	67.6% to 100%	55.9	43.3% to 67.8%	0.559
> 6.33	100	67.6% to 100%	57.6	44.9% to 69.4%	0.576
> 6.69	100	67.6% to 100%	59.3	46.6% to 70.9%	0.593
> 7.03	100	67.6% to 100%	61	48.3% to 72.4%	0.610
> 7.24	100	67.6% to 100%	62.7	50.0% to 73.9%	0.627
> 7.38	100	67.6% to 100%	64.4	51.7% to 75.4%	0.644
> 7.48	100	67.6% to 100%	66.1	53.4% to 76.9%	0.661
> 7.75	100	67.6% to 100%	67.8	55.1% to 78.3%	0.678
> 8.18	100	67.6% to 100%	69.5	56.9% to 79.7%	0.695
> 8.41	100	67.6% to 100%	71.2	58.6% to 81.2%	0.712
> 8.49	100	67.6% to 100%	72.9	60.4% to 82.6%	0.729
> 9.26	100	67.6% to 100%	74.6	62.2% to 83.9%	0.746
> 10.1	100	67.6% to 100%	76.3	64.0% to 85.3%	0.763
> 10.3	100	67.6% to 100%	78	65.9% to 86.6%	0.780
> 11.0	100	67.6% to 100%	79.7	67.7% to 88.0%	0.797
> 11.6	100	67.6% to 100%	81.4	69.6% to 89.3%	0.814
> 12.0	100	67.6% to 100%	83.1	71.5% to 90.5%	0.831
> 12.5	100	67.6% to 100%	84.7	73.5% to 91.8%	0.847
> 13.2	100	67.6% to 100%	86.4	75.5% to 93.0%	0.864
> 15.1	100	67.6% to 100%	88.1	77.5% to 94.1%	0.881
> 20.4	100	67.6% to 100%	89.8	79.5% to 95.3%	0.898
> 26.2	100	67.6% to 100%	91.5	81.6% to 96.3%	0.915
> 32.8	100	67.6% to 100%	93.2	83.8% to 97.3%	0.932
> 37.3	100	67.6% to 100%	94.9	86.1% to 98.6%	0.949
> 42.1	100	67.6% to 100%	96.6	88.5% to 99.4%	0.966
> 49.9	100	67.6% to 100%	98.3	91.0% to 99.9%	0.983
**> 146**	**100**	**67.6% to 100%**	**100**	**93.9% to 100%**	**1,000**
> 1890	87.5	52.9% to 99.4%	100	93.9% to 100%	0.875
> 4659	75	40.9% to 95.6%	100	93.9% to 100%	0.750
> 7192	62.5	30.6% to 86.3%	100	93.9% to 100%	0.625
> 9690	50	21.5% to 78.5%	100	93.9% to 100%	0.500
> 11258	37.5	13.7% to 69.4%	100	93.9% to 100%	0.375
> 12093	25	4.44% to 59.1%	100	93.9% to 100%	0.250
> 39886	12.5	0.641% to 47.1%	100	93.9% to 100%	0.125
